# Synthesis of Novel Crown Ether-Squaramides and Their Application as Phase-Transfer Catalysts

**DOI:** 10.3390/molecules26216542

**Published:** 2021-10-29

**Authors:** Zsuzsanna Fehér, Dóra Richter, Sándor Nagy, Péter Bagi, Zsolt Rapi, András Simon, László Drahos, Péter Huszthy, Péter Bakó, József Kupai

**Affiliations:** 1 Department of Organic Chemistry and Technology, Budapest University of Technology and Economics, Szent Gellért tér 4, H-1111 Budapest, Hungary; zsuzsanna.feher@edu.bme.hu (Z.F.); richterdora98@gmail.com (D.R.); nagy.sandor@mail.bme.hu (S.N.); bagi.peter@vbk.bme.hu (P.B.); rapi.zsolt@vbk.bme.hu (Z.R.); huszthy.peter@vbk.bme.hu (P.H.); bako.peter@vbk.bme.hu (P.B.); 2 Department of Inorganic and Analytical Chemistry, Budapest University of Technology and Economics, Szent Gellért tér 4, H-1111 Budapest, Hungary; simon.andras@vbk.bme.hu; 3 MS Proteomics Research Group, Research Centre for Natural Sciences, Hungarian Academy of Sciences, Magyar Tudósok körútja 2, H-1117 Budapest, Hungary; drahos.laszlo@ttk.hu

**Keywords:** asymmetric catalysis, phase-transfer catalysis, enantioselectivity, allylation, crown compounds, carbohydrates, amino acids

## Abstract

This work presents the synthesis of six new phase-transfer organocatalysts in which the squaramide unit is directly linked to the nitrogen atom of an aza-crown ether. Four chiral skeletons, namely hydroquinine, quinine, cinchonine (cinchonas), and α-d-glucopyranoside were responsible for the asymmetric construction of an all-carbon quaternary stereogenic center in α-alkylation and Michael addition reactions of malonic esters. We investigated the effects of these different chiral units and that of crown ethers with different sizes on catalytic activity and enantioselectivity. During extensive parameter investigations, both conventional and emerging green solvents were screened, providing valuable α,α-disubstituted malonic ester derivatives with excellent yields (up to 98%).

## 1. Introduction

Investigating the creation of quaternary stereogenic centers is essential in organic synthesis as it poses a challenge to organic chemists due to the possible steric repulsion between the groups around the stereocenter. In the last decade, the synthesis of chiral α,α-disubstituted malonates has gained considerable attention [1,2]. These compounds can be applied in the preparation of α,α-disubstituted amino acids, as they can undergo chemoselective transformations if their carboxylic acid moieties are protected with different groups, e.g., through Curtius rearrangement [3]. A decade ago, a new method involving enantioselective phase-transfer catalytic double α-alkylation of malonates was developed for the construction of chiral quaternary carbon centers, applying *tert*-butyl diphenylmethyl α-alkylmalonates as starting materials [1]. This method was later extended in numerous publications by examining a broad substrate scope [3,4,5,6]. Moreover, similar phase-transfer catalytic reactions, such as the α-benzoyloxylation of *tert*-butyl methyl α-alkylmalonates [7] and the Michael addition reaction of *tert*-butyl methyl α-benzylmalonate to acrylates, have also been elaborated [2]. In all of these papers, quaternary ammonium salts were applied as phase-transfer catalysts (PTCs) in the enantioselective α-alkylation of malonates, while chiral macrocycle PTCs remain a rather neglected tool for these asymmetric reactions.

Phase-transfer catalysis is one of the most efficient asymmetric synthetic methods, which is also inexpensive and sustainable as it involves simple procedures and mild reaction conditions. Thanks to these advantages, phase-transfer catalysis is particularly suitable for industrial applications [8].

The most common types of asymmetric phase-transfer catalysts are chiral quaternary ammonium and phosphonium salts, but chiral crown ether derivatives and other macrocycles emerge as alternatives despite their cumbersome and costly synthesis [9,10,11]. Crown ethers are neutral ligands that can complex and transport alkali metal cations into the organic phase. This crown ether–metal cation complex plays the same role as the quaternary onium cation, but crown ethers work with a different mechanism, called cation-binding catalysis, during which the entire reacting ion pair is transported into the organic phase, not just the anion [12]. The most conspicuous benefits that help crown ether derivatives to stand out from other catalysts are as follows: they are usually more resistant to strong bases, and the cation is usually more accessible than the positively charged nitrogen or phosphorus atom in ammonium or phosphonium salts; consequently, a stronger interaction can take place with the reactive anion. Furthermore, crown ethers are more effective in extracting inorganic salts from their solid form [13].

In asymmetric phase-transfer catalysis, introducing hydrogen bond donor units into catalysts has recently attained broad application [14]. The most common of those units include the hydroxyl group, amide, (thio)urea and squaramide. In the case of quaternary onium salts, there are many examples for the installation of these units into catalyst scaffold [15,16,17,18,19,20]. However, in crown ether derivatives, only hydroxyl groups are frequently used as ancillary components capable of forming hydrogen bonds.

Squaramides are highly effective double hydrogen bond donor units due to their rigid, aromatic four-membered ring [21]. There are a few examples in which a squaramide unit was connected indirectly to crown ether derivatives [22,23,24,25,26,27]. However, these ligands have only been applied for ion pair transport so far.

Herein, we present the synthesis of novel chiral crown ether-squaramide phase-transfer organocatalysts, and the catalytic performance of these new derivatives were tested in the synthesis of α,α-disubstituted malonates. The effects of different chiral units and various cavity sizes of crown ethers on catalytic activity and enantioselectivity were investigated. In these catalysts, the squaramide unit is directly linked to the nitrogen atom of an aza-crown ether; thus, one amino group of the squaramide unit is tertiary. This means that the squaramide unit serves as a linker and a single hydrogen bond donor unit [28,29]. By applying this catalyst design, we have anticipated that one NH group and the cation complexed by the crown ether can form secondary interactions of sufficient strength during the catalysis.

## 2. Results and Discussion

### 2.1. Synthesis of Crown Ether-Squaramide Phase-Transfer Catalysts

**Scheme 1:**
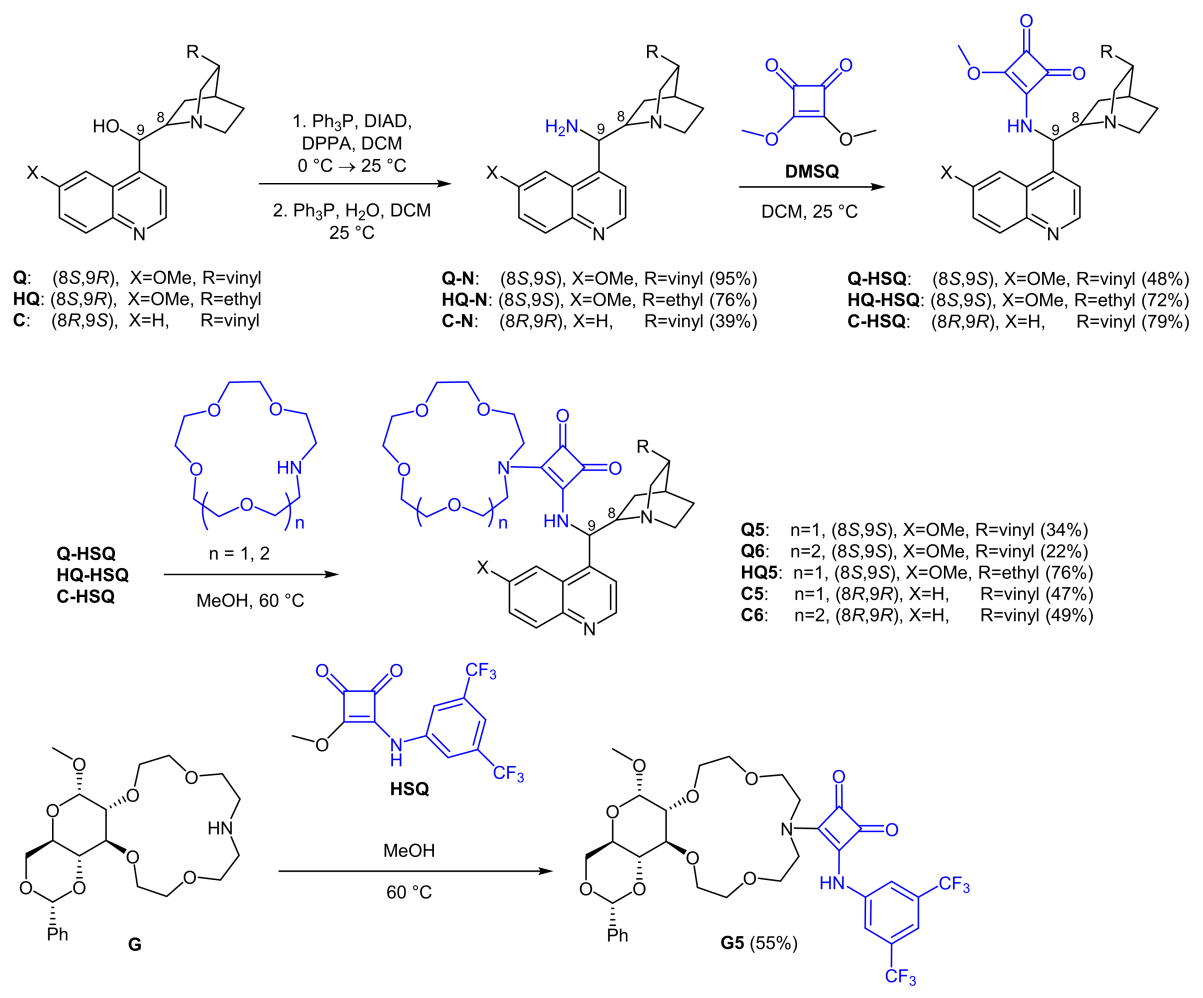
Synthesis of cinchona alkaloid-based (**Q5**, **Q6**, **HQ5**, **C5**, **C6**) and d-glucose-based (**G5**) crown ether-squaramide phase-transfer catalysts. DIAD: diisopropyl azodicarboxylate, DPPA: diphenylphosphoryl azide, DCM: dichloromethane.

We have prepared a series of crown ethers with a squaramide hydrogen bond donor unit (Scheme 1) for catalyzing the α-alkylation reaction of malonates, which may be an important method for obtaining α,α-disubstituted α-amino acid intermediates. Different cinchona alkaloids [quinine (**Q**), hydroquinine (**HQ**) and cinchonine (**C**)] and a d-glucose derivative (**G**) were chosen as chiral starting materials for the syntheses of the catalysts. Previously, we have shown that the substituent on the nitrogen atom of glucose-based aza-crown ether **G** influences the catalytic effect [30].

The cinchona alkaloid-based catalysts were prepared in 3 steps. The C9 hydroxyl group of the commercially available starting materials (**Q**, **HQ**, **C**) was converted to an amino group as reported earlier [31]. In the next step, half squaramide derivatives quinine-half squaramide (**Q-HSQ**), hydroquinine-half squaramide (**HQ-HSQ**) and cinchonine-half squaramide (**C-HSQ**) were obtained by the addition of these amines to dimethyl squarate (**DMSQ**) [32,33]. Finally, the addition of 1-aza-15-crown-5 or 1-aza-18-crown-6 ethers to the previously mentioned half squaramides afforded the corresponding cinchona-crown ether-squaramide derivatives (**Q5**, **Q6**, **C5**, **C6**, **HQ5**).

The glucose-based catalyst was prepared in a two-step-synthesis from glucose-aza-crown ether derivative **G**, which can be prepared in 5 steps from d-glucose [34,35]. First, dimethyl squarate (**DMSQ**) was reacted with 3,5-bis(trifluoromethyl)aniline to obtain a half squaramide derivative **HSQ** [36]; then, the treatment of aza-crown ether **G** with this half squaramide (**HSQ**) led to the appropriate glucose-crown ether-squaramide derivative (**G5**).

During the synthetic procedures, all compounds were characterized by well-established methods, including HRMS, IR, ^1^H and ^13^C NMR spectroscopies.

### 2.2. Application of the Catalysts

**Scheme 2:**
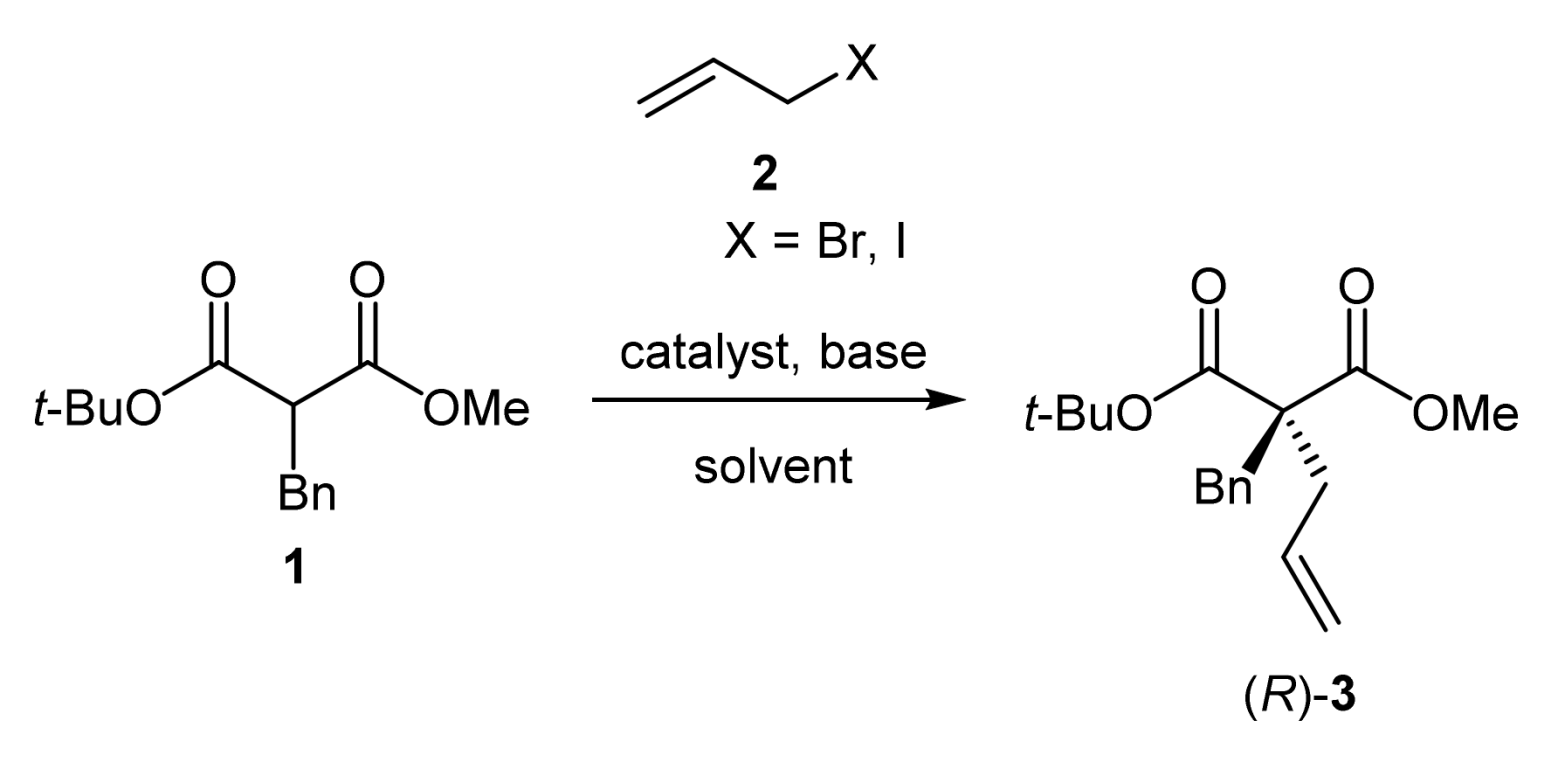
Synthesis of α,α-disubstituted malonate (*R*)-**3** by asymmetric *α*-alkylation of *tert*-butyl methyl α-benzylmalonate (**1**) with allyl halides **2** under phase-transfer conditions.

The catalysts were applied in the asymmetric α-alkylation of *tert*-butyl methyl α-benzylmalonate [3] (**1**) under phase-transfer conditions (Scheme 2).

First, we compared the activities and enantioselectivities of the catalysts; these results are shown in Table 1. The catalysts provided good yields except catalysts bearing a 1-aza-18-crown-6 ether macroring (**Q6** and **C6**, Table 1, Entries 5–6), and only poor or no enantioselectivity. The highest enantiomeric excess of (*R*)-**3** was obtained with catalyst **C5**, which was still as low as 9% (Table 1, Entry 3). In the absence of a base while applying **C5** as catalyst, no product was observed according to thin-layer chromatography (TLC) analysis. When 50% aq. NaOH was applied as a base in the absence of a phase-transfer catalyst, the yield was only 33%.

Next, we conducted a parameter study with catalyst **C5** to maximize the enantioselectivity. First, we examined the effect of the solvent while applying 50% aq. NaOH or solid NaOH as base (Table 2). In the case of aqueous NaOH, six different solvents were tested (Table 2, Entries 1–6); however, only the most suitable solvent (DCM: dichloromethane; Table 2, Entry 7) and two other polar aprotic solvents (MeCN; THF: tetrahydrofuran; Table 2, Entries 8–9) were used with solid NaOH. Dichloromethane proved to be the best solvent in terms of yield and enantiomeric excess by applying 50% aq. NaOH as base (Table 2, Entry 1, 73% yield, 9% ee).

Then, the effect of the quantity of the applied base or its concentration was investigated. By decreasing the quantity of the 50% aq. NaOH from 50 to 25 eq, the yield slightly increased (from 73% to 85%), but the enantiomeric excess did not change. By using 1 eq of 50% aq. NaOH or 50 eq of 30% aq. NaOH, both yield and enantioselectivity declined (from 73% yield and 9% ee to 26–28% yield and <5% ee).

In the next step of the parameter study, we examined the effect of the catalyst quantity (10 or 20 mol%), the reagent quantity (1 or 1.2 eq) and the solvent volume (1.2 or 2.4 mL) (Table 3). This set of experiments indicated that the application of 10 mol% catalyst, 1.2 eq of allyl bromide and 2.4 mL DCM is the most beneficial as it gave (*R*)-**3** in an increased yield (83%) with a slightly better enantiomeric excess (11%, Table 3, Entry 3).

The reaction was also carried out using allyl iodide instead of allyl bromide as a reagent under the same conditions as at the beginning of the study (1 eq reagent, 10 mol% **C5** catalyst, 50 eq 50% aq. NaOH base, DCM solvent, 25 °C, 24 h). In this case, a better yield (98% instead of 73%) and a slightly higher, but still low, enantiomeric excess (12% instead of 9%) were obtained. Consequently, we continued the study by applying allyl iodide.

Next, the reaction was carried out at 0 °C as an endeavor to improve enantioselectivity. As increasing the quantity of the reagent to 1.2 eq and decreasing the quantity of the base (50% aq. NaOH) to 25 eq seemed to be able to ameliorate yield and enantiomeric excess, we also varied these conditions in the following experiments (Table 4). By setting a lower reaction temperature, the enantiomeric excess did not improve significantly in any case. Based on these experiments, the yield was found to deteriorate when only 25 eq base is applied, while it does not depend on the temperature and the reagent quantity in the applied ranges. We also conducted the reaction at −78 °C to examine whether the enantioselectivity could be improved by setting a drastically lower reaction temperature, but no product (**3**) was formed based on TLC analysis.

Furthermore, the use of other bases did not improve the outcome: with 2 eq Na_2_CO_3_ in THF or MeCN solvent, there was no product at 0 °C, and by applying 50% aq. NaOH base in the cases of **Q6** and **C6**, and 50% aq. KOH in the cases of **Q5**, **HQ5**, **C5** and **G5** catalysts (to examine the effect of both bases with each catalyst), enantioselective alkylation occurred only with **C5** catalyst at 0 °C (17% ee, but only 47% yield, with 50% aq. KOH).

**Scheme 3:**
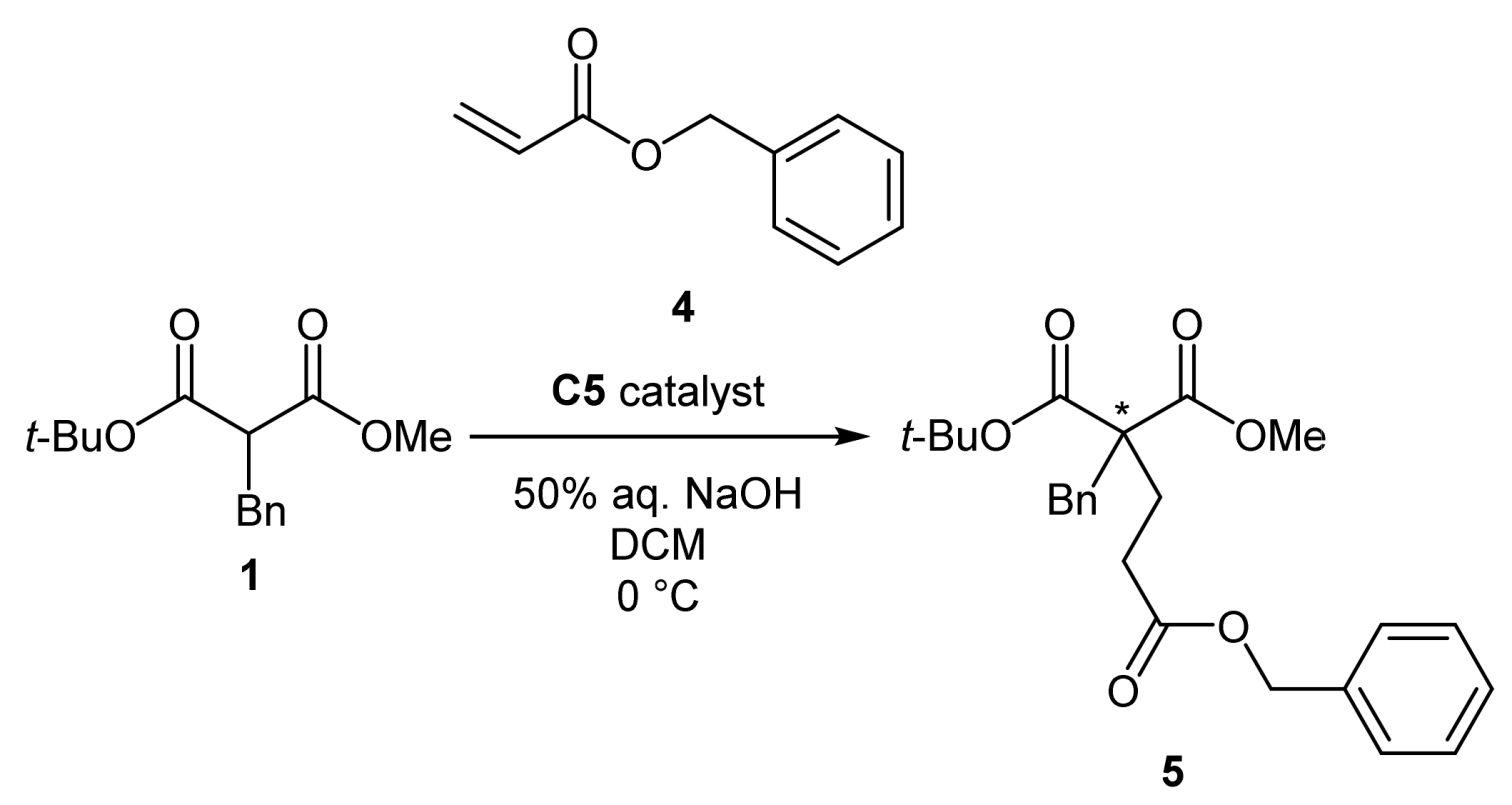
Synthesis of α,α-disubstituted malonate **5** by Michael addition of *tert*-butyl methyl α-benzylmalonate (**1**) to benzyl acrylate (**4**) (Reaction conditions: *tert*-butyl methyl α-benzylmalonate (1 eq), benzyl acrylate reagent (1.5 eq), **C5** catalyst (10 mol%), 50% aq. NaOH base (6.5 eq), DCM solvent (4 mL), 0 °C, 24 h reaction time).

As another attempt to increase enantioselectivity, we also investigated the activity and enantioselectivity of catalyst **C5** in Michael addition reaction of *tert*-butyl methyl α-benzylmalonate (**1**) to benzyl acrylate (Scheme 3). We anticipated higher enantioselectivity than in the alkylation reaction as benzyl acrylate has a hydrogen bond acceptor carbonyl oxygen, which could form stronger interactions with the hydrogen bond donor groups of the catalyst as opposed to the allyl halide reagents in the alkylation reaction. However, there was practically no enantioselectivity (2% ee) in this reaction, although product **5** was obtained in a good yield (74%).

### 2.3. Proposed Mechanism

**Scheme 4:**
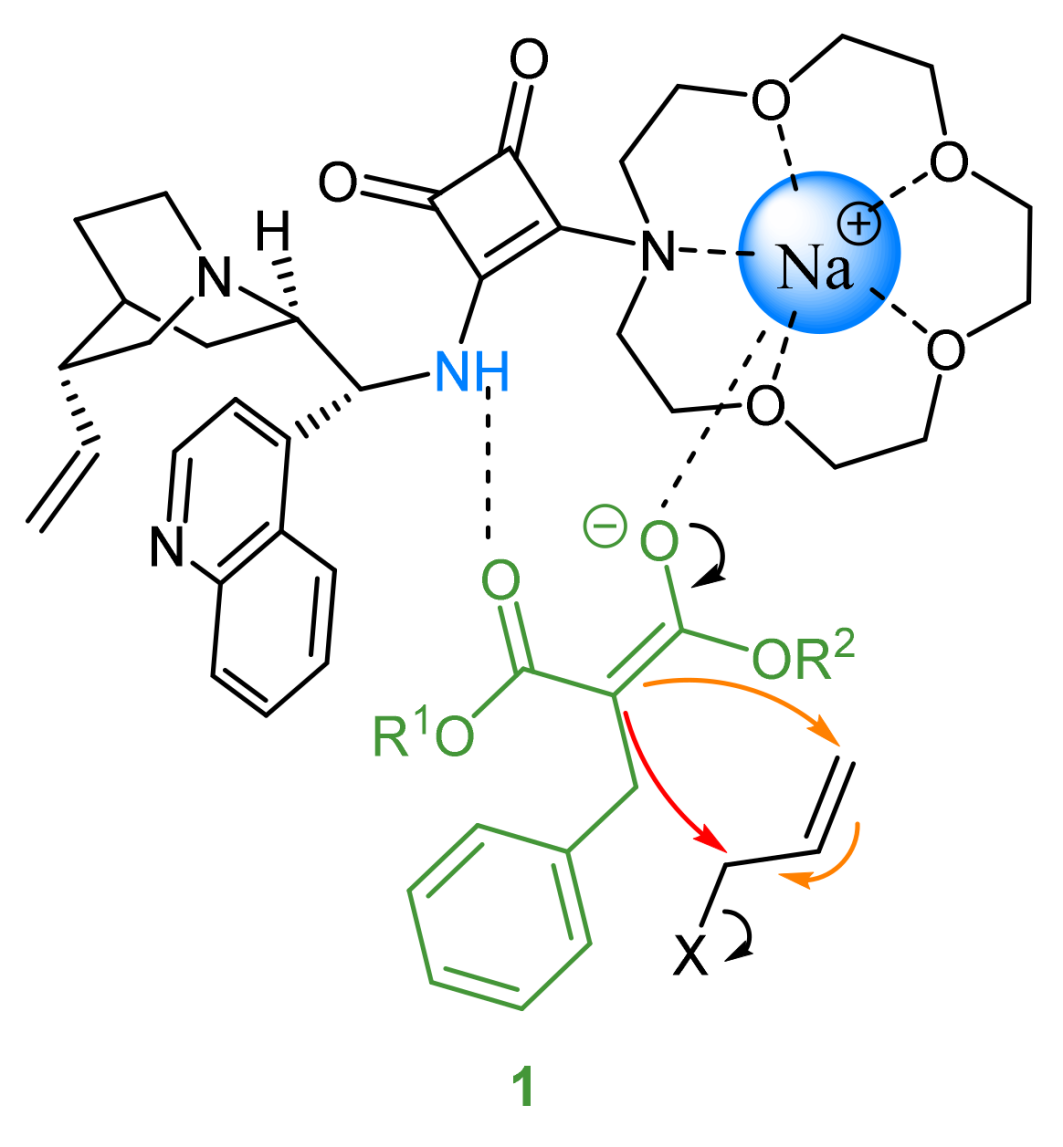
Proposed transition state for the *α*-alkylation reaction of *tert*-butyl methyl α-benzylmalonate (**1**) with catalyst **C5**. R^1^, R^2^ = *tert*-butyl or methyl.

Based on our previous research, we propose a mechanism for the α-alkylation reaction of *tert*-butyl methyl α-benzylmalonate (**1**) in the presence of our crown ether-squaramide catalysts. According to our preceding study, the Na^+^ ion of multiple monosaccharide-based aza-crown ethers’ Na^+^ complexes can be coordinated by a substrate’s carbonyl group [37]. In our other work, we have found that it is possible that only one NH group of a cinchona-based organocatalyst’s squaramide unit forms a hydrogen bond with a substrate’s carbonyl group [29]. Extending these results to our catalysts, we suggest that after the deprotonation of malonate **1** by the hydroxide ion, the phase-transfer catalyst coordinates the malonate anion in a way that one carbonyl of the malonate binds to the Na^+^ ion and the other to the NH group of the squaramide (Scheme 4). Then, the nucleophilic α-carbon of the malonate attacks the allyl halide, either directly on the electrophilic saturated carbon or in a conjugate addition on the unsaturated carbon, resulting in allylic rearrangement. Naturally, in the case of the applied unsubstituted allyl halides, the product is the same in both cases. Thus, the halide anion leaves, and an allyl group is built into the substrate.

## 3. Materials and Methods

### 3.1. General

Starting materials were purchased from commercially available sources (Sigma-Aldrich, Merck, and Alfa Aesar). Infrared spectra were recorded on a Bruker Alpha-T FT-IR spectrometer (Bruker, Ettlingen, Germany). Optical rotations were measured on a Perkin-Elmer 241 polarimeter (Perkin-Elmer, Waltham, MA, USA) that was calibrated by measuring the optical rotations of both enantiomers of menthol. Silica gel 60 F_254_ (Merck) plates were used for TLC. Silica gel 60 (70–230 mesh, Merck) was used for column chromatography. Ratios of solvents for the eluents are given in volumes (mL mL^−1^). Melting points were taken on a Boetius micro-melting point apparatus (VEB Dresden Analytik, Dresden, Germany), and they were uncorrected.

NMR spectra were recorded on a Bruker Avance 500 MHz spectrometer( Bruker, Ettlingen, Germany), (500.13 MHz for ^1^H, 125.76 MHz for ^13^C and 50.68 MHz for ^15^N) in DMSO-d_6_ and were referenced to residual solvent proton signals(δ_H_ = 2.50) and solvent carbon signals (δ_C_ = 39.51). The approximate ^15^N chemical shifts were detected by ^1^H,^15^N-gs-HMBC method optimized for *J*(N,H) = 5 Hz long-range couplings, and ^15^N chemical shifts are given on NH_3_ scale. The pulse programs of one-dimensional (^1^H, ^13^C and DEPTQ) and two-dimensional (^1^H,^1^H-gs-COSY, ^1^H,^13^C-gs-HSQC, ^1^H,^13^C-gs-HMQC, ^1^H,^13^C-gs-HMBC, ^1^H,^15^N-gs-HMBC and ^1^H,^1^H-gs-ROESY) measurements were utilized. All chemical shifts are reported in parts per million (ppm). Abbreviations used in the description of resonances are: a (methylene hydrogen with higher ^1^H chemical shift), b (methylene hydrogen with lower ^1^H chemical shift), o (overlapping signal), s (singlet), d (doublet), t (triplet), q (quartet), br (broad), m (multiplet) and nd: not detectable. Coupling constants (*J*) are given in Hz.

The broad signals, the non-integer integral values in the ^1^H NMR spectra (seen in Supplementary Materials) of compounds and the duplication of some signals in the ^1^H and ^13^C NMR spectra (e.g., ^1^H and ^13^C NMR signals of *N*-methylene groups in aza-crown ether units) of compounds indicate inhibited motions in the molecule. Also, the conformational properties of the molecules resulted in many cases in which not all signals appeared in the NMR spectra due to signal broadening. In these cases, the approximate chemical shift of the above-mentioned signals could be determined based on their HSQC or HMBC correlations. Previous ^1^H NMR measurements of similar compounds at higher temperatures showed that not all conformational motion inhibitions were removed.

In the process of structure elucidation, we assigned the ^1^H and ^13^C NMR signals of the main conformer with the exception of compound **G5**, in which the chemical shifts of several minor signals were also determined.

The starting points of signal assignment were easily identifiable units of molecules: the methyl and methoxy groups, the terminal olefinic methylene unit, the position 2′ in the quinoline ring and the methylidine groups next to nitrogen. The remainder of the molecular structures was elucidated using the same NMR methods mentioned above.

HPLC-MS was performed using a Shimadzu LCMS-2020 (Shimadzu Corp., Kyoto, Japan) device, equipped with a Reprospher (Altmann Analytik Corp., München, Germany) 100 C18 (5 µm; 100 × 3 mm) column and a positive/negative double ion source (DUIS±) with a quadrupole MS analyzer in a range of 50–1000 *m*/*z*. The samples were eluted with gradient elution, using eluent A (0.1% HCOOH in H_2_O) and eluent B (0.1% HCOOH in MeCN). The flow rate was set to 1.5 mL min^−1^. The initial condition was 5% eluent B, followed by a linear gradient to 100% eluent B by 1.5 min; from 1.5 to 4.0 min, 100% eluent B was retained; and from 4 to 4.5 min, it went back by a linear gradient to 5% eluent B, which was retained from 4.5 to 5 min. The column temperature was kept at room temperature, and the injection volume was 1 µL. The purity of the compounds was assessed by HPLC with UV detection at 215 and 254 nm. High-resolution mass measurements were performed on a Q-TOF Premier mass spectrometer (Waters Corp., Milford, MA, USA). The ionization method was electrospray ionization (ESI) operated in positive ion mode. The enantiomeric excess (ee) values were determined by chiral HPLC with a PerkinElmer Series 200 instrument (PerkinElmer, Waltham, MA, USA). The applied column, eluent, flow rate, column temperature, and detector wavelength are indicated at the corresponding procedure.

### 3.2. Procedures

#### 3.2.1. 3-(+)-Methoxy-4-(((R)-quinolin-4-yl((1S,2R,4S,5R)-5-vinylquinuclidin-2-yl)methyl)amino)cyclobut-3-ene-1,2-dione (**C-HSQ**)

A solution of **C-N** (535 mg, 1.55 mmol) in DCM (2 mL) was added to a solution of dimethyl squarate (**DMSQ**) (242 mg, 1.70 mmol) in DCM (2 mL) under argon atmosphere. This mixture was stirred for 3 h at room temperature. The solvent was removed under reduced pressure, then the crude product was purified by column chromatography (SiO_2_; DCM:methanol = 10:1) to obtain **C-HSQ** as pale-yellow crystals (494 mg, 79% yield). TLC (SiO_2_ TLC; DCM:methanol = 5:1, *R_f_* = 0.62). M.p. 122 °C (decomposed).${\left[\alpha \right]}_{D}^{25}$: +220.0 (*c* = 1.0, chloroform).

IR (KBr): 3233, 3075, 2938, 2869, 1803, 1706, 1606, 1510, 1460, 1393, 1375, 1263, 1240, 1174, 1079, 1053, 927, 851, 826, 768 cm^–1^.

^1^H NMR (500.13 MHz, DMSO-d_6_): δ = 9.20 (br m, 1H, NH), 8.94 (d, *J* = 3.8 Hz, 1H, H-2′), 8.35 (d, *J* = 7.9 Hz, 1H, H-5′), 8.07 (d, *J* = 8.2 Hz, 1H, H-8′), 7.80 (br m, 1H, H-7′), 7.72 (o m, 1H, H-6′), 7.71 (o m, 1H, H-3′), 6.08 (br m, 1H, H-9), 5.80 (br m, 1H, H-10), 5.13 (d, *J* = 17.1 Hz, 1H, H-11a), 5.08 (d, *J* = 10.5 Hz, 1H, H-11b), 4.25 (s, 3H, H-5′′), 3.28 (br m, 1H, H-8), 2.99 (o m, 1H, H-2a), 2.90 (o m, 1H, H-6a), 2.84 (o m, 1H, H-6b), 2.80 (o m, 1H, H-2b), 2.21 (br m, 1H, H-3), 1.51 (o m, 1H, H-4), 1.49 (o m, 2H, H-5ab), 0.91 (o m, 1H, H-7a), 0.84 (o m, 1H, H-7b).

^13^C NMR (125.76 MHz, DMSO-d_6_): δ = 189.8 (C-1′′ or C-2′′), 181.6 (C-1′′ or C-2′′), 177.5 (C-3′′), 171.2 (C-4′′), 150.4 (C-2′), 148.0 (C-8a′), 144.7 (C-4′), 140.6 (C-10), 130.1 (C-8′), 129.6 (C-7′), 127.3 (C-6′), 126.3 (C-4a′), 122.9 (C-5′), 119.8 (C-3′), 114.6 (C-11), 60.1 (C-5′′), 59.2 (C-8), 52.2 (C-9), 48.8 (C-6), 45.9 (C-2), 38.6 (C-3), 27.3 (C-4), 26.0 (C-5), 24.7 (C-7).

HRMS (ESI^+^): *m*/*z* [M + H]^+^ calcd for C_24_H_25_N_3_O_3_: 404.1974; found: 404.1974.

To the best of our knowledge, the synthesis of **C-HSQ** has not been reported so far.

#### 3.2.2. General Procedure for the Preparation of Cinchona Alkaloid-Based Catalysts

To a solution of cinchona half squaramide (**Q-HSQ** or **HQ-HSQ** or **C-HSQ**, 0.482 mmol) in methanol (2.1 mL), a solution of aza-crown ether (1-aza-15-crown-5 ether or 1-aza-18-crown-6 ether, 0.439 mmol) in methanol (2.1 mL) was added under argon atmosphere. The resulting mixture was warmed up to 60 °C, then it was stirred for 6 h at this temperature. The solvent was removed under reduced pressure, then the crude product was purified by column chromatography (SiO_2_; DCM:methanol = 10:1) to obtain **Q5** or **Q6** or **HQ5** or **C5** or **C6**.

#### 3.2.3. 3-(+)-(1,4,7,10-Tetraoxa-13-azacyclopentadecan-13-yl)-4-(((S)-(6-methoxyquinolin-4-yl)((1S,2S,4S,5R)-5-vinylquinuclidin-2-yl)methyl)amino)cyclobut-3-ene-1,2-dione (**Q5**)

Yellowish white crystals (93 mg, 34% yield). TLC (SiO_2_ TLC; DCM:methanol = 10:1, *R_f_* = 0.46). M.p. 97–100 °C. ${\left[\alpha \right]}_{D}^{25}$: +53.1 (*c* = 1.0, chloroform).

IR (KBr): 3323, 2934, 2863, 1786, 1664, 1621, 1516, 1471, 1432, 1354, 1313, 1298, 1245, 1230, 1176, 1124, 1082, 1057, 1028, 851 cm^–1^.

^1^H NMR (500.13 MHz, DMSO-d_6_): δ = 8.77 (d, *J* = 4.3 Hz, 1H, H-2′), 7.95 (d, *J* = 9.2 Hz, 1H, H-8′), 7.89 (br m, 1H, H-5′), 7.69 (d, *J* = 4.3 Hz, 1H, H-3′), 7.64 (br m, 1H,NH), 6.26 (br m, 1H, H-9), 7.42 (dd, *J* = 9.2, and 2.3 Hz, 1H, H-7′), 5.93 (ddd, *J* = 17.2, 10.3, and 7.6 Hz, 1H, H-10), 5.04 (d, *J* = 17.2 Hz, 1H, H-11a), 4.99 (d, *J* = 10.3 Hz, 1H, H-11b), 3.98 (br m, 1H, H-2′′′a or H-11′′′a), 3.93 (s, 3H, H-9′), 3.65 (br m, 2H, H-2′′′ and/or H-11′′′), 3.00–3.60 (o m, 16H, H-3′′′, H-4′′′, H-5′′′, H-6′′′, H-7′′′, H-8′′′, H-9′′′, and H-10′′′), 3.47 (o m, 1H, H-2′′′b or H-11′′′b), 3.50 (o m, 1H, H-8), 3.33 (o m, 1H, H-6a), 3.17 (o m, 1H, H-2a), 2.72 (dm, *J* = 13.3 Hz, 1H, H-2b), 2.62 (br m, 1H, H-6b), 2.27 (br m, 1H, H-3), 1.57 (br m, 1H, H-4), 1.49 (o m, 2H, H-5ab), 1.42 (tm, *J* = 12.2 Hz, 1H, H-7a), 0.55 (dm, *J* = 13.2, and 7.1 Hz, 1H, H-7b).

^13^C NMR (125.76 MHz, DMSO-d_6_): δ = 182.2 (C-1′′ or C-2′′), 181.8 (C-1′′ or C-2′′), 168.3 (C-3′′ or C-4′′), 166.5 (C-3′′ or C-4′′), 157.8 (C-6′), 144.2 (C-4′ or C-8a′), 144.0 (C-4′ or C-8a′), 147.8 (C-2′), 142.2 (C-10), 131.4 (C-8′), 127.7 (C-4a′), 121.9 (C-7′), 119.9 (C-3′), 114.4 (C-11), 101.8 (C-5′), 68.5–70.5 (C-3′′′, C-4′′′, C-5′′′, C-6′′′, C-7′′′, C-8′′′, C-9′′′, and C-10′′′), 58.6 (C-8), 55.7 (C-9′), 55.5 (C-2), 52.8 (C-9), 51.8 (C-2′′′ or C-13′′′), 51.4 (C-2′′′ or C-13′′′), 40.1 (C-6), 39.2 (C-3), 27.4 (C-5), 27.3 (C-4), 26.1 (C-7).

HRMS (ESI^+^): *m*/*z* [M + H]^+^ calcd for C_34_H_44_N_4_O_7_: 621.3288; found: 621.3285.

To the best of our knowledge, the synthesis of **Q5** has not been reported so far.

#### 3.2.4. 3-(+)-(1,4,7,10,13-Pentaoxa-16-azacyclooctadecan-16-yl)-4-(((S)-(6-methoxyquinolin-4-yl)((1S,2S,4S,5R)-5-vinylquinuclidin-2-yl)methyl)amino)cyclobut-3-ene-1,2-dione (**Q6**)

Pale-brown oil (64 mg, 22% yield). TLC (SiO_2_ TLC; DCM:methanol = 10:1, *R_f_* = 0.19). ${\left[\alpha \right]}_{D}^{25}$: +64.5 (*c* = 1.0, chloroform).

IR (KBr): 3311, 2909, 2867, 1784, 1666, 1622, 1574, 1521, 1473, 1431, 1347, 1315, 1303, 1248, 1231, 1134, 1107, 1028, 980, 833 cm^–1^.

^1^H NMR (500.13 MHz, DMSO-d_6_): δ = 8.78 (d, *J* = 4.3 Hz, 1H, H-2′), 7.96 (d, *J* = 9.1 Hz, 1H, H-8′), 7.90 (br m, 1H, H-5′), 7.71 (br m, 1H, H-3′), 7.66 (br m, 1H, NH), 6.32 (br m, 1H, H-9), 7.42 (dd, *J* = 9.1, and 2.2 Hz, 1H, H-7′), 5.95 (ddd, *J* = 17.3, 10.5, and 6.7 Hz, 1H, H-10), 5.08 (d, *J* = 17.3 Hz, 1H, H-11a), 5.03 (d, *J* = 10.5 Hz, 1H, H-11b), 3.99 (br m, 1H, H-2′′′a or H-11′′′a), 3.98 (br m, 1H, H-8), 3.93 (s, 3H, H-9′), 3.71 (br m, 2H, H-2′′′ and/or H-11′′′), 3.00–3.64 (o m, 18H, H-4′′′, H-5′′′, H-6′′′, H-7′′′, H-8′′′, H-9′′′, H-10′′′, H-11′′′, and H-12′′′), 3.52 (o m, 1H, H-2′′′b or H-11′′′b), 3.38 (o m, 1H, H-6a), 3.27 (o m, 1H, H-2a), 2.78 (br m, 1H, H-2b), 2.69 (br m, 1H, H-6b), 2.34 (br m, 1H, H-3), 1.62 (o m, 1H, H-4), 1.50 (o m, 1H, H-7a), 0.60 (br m, 1H, H-7b), and (2H, H-5).

^13^C NMR (125.76 MHz, DMSO-d_6_): δ = 182.1 (C-1′′ or C-2′′), 181.9 (C-1′′ or C-2′′), 168.2 (C-3′′ or C-4′′), 166.2 (C-3′′ or C-4′′), 157.9 (C-6′), 144.2 (C-8a′), 147.7 (C-2′), 142.0 (C-10), 131.4 (C-8′), 127.7 (C-4a′), 121.9 (C-7′), 120.0 (C-3′), 114.7 (C-11), 101.7 (C-5′), 68.5–70.5 (C-3′′′, C-4′′′, C-5′′′, C-6′′′, C-7′′′, C-8′′′, C-9′′′, C-10′′′, C-11′′′, and C-12′′′), 55.7 (C-8, C-9′), 55.3 (C-2), 52.6 (C-9), 50.6 (C-2′′′ or C-13′′′), 49.7 (C-2′′′ or C-13′′′), 40.2 (C-6), 38.9 (C-3), 27.7 (C-4), 25.8 (C-7), and (C-5, C-4′).

^15^N NMR (50.68 MHz, DMSO-d_6_): 312.3 (N-1′′).

HRMS (ESI^+^): *m*/*z* [M + H]^+^ calcd for C_36_H_48_N_4_O_8_: 665.3550; found: 665.3550.

To the best of our knowledge, the synthesis of **Q6** has not been reported so far.

#### 3.2.5. 3-(+)-(1,4,7,10-Tetraoxa-13-azacyclopentadecan-13-yl)-4-(((S)-((1S,2S,4S,5R)-5-ethylquinuclidin-2-yl)(6-methoxyquinolin-4-yl)methyl)amino)cyclobut-3-ene-1,2-dione (**HQ5**)

White powder (208 mg, 76% yield). TLC (SiO_2_ TLC; DCM:methanol = 10:1, *R_f_* = 0.19). M.p. 107 °C. ${\left[\alpha \right]}_{D}^{25}$: +50.8 (*c* = 1.0, chloroform).

IR (KBr): 3271, 2930, 2862, 1789, 1672, 1622, 1579, 1519, 1475, 1432, 1353, 1297, 1257, 1241, 1230, 1122, 1029, 939, 854, 831 cm^–1^.

^1^H NMR (500.13 MHz, DMSO-d_6_): δ = 8.78 (d, *J* = 4.3 Hz, 1H, H-2′), 7.95 (d, *J* = 9.2 Hz, 1H, H-8′), 7.89 (br, 1H, H-5′), 7.73 (br m, 1H, H-3′), 7.67 (br m, 1H,NH), 6.28 (br m, 1H, H-9), 7.42 (dd, *J* = 9.2, and 2.3 Hz, 1H, H-7′), 4.02 (br m, 1H, H-2′′′a or H-11′′′a), 3.93 (s, 3H, H-9′), 3.65 (br m, 2H, H-2′′′ and/or H-11′′′), 3.10–3.62 (o m, 14H, H-4′′′, H-5′′′, H-6′′′, H-7′′′, H-8′′′, H-9′′′, and H-10′′′), 3.57 (o m, 1H, H-8), 3.45 (o m, 1H, H-2′′′b or H-11′′′b), 3.36 (o m, 1H, H-6a), 3.20 (o m, 1H, H-2a), 2.67 (br m, 1H, H-6b), 2.51 (o m, 1H, H-2b), 1.58 (o m, 1H, H-4), 1.56 (o m, 1H, H-5a), 1.45 (o m, 1H, H-5b), 1.44 (o m, 1H, H-3), 1.41 (o m, 1H, H-7a), 1.34 (o m, 2H, H-10ab), 0.81 (t, *J* = 7.0 Hz, 3H, H-11), 0.58 (br m, 1H, H-7b).

^13^C NMR (125.76 MHz, DMSO-d_6_): δ = 182.2 (C-1′′ or C-2′′), 181.8 (C-1′′ or C-2′′), 168.4 (C-3′′ or C-4′′), 166.4 (C-3′′ or C-4′′), 157.8 (C-6′), 144.2 (C-8a′), 147.7 (C-2′), 131.4 (C-8′), 127.6 (C-4a′), 121.9 (C-7′), 119.9 (C-3′), 101.7 (C-5′), 69.0–70.0 (C-3′′′, C-4′′′, C-5′′′, C-6′′′, C-7′′′, C-8′′′, C-9′′′, and C-10′′′), 58.5 (C-8), 56.7 (C-2), 55.7 (C-9′), 52.4 (C-9), 51.8 (C-2′′′ or C-11′′′), 51.4 (C-2′′′ or C-11′′′), 39.8 (C-6), 36.5 (C-3), 27.6 (C-5), 27.0 (C-10), 25.5 (C-7), 24.8 (C-4), 11.9 (C-11), and (C-4′).

^15^N NMR (50.68 MHz, DMSO-d_6_): 312.1 (N-1′′).

HRMS (ESI^+^): *m*/*z* [M + H]^+^ calcd for C_34_H_46_N_4_O_7_: 623.3445; found: 623.3450.

To the best of our knowledge, the synthesis of **HQ5** has not been reported so far.

#### 3.2.6. 3-(+)-(1,4,7,10-Tetraoxa-13-azacyclopentadecan-13-yl)-4-(((R)-quinolin-4-yl((1S,2R,4S,5R)-5-vinylquinuclidin-2-yl)methyl)amino)cyclobut-3-ene-1,2-dione (**C5**)

White powder (122 mg, 47% yield). TLC (SiO_2_ TLC; DCM:methanol = 10:1, *R_f_* = 0.46). M.p. 204 °C. ${\left[\alpha \right]}_{D}^{25}$: +45.2 (*c* = 1.0, chloroform).

IR (KBr): 3308, 2934, 2895, 2856, 1790, 1664, 1575, 1526, 1475, 1433, 1384, 1352, 1317, 1265, 1249, 1124, 1080, 1058, 770 cm^–1^.

^1^H NMR (500.13 MHz, DMSO-d_6_): δ = 8.94 (d, *J* = 4.2 Hz, 1H, H-2′), 8.51 (d, *J* = 8.5 Hz, 1H, H-5′), 8.05 (d, *J* = 8.3 Hz, 1H, H-8′), 7.79 (o m, 1H, H-3′), 7.78 (o m, 1H, H-7′), 7.67 (o m, 1H, NH), 7.70 (o m, 1H, H-6′), 6.36 (br m, 1H, H-9), 5.81 (ddd, *J* = 17.3, 10.5, and 6.7 Hz, 1H, H-10), 5.17 (d, *J* = 17.3 Hz, 1H, H-11a), 5.08 (d, *J* = 10.5 Hz, 1H, H-11b), 3.93 (br m, 1H, H-2′′′a or H-11′′′a), 3.67 (br m, 2H, H-2′′′ and/or H-11′′′), 3.26–3.62 (o m, 14H, H-4′′′ H-5′′′ H-6′′′ H-7′′′ H-8′′′ H-9′′′ and H-10′′′), 3.58 (o m, 4H, H-3′′′ and H-10′′′), 3.52 (br m, 1H, H-2′′′b or H-11′′′b), 3.40 (o m, 1H, H-8), 3.14 (br m, 1H, H-2a), 2.96 (br m, 1H, H-6a), 2.88 (o m, 1H, H-6b), 2.82 (br m, 1H, H-2b), 2.23 (br m, 1H, H-3), 1.52 (o m, 1H, H-4), 1.51 (o m, 2H, H-5ab), 0.95 (br m, 1H, H-7a), 0.88 (br m, 1H, H-7b).

^13^C NMR (125.76 MHz, DMSO-d_6_): δ = 182.2 (C-1′′ or C-2′′), 182.1 (C-1′′ or C-2′′), 168.2 (C-3′′ or C-4′′), 166.8 (C-3′′ or C-4′′), 150.3 (C-2′), 148.0 (C-8a′), 145.7 (C-4′), 140.5 (C-10), 129.9 (C-8′), 129.4 (C-7′), 126.9 (C-6′), 126.6 (C-4a′), 123.5 (C-5′), 119.7 (C-3′), 114.6 (C-11), 69.0–70.0 (C-3′′′, C-4′′′, C-5′′′, C-6′′′, C-7′′′, C-8′′′, C-9′′′, and C-10′′′), 59.0 (C-8), 51.8 (C-9 and C-2′′′ or C-11′′′), 51.4 (C-2′′′ or C-11′′′), 48.8 (C-6), 45.8 (C-2), 38.6 (C-3), 27.3 (C-4), 25.9 (C-5), 24.9 (C-7).

^15^N NMR (50.68 MHz, DMSO-d_6_): 312.4 (N-1′′), 105.8 (9-NH, *^1^J* = 95 Hz).

HRMS (ESI^+^): *m*/*z* [M + H]^+^ calcd for C_33_H_42_N_4_O_6_: 591.3183; found: 591.3179.

To the best of our knowledge, the synthesis of **C5** has not been reported so far.

#### 3.2.7. 3-(+)-(1,4,7,10,13-Pentaoxa-16-azacyclooctadecan-16-yl)-4-(((R)-quinolin-4-yl((1S,2R,4S,5R)-5-vinylquinuclidin-2-yl)methyl)amino)cyclobut-3-ene-1,2-dione (**C6**)

Pale-brown oil (137 mg, 49% yield). TLC (SiO_2_ TLC; DCM:methanol = 5:1, *R_f_* = 0.62). ${\left[\alpha \right]}_{D}^{25}$: +25.2 (*c* = 1.0, chloroform).

IR (KBr): 3275, 2935, 2867, 1789, 1671, 1637, 1577, 1520, 1477, 1431, 1350, 1297, 1259, 1204, 1116, 987, 916, 852, 828, 771 cm^–1^.

^1^H NMR (500.13 MHz, DMSO-d_6_): δ = 8.94 (d, *J* = 4.0 Hz, 1H, H-2′), 8.52 (d, *J* = 8.3 Hz, 1H, H-5′), 8.06 (d, *J* = 8.3 Hz, 1H, H-8′), 7.80 (br m, 1H, H-3′), 7.78 (t, *J* = 7.7 Hz, 1H, H-7′), 7.68 (t, *J* = 7.4 Hz, 1H, H-6′), 7.65 (o m, 1H,NH), 6.41 (br m, 1H, H-9), 5.80 (ddd, *J* = 17.3, 10.4, and 6.3 Hz, 1H, H-10), 5.18 (d, *J* = 17.3 Hz, 1H, H-11a), 5.08 (d, *J* = 10.4 Hz, 1H, H-11b), 3.96 (br m, 1H, H-2′′′a or H-11′′′a), 3.71 (br m, 2H, H-2′′′ and/or H-11′′′), 3.25–3.64 (o m, 18H, H-4′′′, H-5′′′, H-6′′′, H-7′′′, H-8′′′, H-9′′′, H-10′′′, H-11′′′, and H-12′′′), 3.57 (o m, 1H, H-2′′′b or H-11′′′b), 3.47 (br m, 1H, H-8), 3.17 (o m, 1H, H-2a), 2.98 (o m, 1H, H-6a), 2.91 (o m, 1H, H-6b), 2.83 (o m, 1H, H-2b), 2.26 (br m, 1H, H-3), 1.56 (o m, 3H, H-4, H-5ab), 1.01 (br m, 1H, H-7a), 0.89 (br m, 1H, H-7b).

^13^C NMR (125.76 MHz, DMSO-d_6_): δ = 182.13 (C-1′′ or C-2′′), 182.11 (C-1′′ or C-2′′), 168.0 (C-3′′ or C-4′′), 166.6 (C-3′′ or C-4′′), 150.4 (C-2′), 145.5 (C-4′), 148.0 (C-8a′), 140.4 (C-10), 129.9 (C-8′), 129.5 (C-7′), 127.1 (C-6′), 126.6 (C-4a′), 123.6 (C-5′),119.9 (C-3′), 114.7 (C-11), 68.5–71.0 (C-3′′′, C-4′′′, C-5′′′, C-6′′′, C-7′′′, C-8′′′, C-9′′′, C-10′′′, C-11′′′, and C-12′′′), 58.9 (C-8), 52.0 (C-9), 51.6 (C-2′′′ or C-13′′′), 49.7 (C-2′′′ or C-13′′′), 48.8 (C-6), 45.8 (C-2), 38.6 (C-3), 27.2 (C-4), 25.8 (C-5), 25.0 (C-7).

HRMS (ESI^+^): *m*/*z* [M + H]^+^ calcd for C_35_H_46_N_4_O_7_: 635.3445; found: 635.3459.

To the best of our knowledge, the synthesis of **C6** has not been reported so far.

#### 3.2.8. Procedure for the Preparation of the Glucose-Based Catalyst: 3-(+)-((3,5-bis(trifluoromethyl)phenyl)amino)-4-((2R,4aR,6S,6aR,19aS,19bR)-6-methoxy-2-phenyltetradecahydro-13H-[1,3]dioxino[4′,5′:5,6]pyrano[3,4-e][1,4,7,10]tetraoxa[13]azacyclopentadecin-13-yl)cyclobut-3-ene-1,2-dione (**G5**)

To a solution of **HSQ** (119 mg, 0.350 mmol) in methanol (2.5 mL), a solution of crown ether derivative **G** (140 mg, 0.319 mmol) in methanol (2.5 mL) was added under argon atmosphere. The resulting mixture was warmed up to 60 °C, then stirred for 6 h at this temperature. The solvent was removed under reduced pressure, then the crude product was purified by column chromatography (SiO_2_; DCM:methanol = 40:1) to obtain **G5** as a yellowish-white powder (131 mg, 55% yield). TLC (SiO_2_ TLC; DCM:methanol = 40:1, *R_f_* = 0.19). M.p. 209–211 °C. ${\left[\alpha \right]}_{D}^{25}$: +32.5 (*c* = 1.0, chloroform).

IR (KBr): 3262, 3080, 2931, 2868, 1785, 1687, 1593, 1562, 1475, 1458, 1427, 1383, 1278, 1174, 1093, 1059, 1018, 992, 885, 766 cm^–1^.

^1^H NMR (500.13 MHz, DMSO-d_6_):

Major signals: δ = 9.55 (br s, 0.6H, NH), 7.92 (s, 2H, H-2′′′ and H-6′′′), 7.56 (br m, 0.55H, H-4′′′), 7.41 (o m, 2H, H-2′′ and H-6′′), 7.37 (o m, 2H, H-3′′ and H-5′′), 7.36 (o m, 1H, H-4′′), 5.61 (s, 0.65H, H-2), 4.92 (br m, 0.55H, H-6), 4.16 (o m, 2H, H-4ab), 3.30–3.90 (o m, 12H, H-8, H-9, H-10, H-15, H-17 and H-18), 3.55 (o m, 2H, H-4′′′ and H-5′′′), 3.52 (o m, 1H, H-19a), 3.35 (o m, 1H, H-6a), 3.31 (o s, 3H, CH_3_).

Minor signals: δ = 7.65 (br m, 0.45H, H-4′′′), 7.38 (o m, H-3′′ and H-5′′), 7.35 (o m, 1H, H-4′′), 7.32 (o m, H-3′′ and H-5′′), 7.27 (o m, H-2′′ and H-6′′), 5.45 (s, 0.5H, H-2), 3.24 (s, 1.7H, CH_3_), 3.19 (s, 0.5H, CH_3_)

^13^C NMR (125.76 MHz, DMSO-d_6_):

Major signals: δ = 185.7 (C-1′ or C-2′), 180.9 (C-1′ or C-2′), 171.1 (C-3′ or C-4′), 162.4 (C-3′ or C-4′), 141.0 (C-1′′′), 137.6 (C-1′′), 130.8 (q, *J* = 32.8 Hz, C-3′′′ and C-5′′′), 128.8 (C-4′′), 128.0 (C-3′′ and C-5′′), 126.1 (C-2′′ and C-6′′), 123.3 (q, *J* = 272.5 Hz, CF_3_), 119.1 (C-2′′′ and C-6′′′), 114.6 (C-4′′′), 100.5 (C-2), 97.3 (C-6), 81.1 (C-19b), 78.7 (C-6a), 77.8 (C-19a), 68.3–72.0 (C-8, C-9, C-11, C-15, C-17 and C-18), 68.1 (C-4), 62.0 (C-4a), 54.6 (CH_3_), 52.0 (C-2′′ or C-11′′), 51.7 (C-12 or C-14).

Minor signals: δ = 137.8 (C-1′′), 137.7 (C-1′′), 128.7 (C-4′′), 128.15 (C-3′′ and C-5′′), 128.12 (C-3′′ and C-5′′), 125.9 (C-2′′ and C-6′′), 119.1 (C-2′′′ and C-6′′′), 114.6 (C-4′′′), 100.5 (C-2), 98.4 (C-6), 97.8 (C-6), 80.8 (C-19b), 78.8 (C-6a), 62.2 (C-4a), 58.1 (CH_3_), 58.0 (CH_3_), 54.7 (CH_3_).

HRMS (ESI^+^): *m*/*z* [M + H]^+^ calcd for C_34_H_36_F_6_N_2_O_10_: 747.2352; found: 747.2355.

To the best of our knowledge, the synthesis of **G5** has not been reported so far.

#### 3.2.9. General Procedure for the α-alkylation of tert-butyl methyl α-benzylmalonate with allyl halides (**3**)

To a solution of *tert*-butyl methyl α-benzylmalonate (**1**, 26 mg, 0.1 mmol) in the indicated solvent, catalyst **Q5** or **Q6** or **HQ5** or **C5** or **C6** or **G5**, and a base were added (exact reaction conditions are shown in Table 1, Table 2, Table 3 and Table 4). Then, the allyl halide (**2**, allyl bromide or allyl iodide) was added, and the resulting mixture was stirred vigorously at the temperature shown in Table 1, Table 2, Table 3 and Table 4. After 24 h, water (1 mL) was added to the reaction mixture, and it was extracted with DCM (2 × 1 mL). The combined organic phase was dried over MgSO_4_ and filtered (except in the cases of MeCN and THF solvents, when the mixture was only dried over MgSO_4_, then filtered). The volatile components were removed under reduced pressure. The residue was purified by preparative TLC (SiO_2_; hexan:EtOAc = 4:1) to obtain product **3** as a colorless oil. Yields and enantiomeric excess (ee) values can be seen in Table 1, Table 2, Table 3 and Table 4. Spectroscopic data are fully consistent with those reported in the literature [3]. TLC (SiO_2_ TLC; hexane:EtOAc = 10:1, *R_f_* = 0.47). ${\left[\alpha \right]}_{D}^{25}$: –1.7 (*c* = 1.0, chloroform, 15% ee, (*R*) abs. config.) (lit. ${\left[\alpha \right]}_{D}^{25}$: –7.6 (*c* = 1.0, chloroform, 94% ee, (*R*) abs. config.). The enantiomeric excess values were determined by chiral HPLC using a Phenomenex Lux^®^ 5-µm Cellulose-2 column (250 × 4.6 mm ID), and a 95:5 mixture of hexane/ethanol was used as the eluent with a flow rate of 0.8 mL min^−1^. The column temperature was 20 °C. UV detector λ = 254 nm. Retention time for (*S*)-**3**: 5.0 min, for (*R*)-**3**: 5.4 min.

MS (ESI^+^): *m*/*z* (%) = 249 (100) [M-*t*-Bu + 2H]^+^.

#### 3.2.10. General Procedure for the Michael Addition of tert-butyl methyl α-benzylmalonate to benzyl acrylate (**5**)

To a solution of *tert*-butyl methyl α-benzylmalonate (**1**, 26 mg, 0.1 mmol) in DCM (4 mL), catalyst **C5** (6 mg, 0.01 mmol) and aq. 50% NaOH (35 µL, 0.65 mmol) were added. Then, benzyl acrylate (**4**, 23 µL 24 mg, 0.15 mmol) was added, and the resulting mixture was stirred vigorously at 0 °C. After 24 h, water (1 mL) was added to the reaction mixture, then it was extracted with DCM (2 × 1 mL), dried over MgSO_4_, and filtered. The volatile components were removed under reduced pressure. The residue was purified by preparative TLC (SiO_2_; hexane:EtOAc = 10:1) to obtain product **5** (32 mg, 74% yield, 2% ee) as a colorless oil. Spectroscopic data are fully consistent with those reported in the literature [2]. TLC (SiO_2_ TLC; hexane:EtOAc = 10:1, *R_f_* = 0.23). In this case, the enantiomeric excess values were determined by chiral HPLC using a Kromasil^®^ 5-µm AmyCoat^®^ column (250 × 4.6 mm ID), an 85:15 mixture of hexane/ethanol was used as the eluent with a flow rate of 0.8 mL min^−1^. The column temperature was 20 °C. UV detector λ = 254 nm. Retention times: 6.8 min and 8.7 min.

MS (ESI^+^): *m*/*z* (%) = 371 (100) [M-*t*-Bu + 2H]^+^.

## 4. Conclusions

In conclusion, we have described the synthesis of six new crown ether-squaramide phase-transfer organocatalysts bearing four different chiral units. To the best of our knowledge, this is the first successful direct coupling of squaramide and aza-crown-ether units and the first application of crown ether-squaramides as phase-transfer catalysts. We have tested their performance in the asymmetric α-alkylation of *tert*-butyl methyl α-benzylmalonate with extensive parameter investigation, after which reaction of the products could be converted to α,α-disubstituted amino acids through Curtius rearrangement. The alkylation reactions afforded the products in excellent yields, but with only low enantiomeric excess values. Thus, despite the low enantioselectivity, the new crown ether-based catalysts can catalyze the often-difficult construction of quaternary carbon centers. With the catalyst having the best enantioselectivity, a Michael addition reaction of *tert*-butyl methyl α-benzylmalonate was also conducted; however, no enantioselectivity was observed. Based on our results, we anticipate that a linker between the crown ether and the squaramide unit may potentially increase the enantioselectivity of this new type of phase-transfer catalyst, as two NH groups may form stronger hydrogen bonds during catalysis.

## Figures and Tables

**Table 1:** Comparison of the catalysts in the α-alkylation of malonate **1** ^a^.

Entry	Catalyst	Base ^b^	Yield ^c^ (%)	ee ^d^ (%)
1	**Q5**	50% aq. NaOH	98	5
2	**HQ5**	50% aq. NaOH	93	<5
3	**C5**	50% aq. NaOH	73	9
4	**G5**	50% aq. NaOH	86	5
5	**Q6**	50% aq. KOH	59	<5
6	**C6**	50% aq. KOH	37	<5

^a^ Reaction conditions: *tert*-butyl methyl α-benzylmalonate (1 eq), allyl bromide reagent (1 eq), catalyst (10 mol%), base (50 eq), 2.4 mL dichloromethane (DCM) solvent, 25 °C, 24 h reaction time. ^b^ Applied base chosen considering the phase-transfer catalyst (PTC) crown ether cavity size. ^c^ Yield of isolated product purified by preparative thin-layer chromatography (TLC). ^d^ Determined by chiral high-performance liquid chromatography (HPLC).

**Table 2:** Examination of the effect of the solvent in the presence of catalyst **C5** ^a^.

Entry	Solvent	Base	Base Quantity (eq)	Yield ^b^ (%)	ee ^c^ (%)
1	DCM	50% aq. NaOH	50	73	9
2	toluene	50% aq. NaOH	50	60	7
3	EtOAc	50% aq. NaOH	50	65	5
4	MTBE	50% aq. NaOH	50	51	<5
5	CPME	50% aq. NaOH	50	49	<5
6	2-Me-THF	50% aq. NaOH	50	51	<5
7	DCM	solid NaOH	1	38	10
8	MeCN	solid NaOH	1	49	<5
9	THF	solid NaOH	1	38	<5

^a^ Reaction conditions: *tert*-butyl methyl α-benzylmalonate (1 eq), allyl bromide reagent (1 eq), **C5** catalyst (10 mol%), base, 2.4 mL solvent, 25 °C, 24 h reaction time. ^b^ Yield of isolated product purified by preparative TLC. ^c^ Determined by chiral HPLC. ee: enantiomeric excess, DCM: dichloromethane, MTBE: *tert*-butyl methyl ether, CPME: cyclopentyl methyl ether, 2-Me-THF: 2-methyltetrahydrofuran, THF: tetrahydrofuran.

**Table 3:** Examination of the effect of catalyst quantity, reagent quantity and solvent volume in the presence of catalyst **C5** ^a^.

Entry	Catalyst Quantity (mol%)	Allyl Bromide Quantity (eq)	Solvent Volume (mL)	Yield ^b^ (%)	ee ^c^ (%)
1	10	1	2.4	73	9
2	20	1	2.4	79	8
3	10	1.2	2.4	83	11
4	10	1	1.2	92	6

^a^ Reaction conditions: *tert*-butyl methyl α-benzylmalonate (1 eq), allyl bromide reagent, **C5** catalyst, 50% aq. NaOH base (50 eq), DCM solvent, 25 °C, 24 h reaction time. ^b^ Yield of isolated product purified by preparative TLC. ^c^ Determined by chiral HPLC. ee: enantiomeric excess.

**Table 4:** Examination of the effect of temperature and quantities of reagent and base in the presence of catalyst **C5** ^a^.

Entry	Temperature (°C)	Reagent Quantity (eq)	Base Quantity (eq)	Yield ^b^ (%)	ee ^c^ (%)
1	25	1	50	98	12
2	0	1	50	94	15
3	0	1	25	79	15
4	0	1.2	50	97	15
5	0	1.2	25	74	15

^a^ Reaction conditions: *tert*-butyl methyl α-benzylmalonate (1 eq), allyl iodide reagent, **C5** catalyst (10 mol%), 50% aq. NaOH base, 2.4 mL DCM solvent, 24 h reaction time. ^b^ Yield of isolated product purified by preparative TLC. ^c^ Determined by chiral HPLC. ee: enantiomeric excess.

## Data Availability

Not applicable.

## Supplementary Materials

The following are available online: NMR spectra of the new compounds and HPLC chromatograms of products **3** and **5**.

## Sample Availability

Samples of the compounds are available from the authors.
