# Supplementary material for: Synthesis of Novel Crown Ether-Squaramides and Their Application as Phase-Transfer Catalysts
Source: Molecules. 2021 Oct 29;26(21):6542. doi: 10.3390/molecules26216542 (PMC8588334; doi:10.3390/molecules26216542)
Supplement: Supplementary file 1 [file molecules-26-06542-s001.zip › molecules-1401596-supplementary.pdf]

Synthesis of Novel Crown Ether-Squaramides and Their Application as Phase-Transfer Catalysts

Zsuzsanna Fehér [1], Dóra Richter [1], Sándor Nagy [1], Péter Bagi [1], Zsolt Rapi [1], András Simon [2], László Drahos [3], Péter Huszthy [1], Péter Bakó [1] and József Kupai [1,*]

[1] Department of Organic Chemistry and Technology, Budapest University of Technology and Economics, Szent Gellért tér 4, H-1111 Budapest, Hungary; zsuzsanna.feher@edu.bme.hu (Z.F.); richterdora98@gmail.com (D.R.); nagy.sandor@mail.bme.hu (S.N.); bagi.peter@vbk.bme.hu (P.B.); rapi.zsolt@vbk.bme.hu (Z.R.); huszthy.peter@vbk.bme.hu (P.H.); bako.peter@vbk.bme.hu (P.B.)
[2] Department of Inorganic and Analytical Chemistry, Budapest University of Technology and Economics, Szent Gellért tér 4, H-1111 Budapest, Hungary; simon.andras@vbk.bme.hu
[3] MS Proteomics Research Group, Research Centre for Natural Sciences, Hungarian Academy of Sciences, Magyar Tudósok körútja 2, H-1117 Budapest, Hungary; drahos.laszlo@ttk.hu
* Correspondence: kupai.jozsef@vbk.bme.hu; Tel.: +36-14-632-369
https://www.kupaigroup.com/

Citation: Fehér, Z.; Richter, D.; Nagy, S.; Bagi, P.; Rapi, Z.; Simon, A.; Drahos, L.; Huszthy, P.; Bakó, P.; Kupai, J. Synthesis of Novel Crown Ether-Squaramides and Their Application as Phase-Transfer Catalysts. *Molecules* **2021**, *26*, 6542. https://doi.org/10.3390/molecules26216542

Academic Editors: Dimitrios Bikiaris and Maurizio Benaglia

Received: 15 September 2021
Accepted: 27 October 2021
Published: 29 October 2021

Publisher's Note: MDPI stays neutral with regard to jurisdictional claims in published maps and institutional affiliations.

Copyright: © 2021 by the authors. Licensee MDPI, Basel, Switzerland. This article is an open access article distributed under the terms and conditions of the Creative Commons Attribution (CC BY) license (http://creativecommons.org/licenses/by/4.0/).

Supporting Information

1. NMR spectra 2
NMR spectra of intermediate **C-HSQ** 2
NMR spectra of catalyst **C5** 8
NMR spectra of catalyst **C6** 14
NMR spectra of catalyst **Q5** 19
NMR spectra of catalyst **Q6** 25
NMR spectra of catalyst **HQ5** 30
NMR spectra of catalyst **G5** 35
2. HPLC chromatograms 41
Product **3** of α-alkylation reaction 41
Product **5** of *Michael* addition reaction 43

1. NMR spectra

NMR spectra of intermediate **C-HSQ**

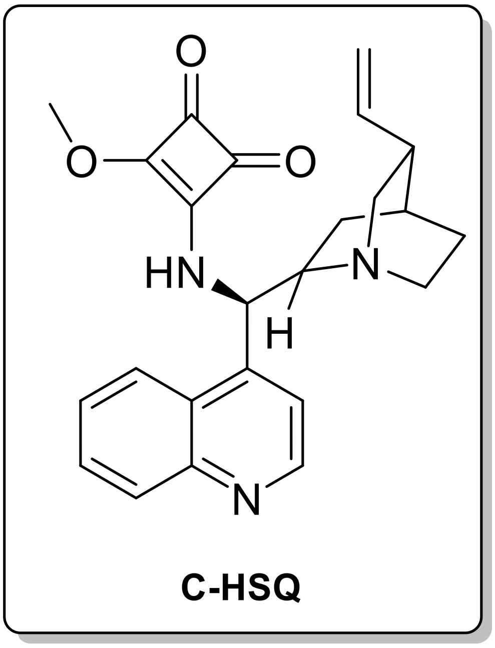

Figure S1. Molecule structure of intermediate **C-HSQ**

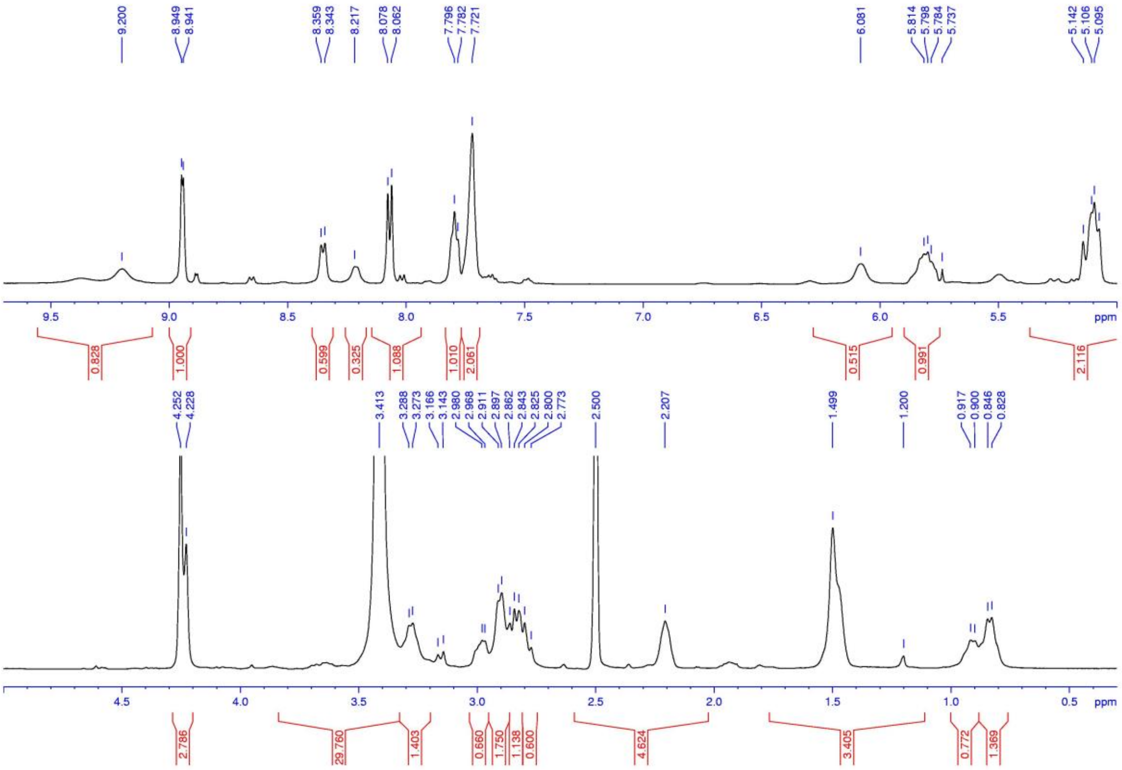

Figure S2. ^{1}H NMR spectrum of intermediate **C-HSQ** (500.13 MHz, DMSO-d_6)

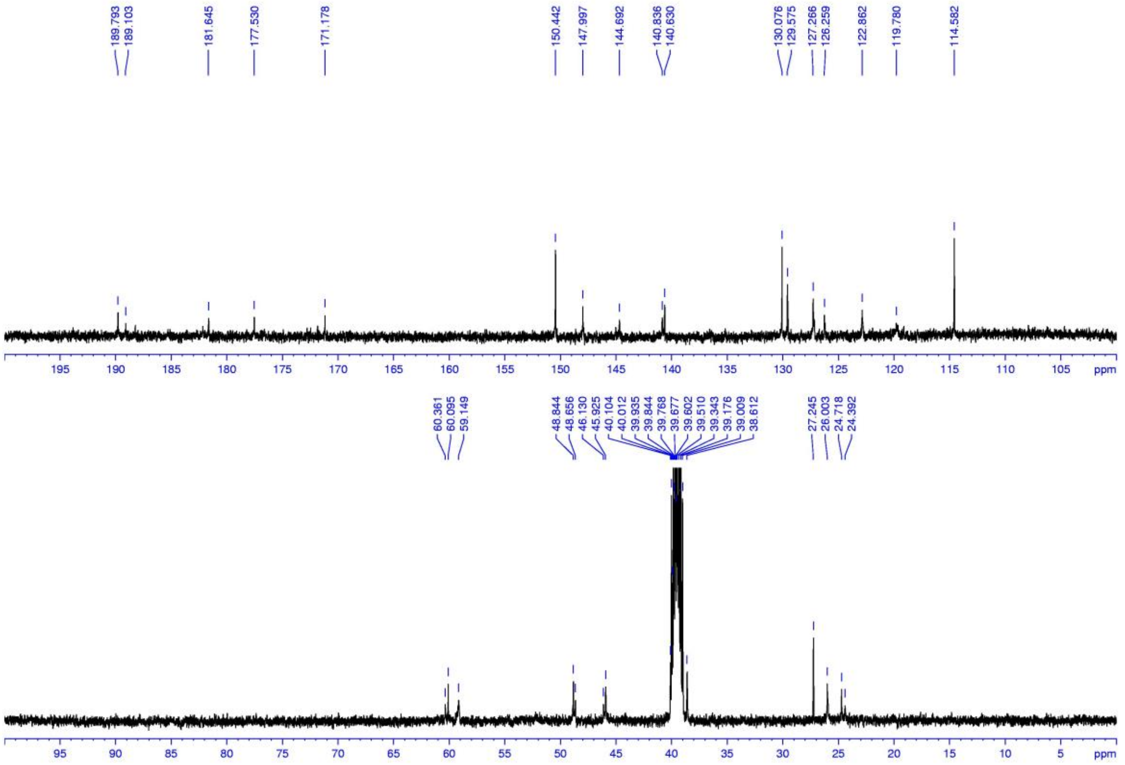

Figure S3. ^{13}C NMR spectrum of intermediate **C-HSQ** (125.76 MHz, DMSO-d_6)

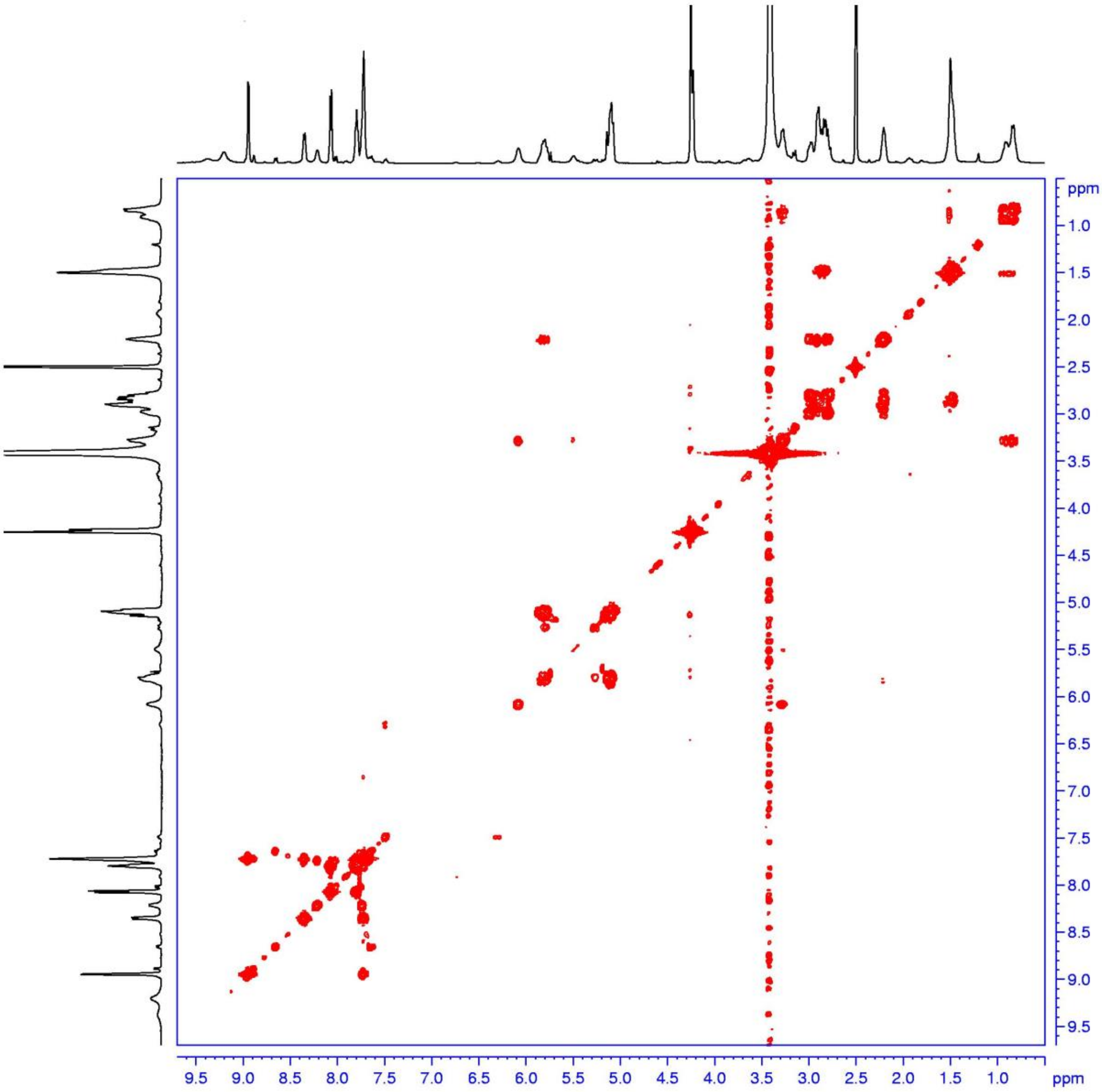

Figure S4. ^{1}H-gs-COSY NMR spectrum of intermediate **C-HSQ** (DMSO-d_6)

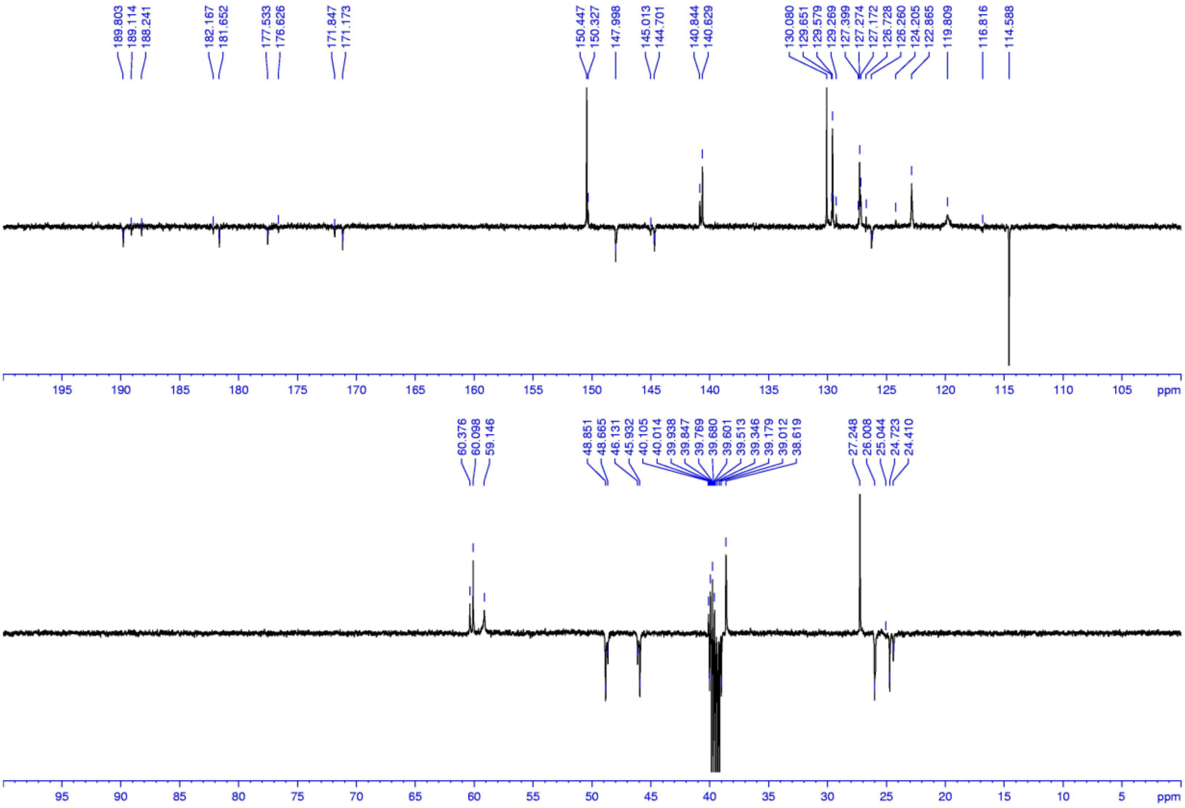

Figure S5. ^{13}C DEPTQ NMR spectrum of intermediate **C-HSQ** (DMSO-d_6)

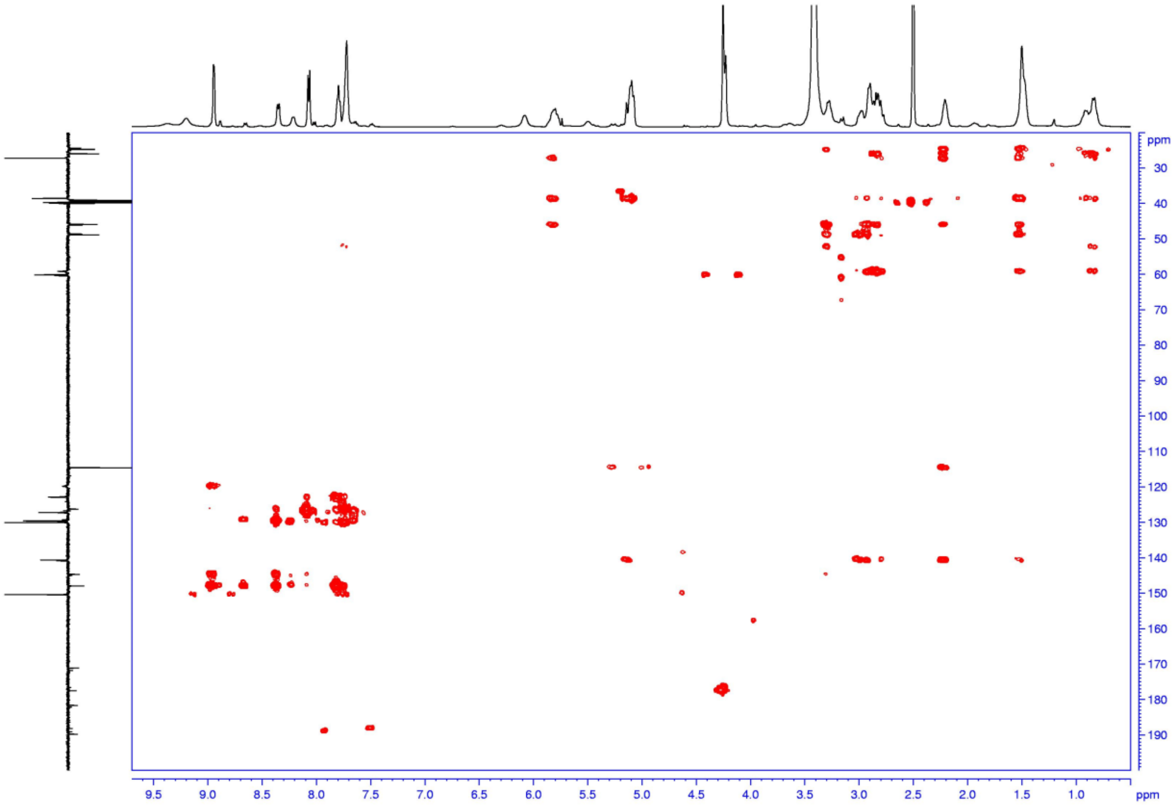

Figure S6. ^{1}H,^{13}C-gs-HMBC NMR spectrum of intermediate **C-HSQ** (DMSO-d_6)

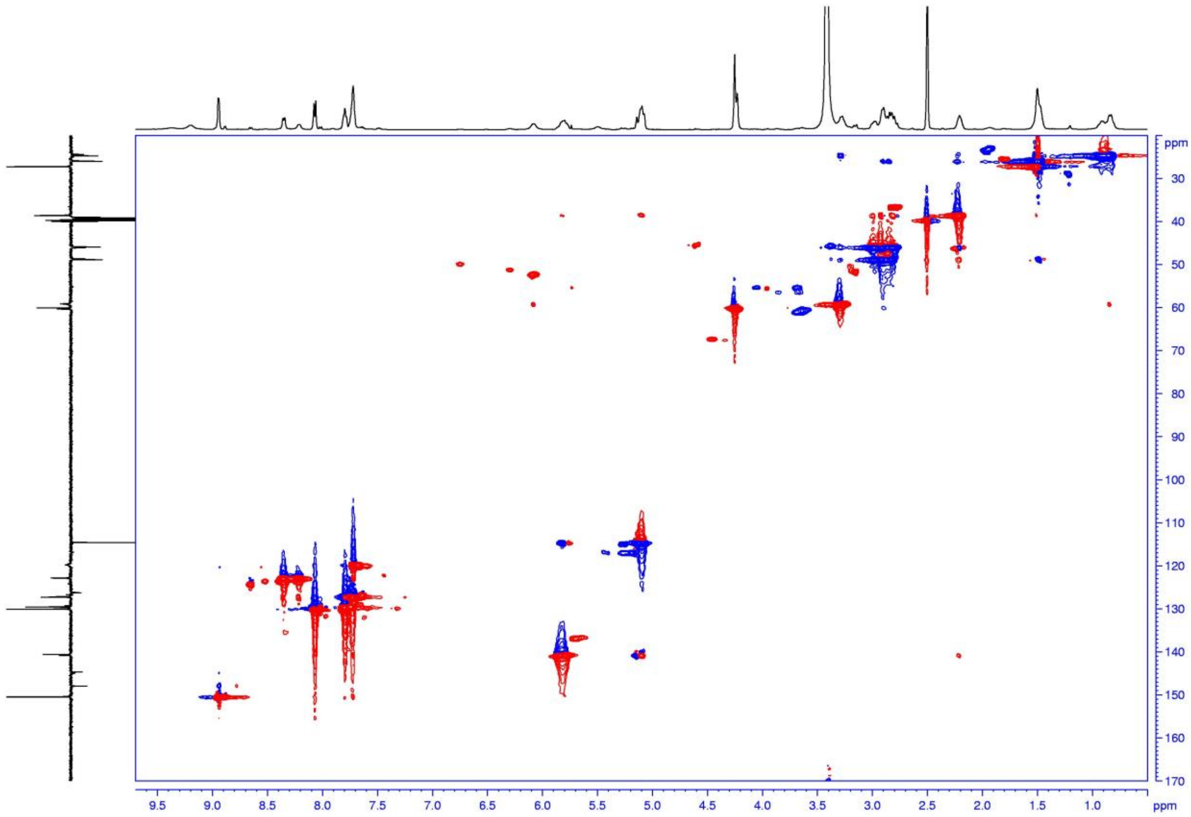

Figure S7. $^{1}H,^{13}C$-gs-HSQC NMR spectrum of intermediate **C-HSQ** (DMSO-d_6)

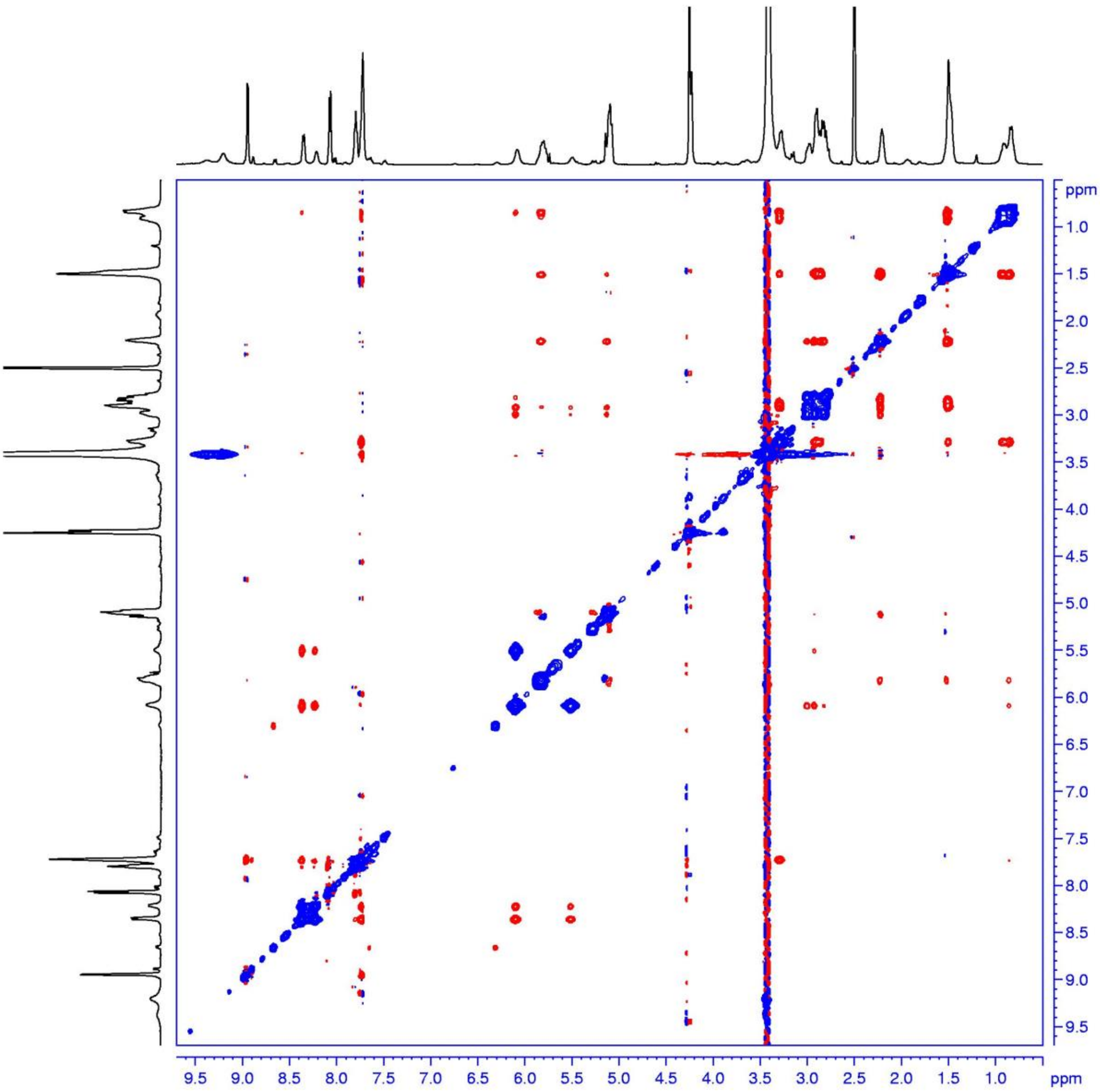

Figure S8. ^{1}H,^{1}H-gs-ROESY NMR spectrum of intermediate **C-HSQ** (DMSO-d$_6$)

NMR spectra of catalyst **C5**

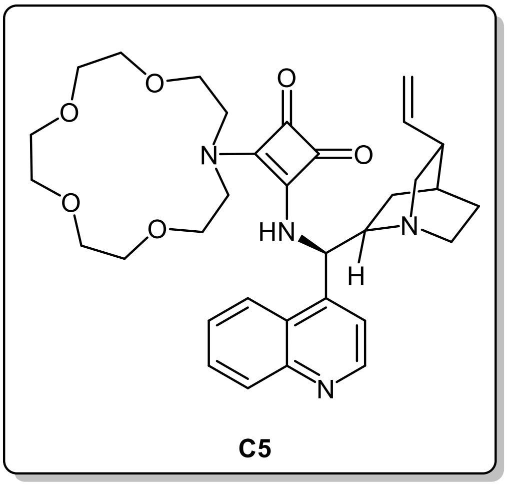

Figure S9. Molecule structure of catalyst **C5**

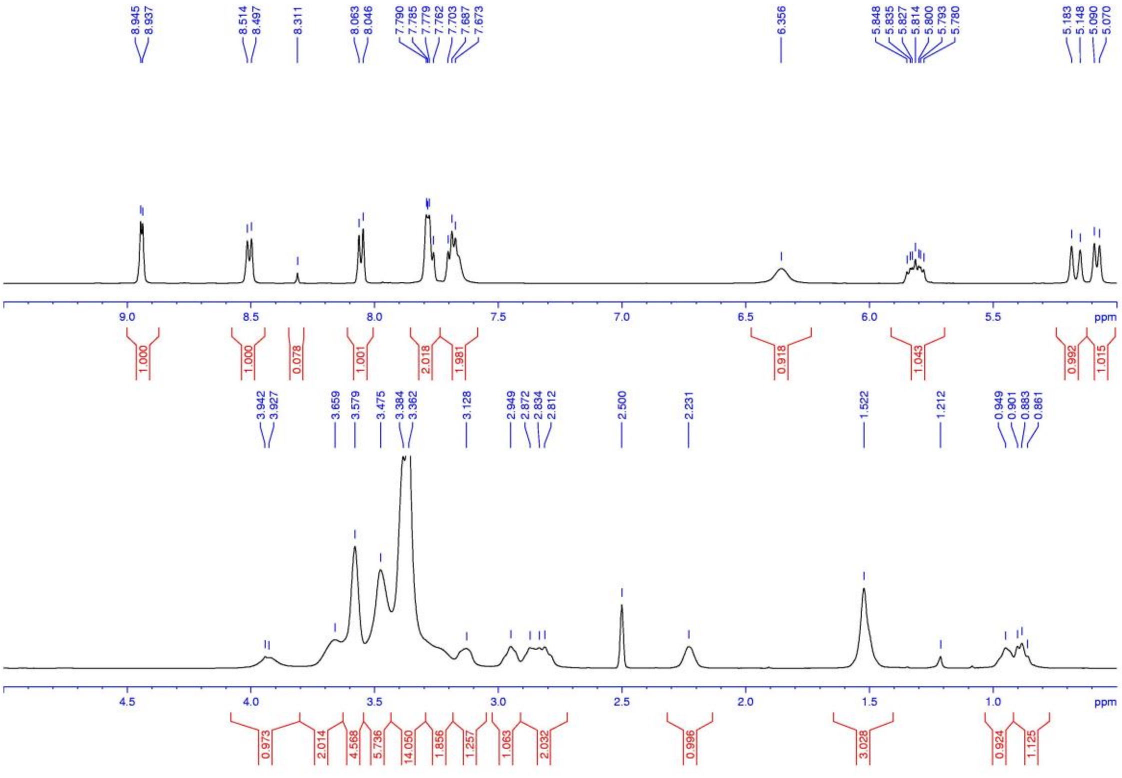

Figure S10. ^{1}H NMR spectrum of catalyst **C5** (500.13 MHz, DMSO-d_6)

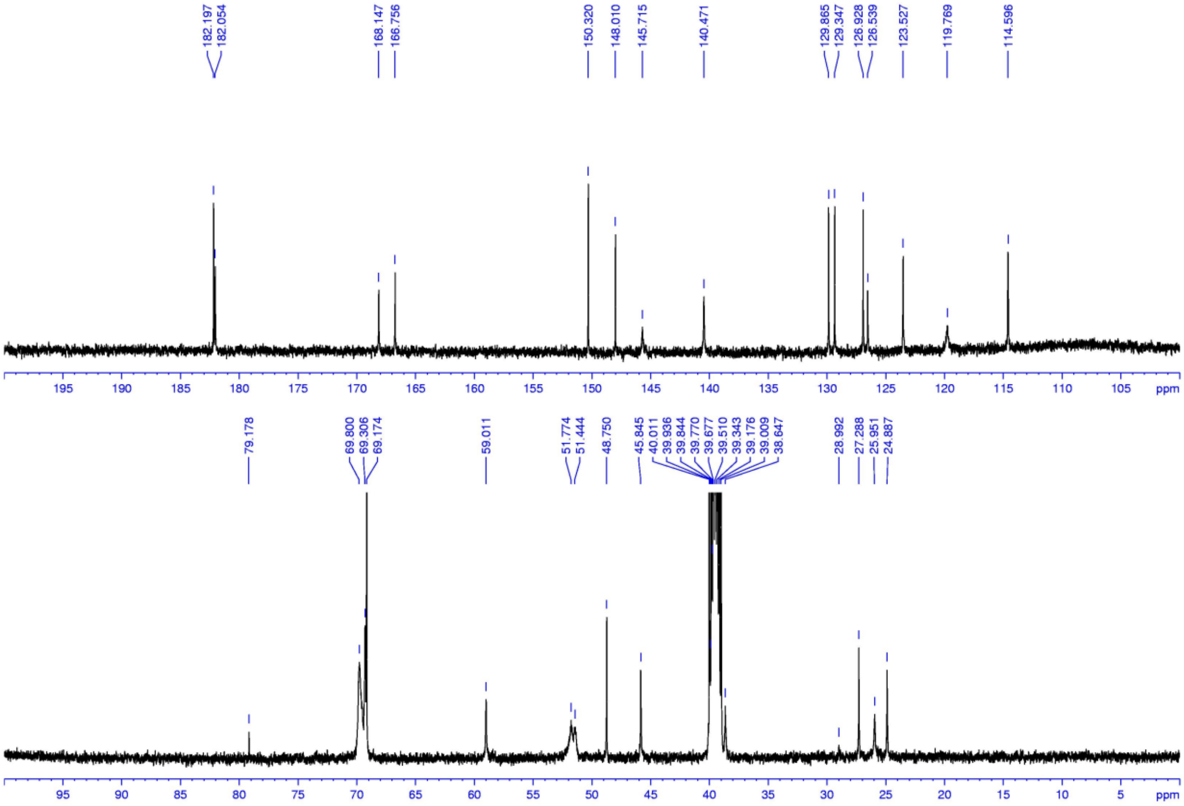

Figure S11. ^{13}C NMR spectrum of catalyst **C5** (125.76 MHz, DMSO-d_6)

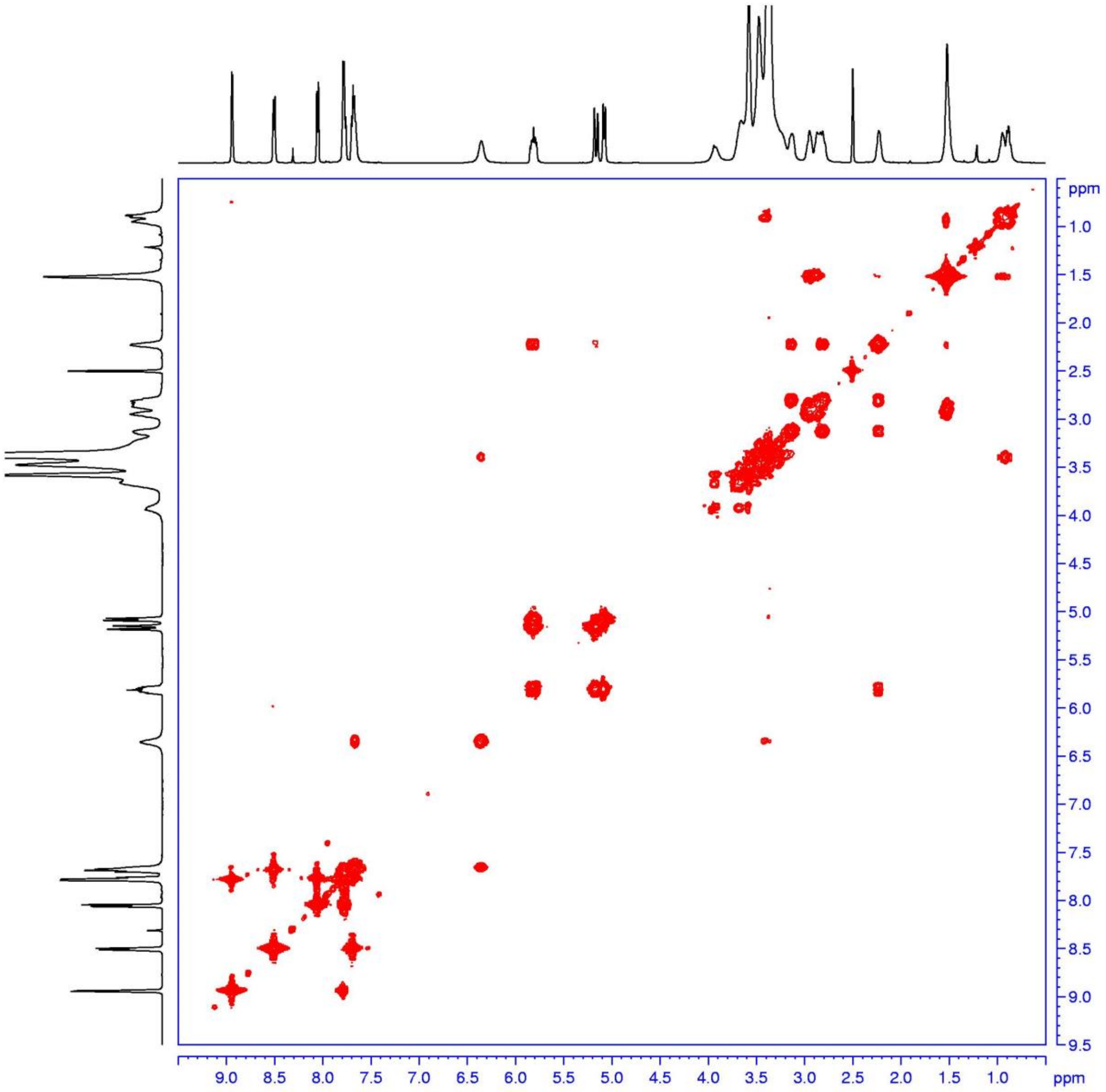

Figure S12. ^{1}H-gs-COSY NMR spectrum of catalyst **C5** (DMSO-d_6)

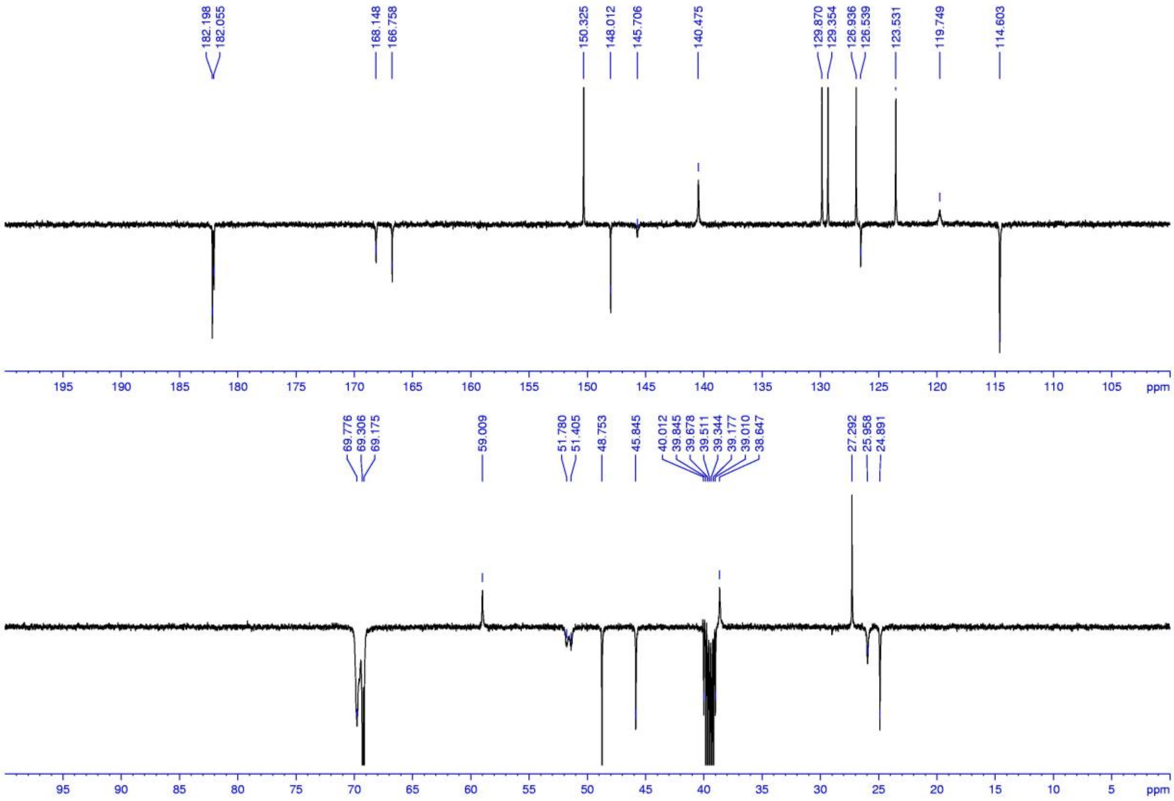

Figure S13. ^{13}C DEPTQ NMR spectrum of catalyst **C5** (DMSO-d_6)

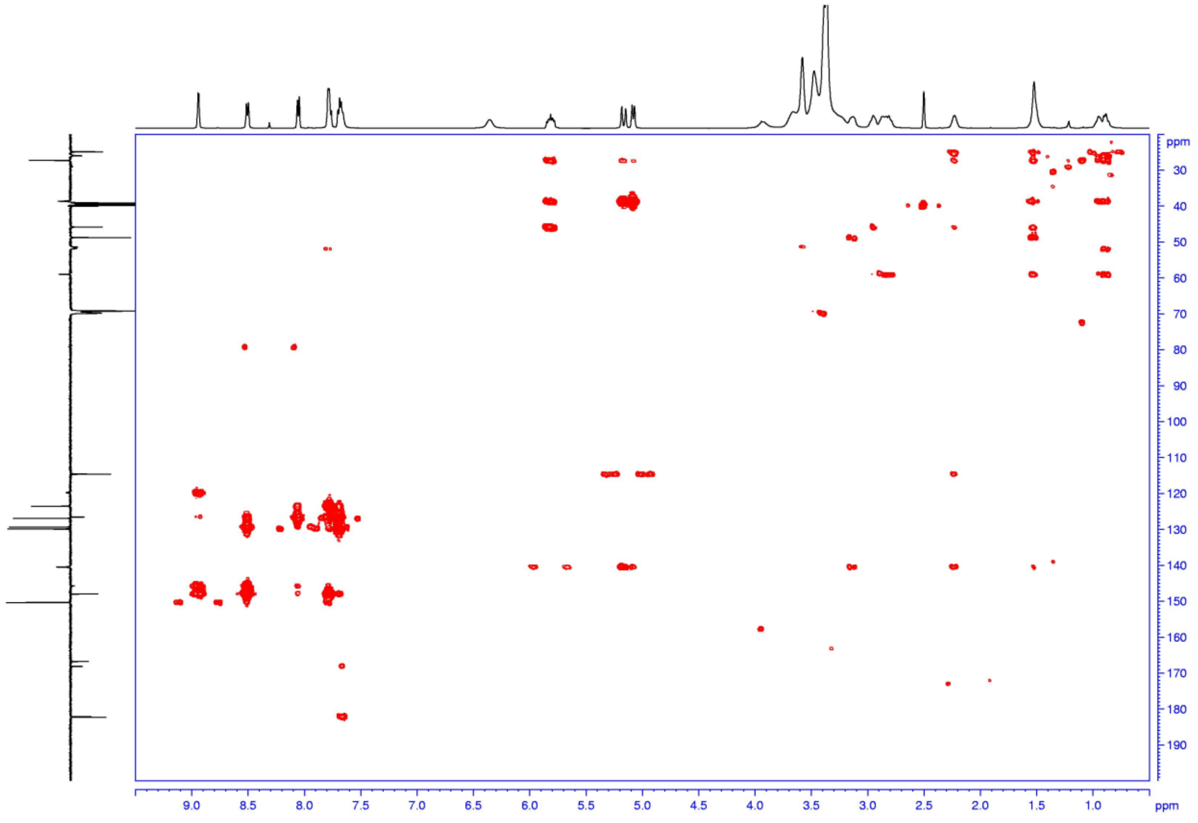

Figure S14. ^{1}H,^{13}C-gs-HMBC NMR spectrum of catalyst **C5** (DMSO-d_6)

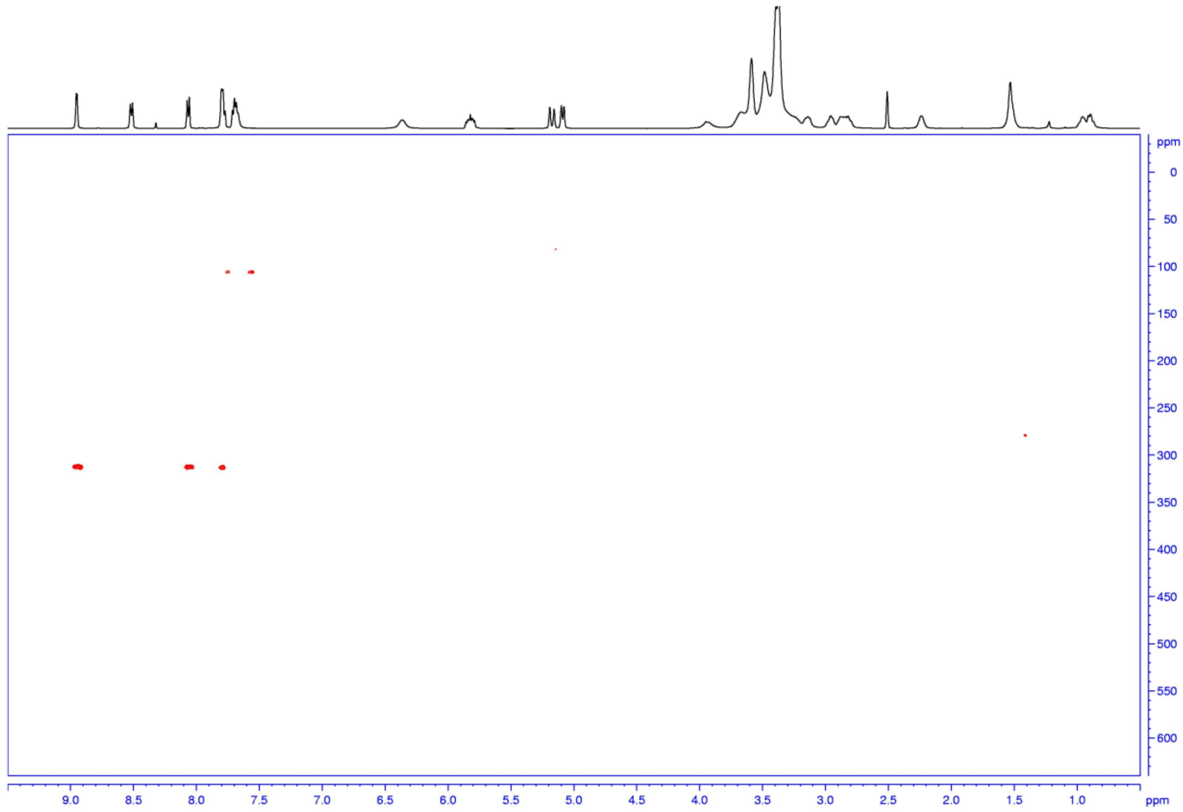

Figure S15. $^{1}H,^{15}N$-gs-HMBC NMR spectrum of catalyst **C5** (DMSO-d$_6$)

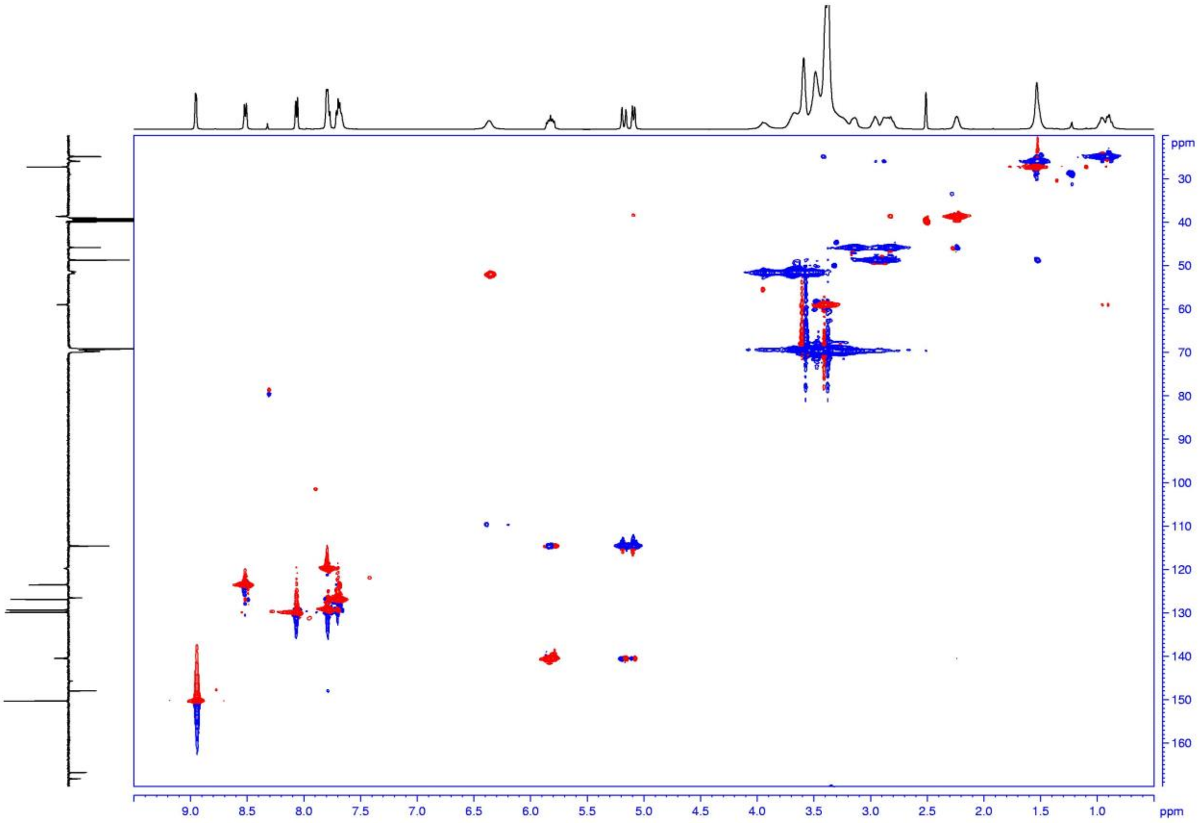

Figure S16. $^{1}H,^{13}C$-gs-HSQC NMR spectrum of intermediate **C5** (DMSO-d$_6$)

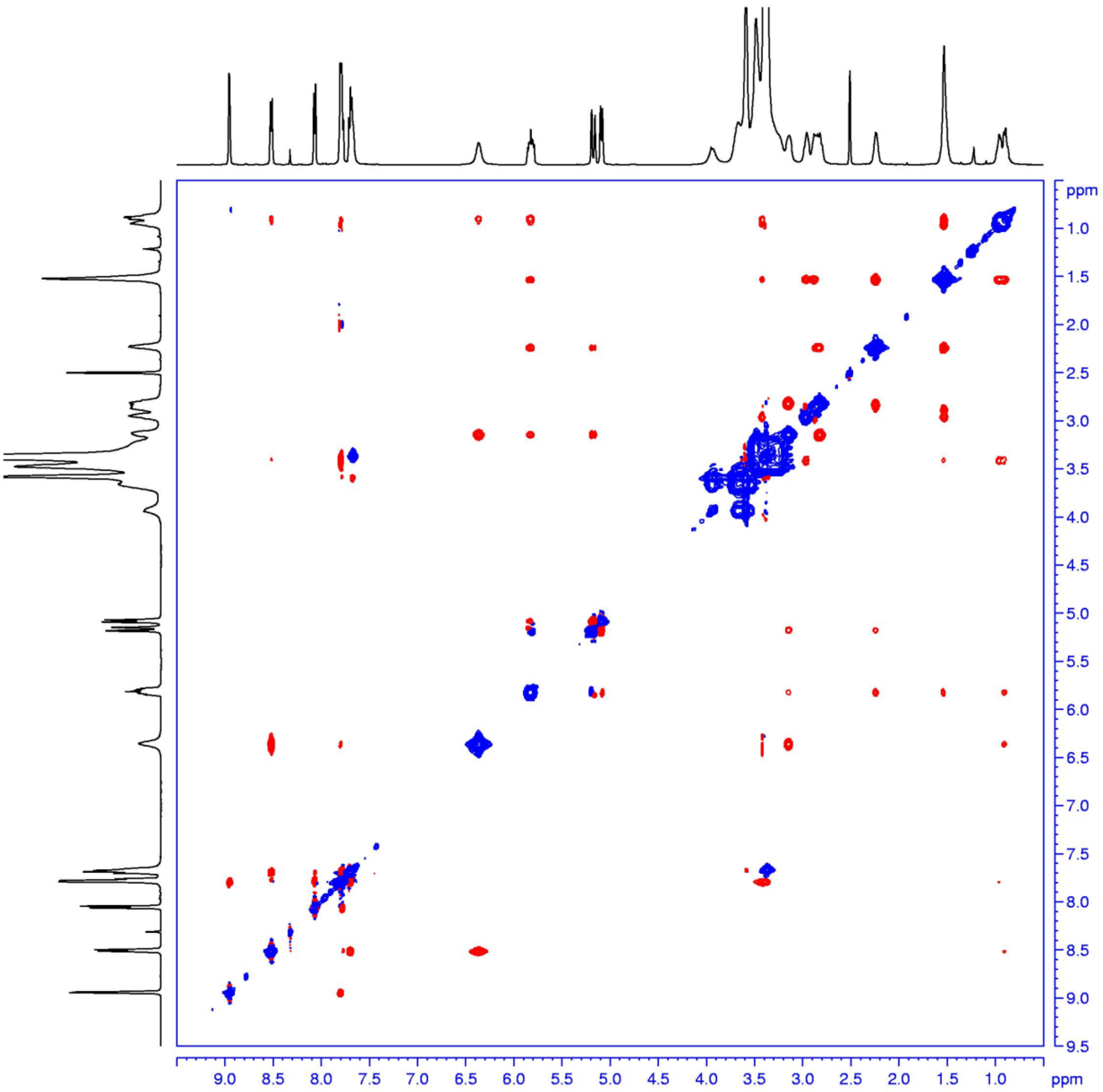

Figure S17. ^{1}H,^{1}H-gs-ROESY NMR spectrum of intermediate **C5** (DMSO-d_6)

NMR spectra of catalyst **C6**

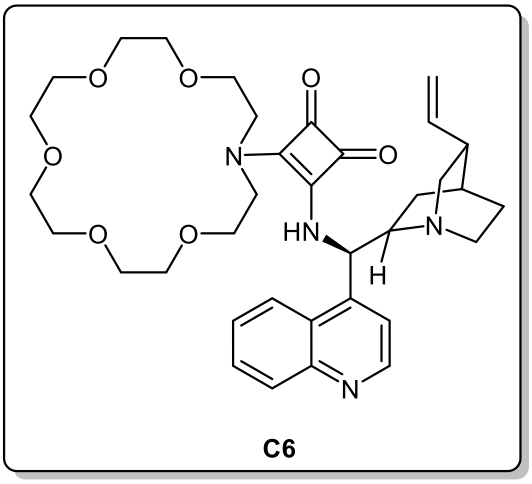

Figure S18. Molecule structure of catalyst **C6**

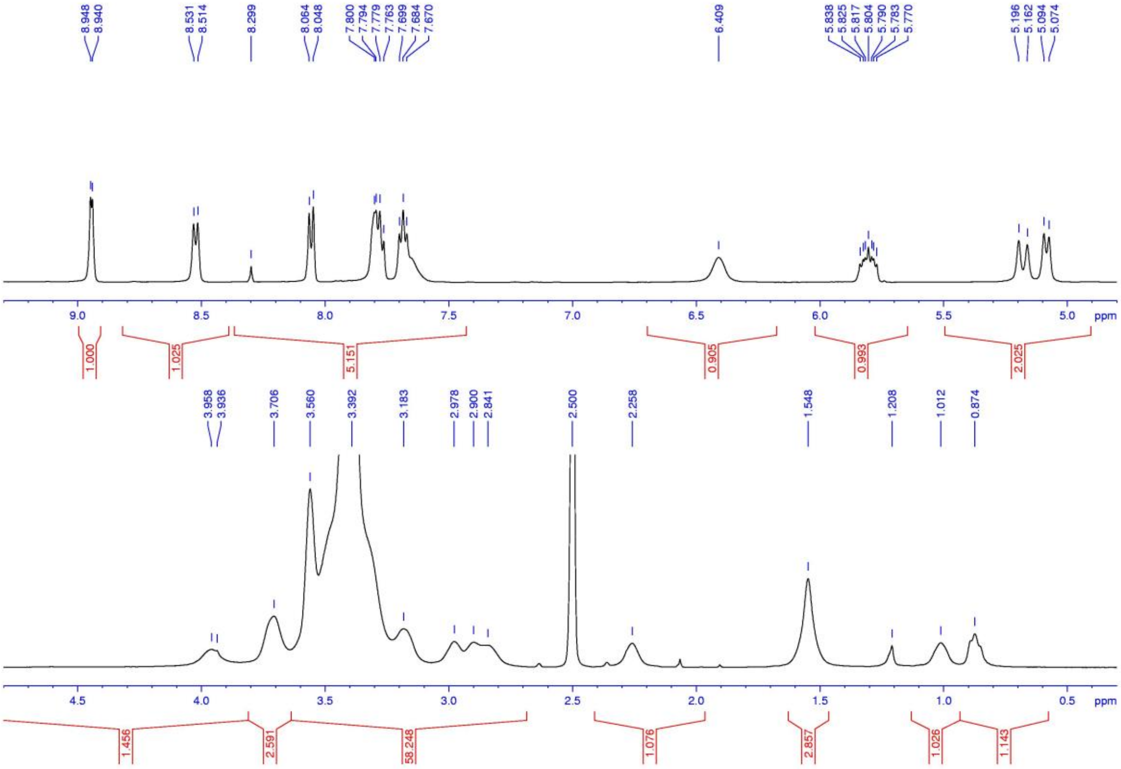

Figure S19. ^{1}H NMR spectrum of catalyst **C6** (500.13 MHz, DMSO-d_6)

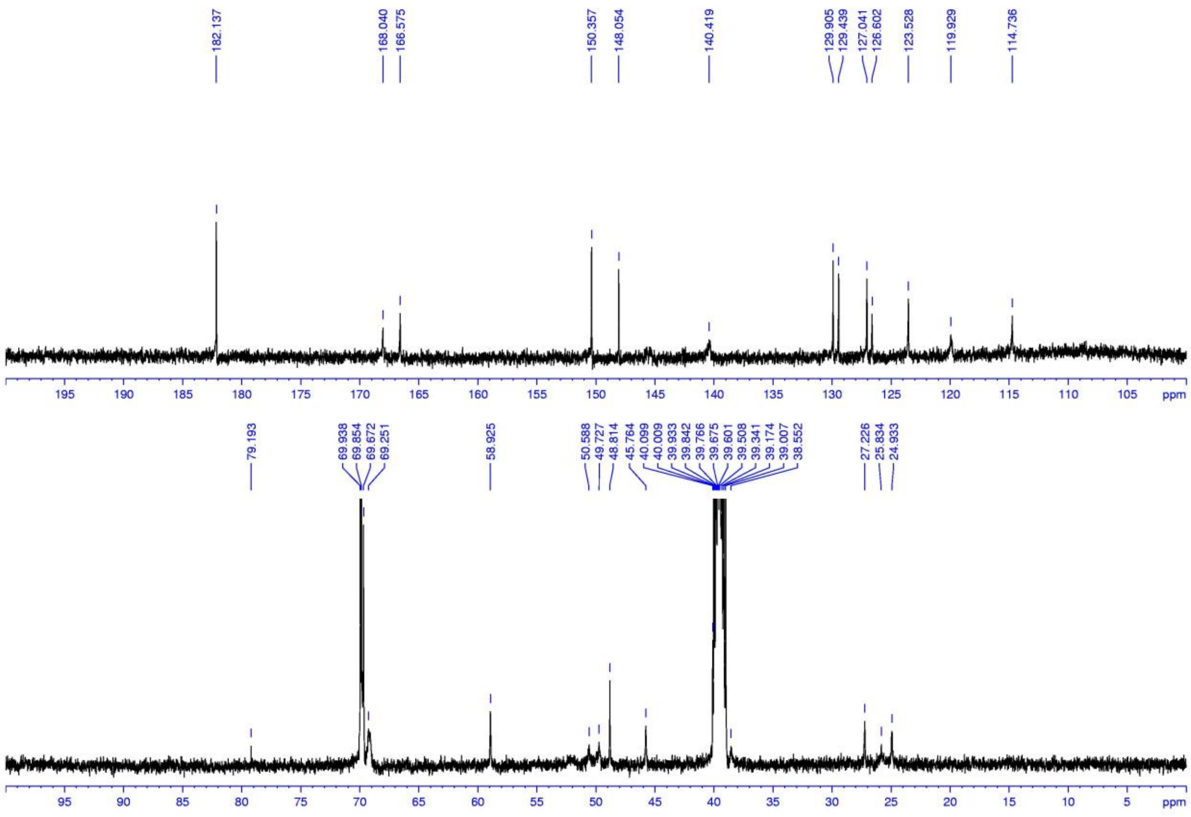

Figure S20. ^{13}C NMR spectrum of catalyst **C6** (125.76 MHz, DMSO-d_6)

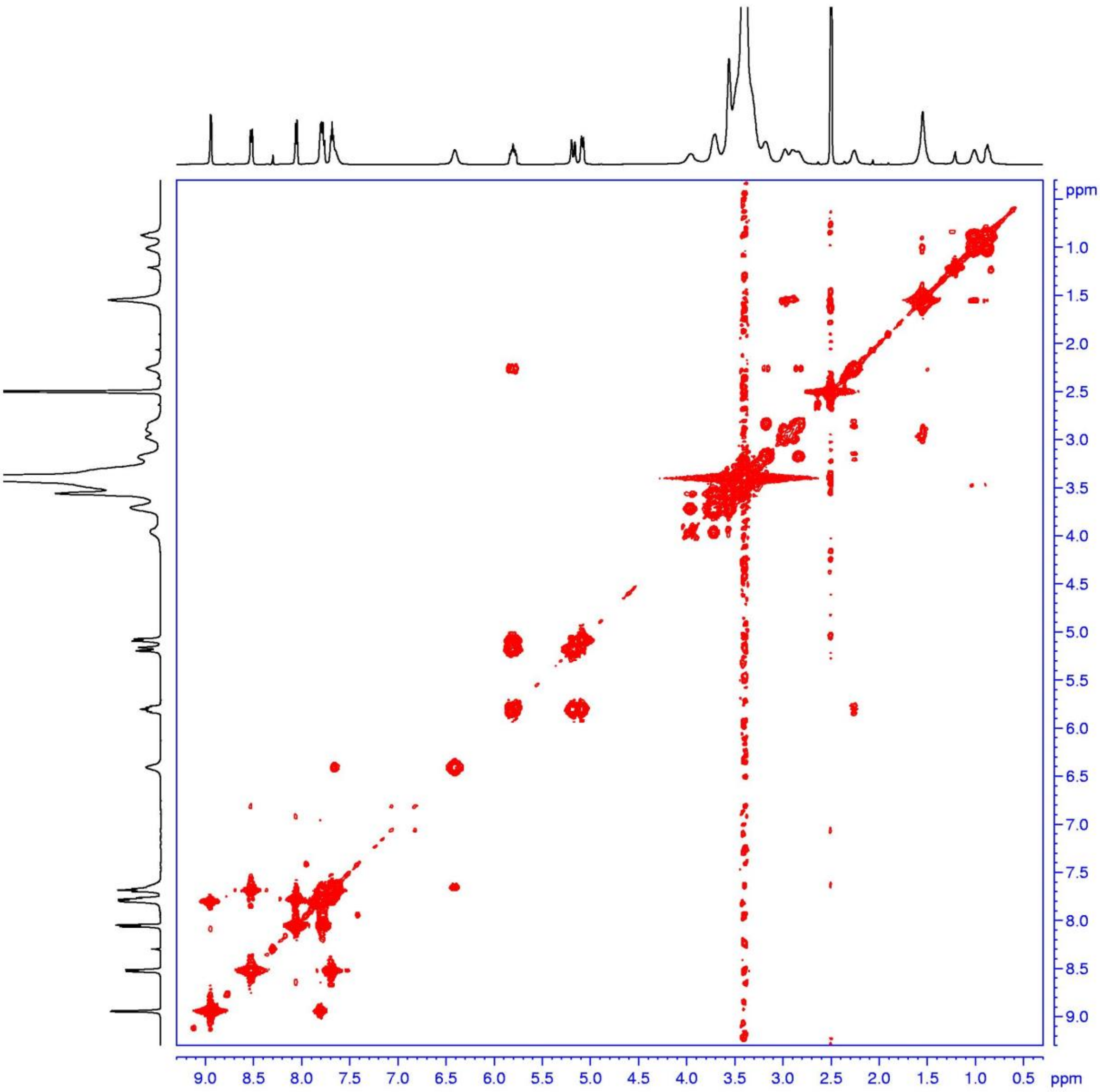

Figure S21. ^{1}H-gs-COSY NMR spectrum of catalyst **C6** (DMSO-d_6)

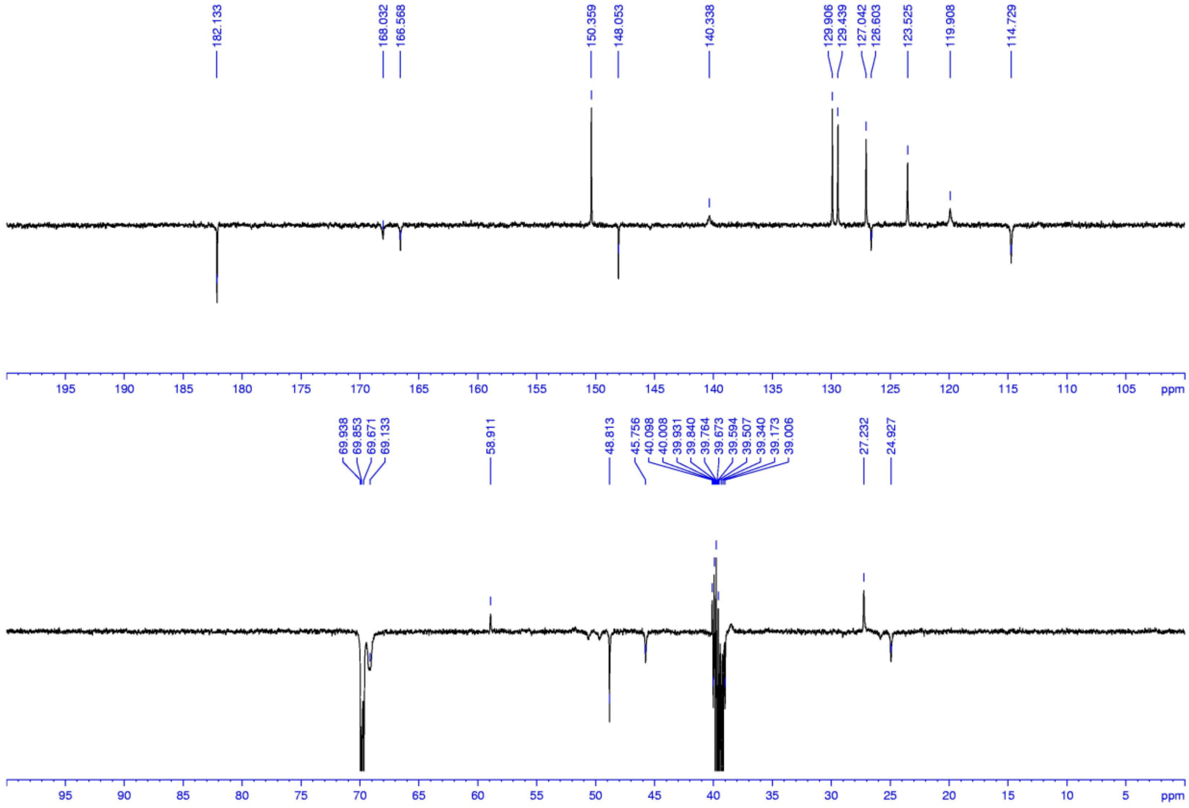

Figure S22. ^{13}C DEPTQ NMR spectrum of catalyst **C6** ($DMSO-d_6$)

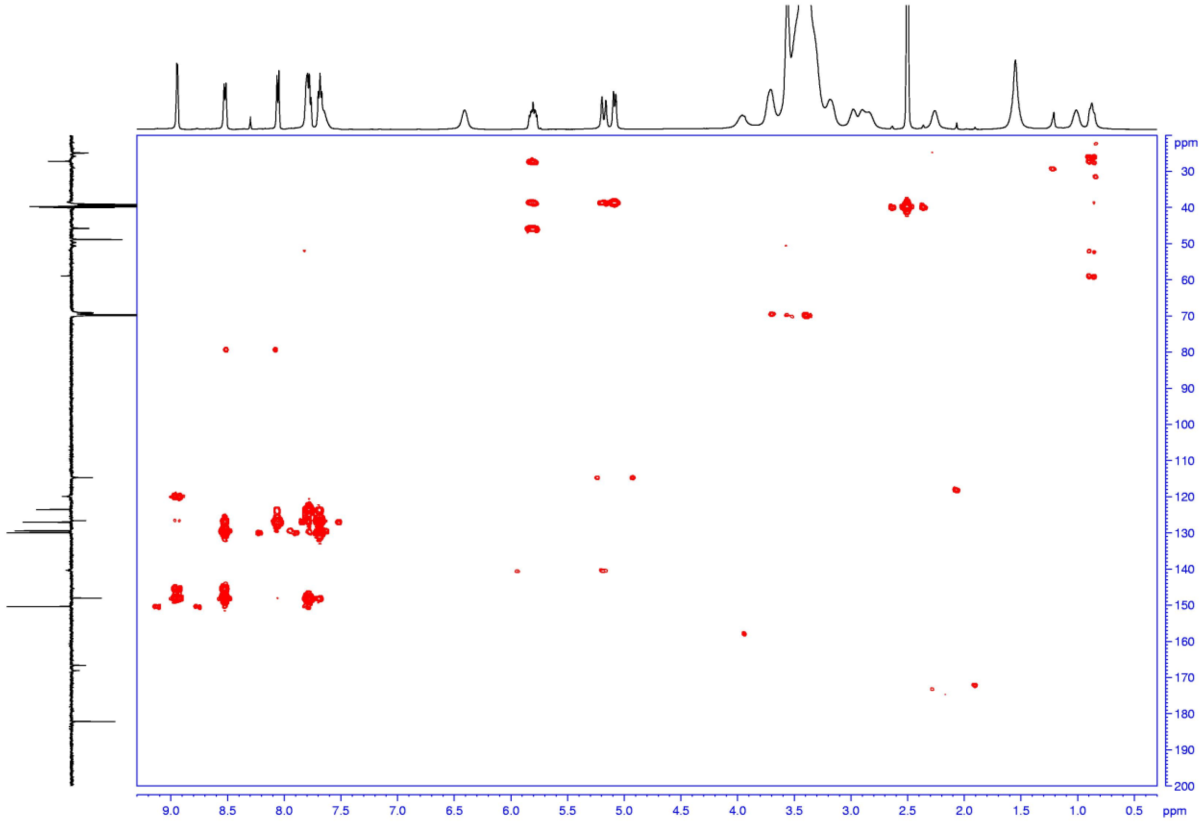

Figure S23. ^{1}H,^{13}C-gs-HMBC NMR spectrum of catalyst **C6** ($DMSO-d_6$)

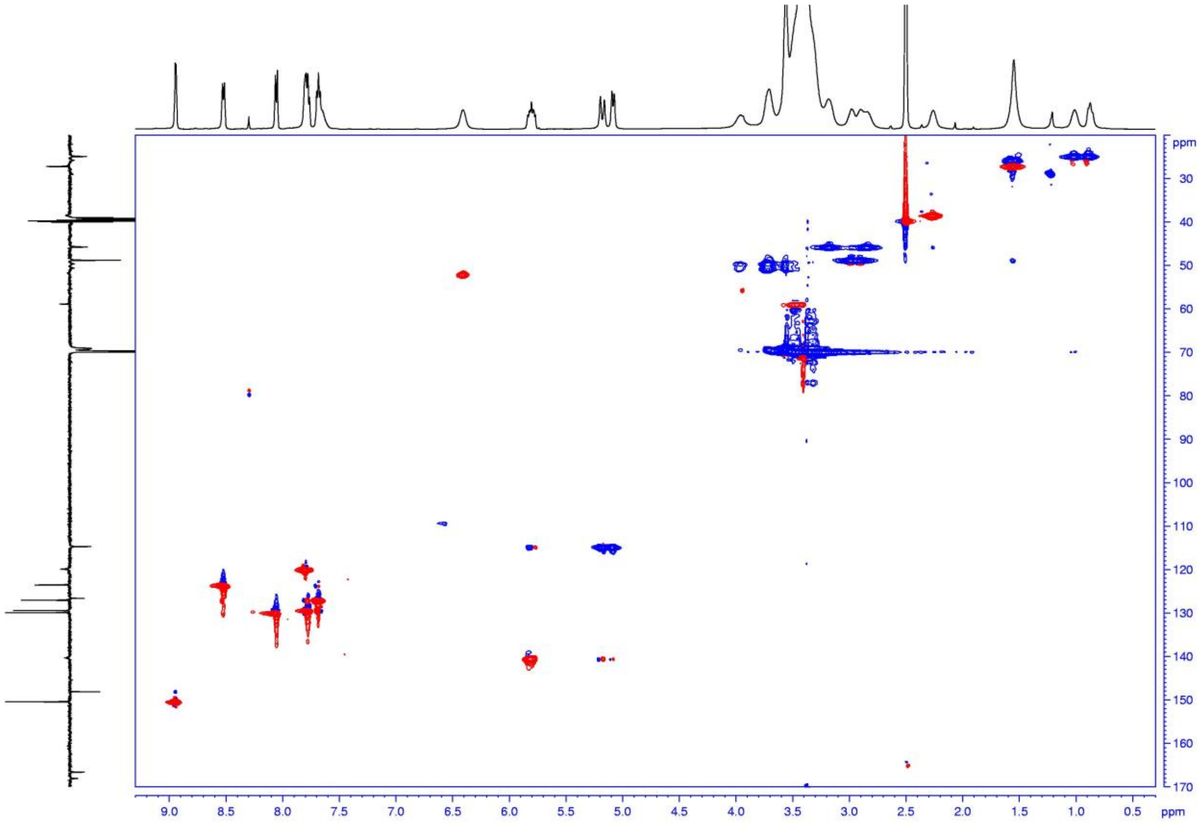

Figure S24. ^{1}H,^{13}C-gs-HSQC NMR spectrum of intermediate **C6** (DMSO-d_6)

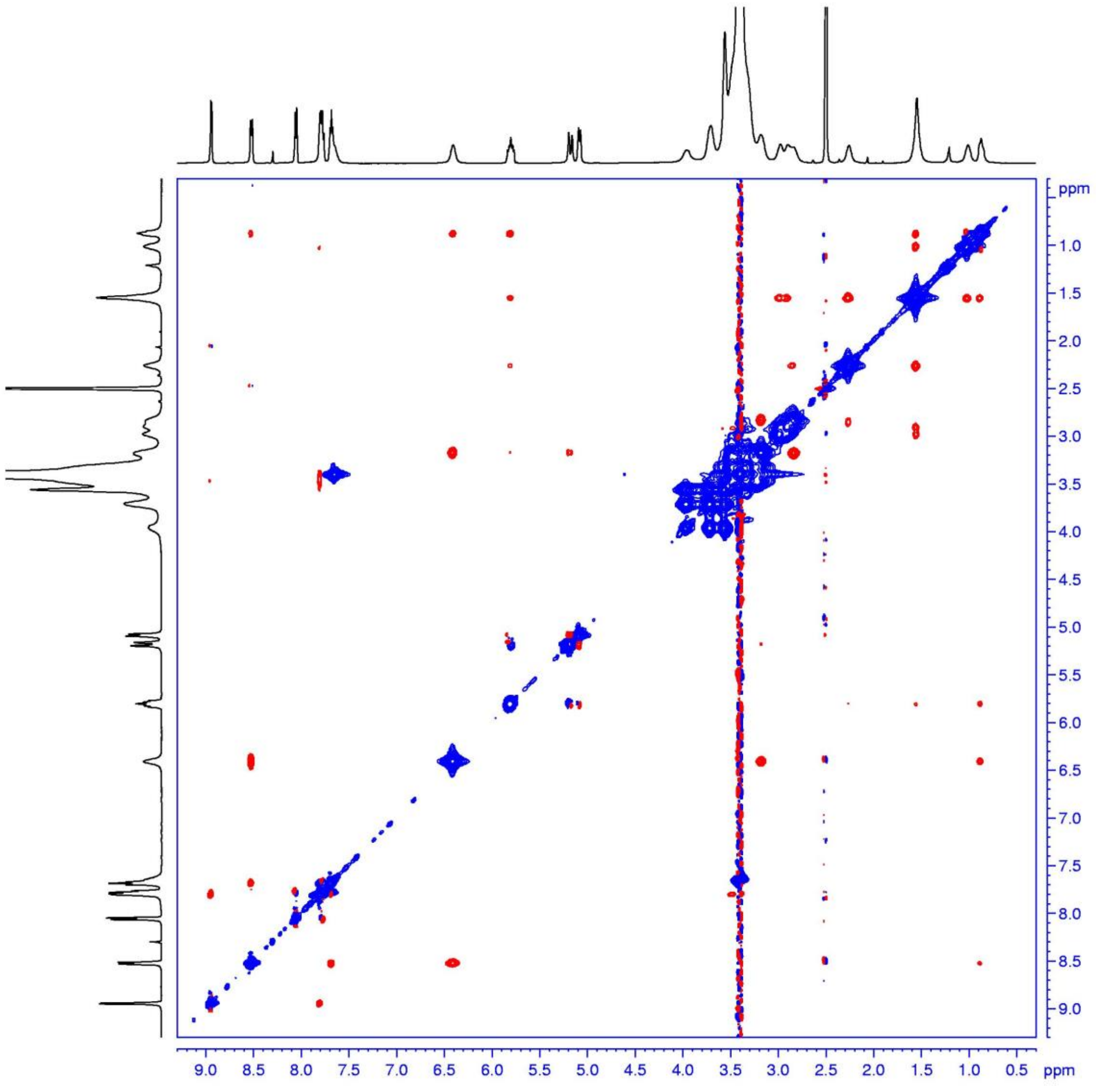

Figure S25. ^{1}H,^{1}H-gs-ROESY NMR spectrum of intermediate **C6** (DMSO-d$_6$)

NMR spectra of catalyst **Q5**

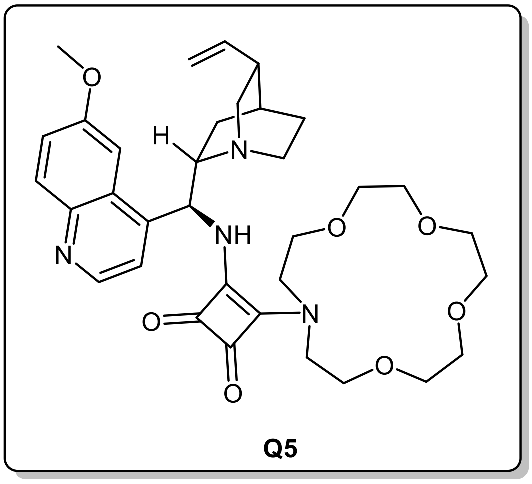

Figure S26. Molecule structure of catalyst **Q5**

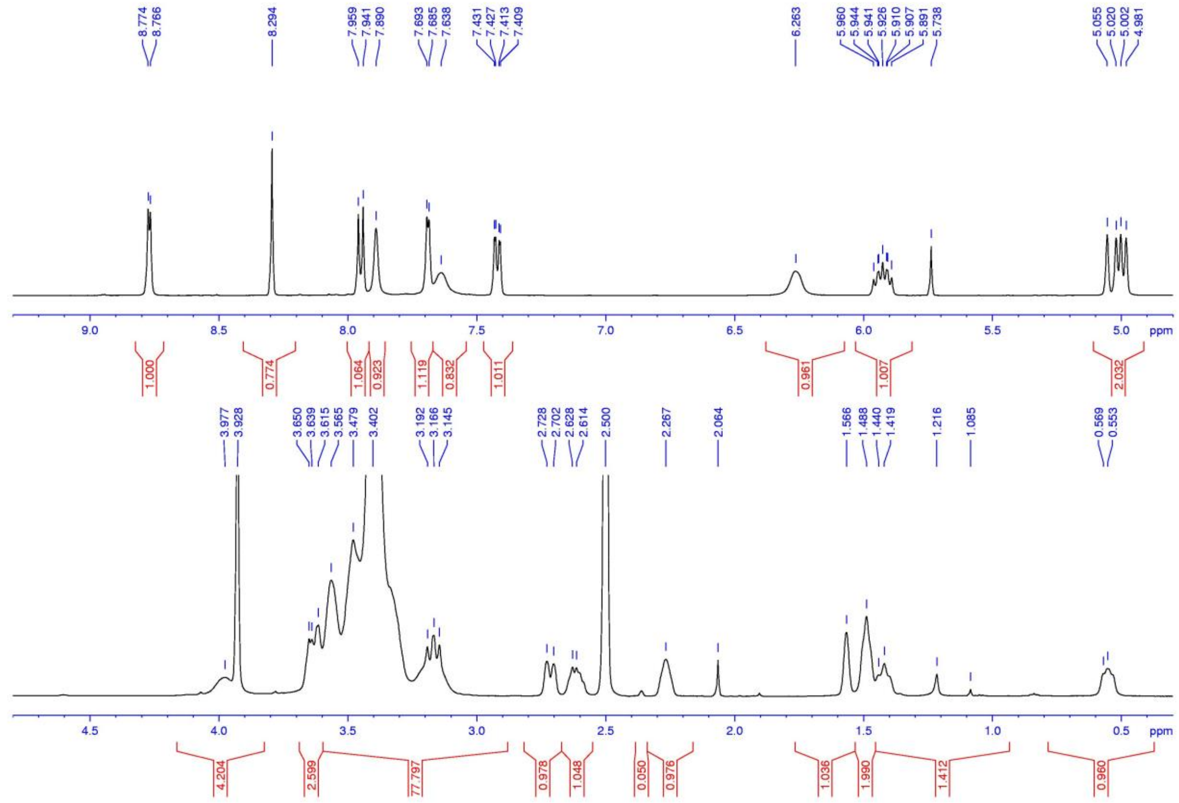

Figure S27. ^{1}H NMR spectrum of catalyst **Q5** (500.13 MHz, DMSO-d_6)

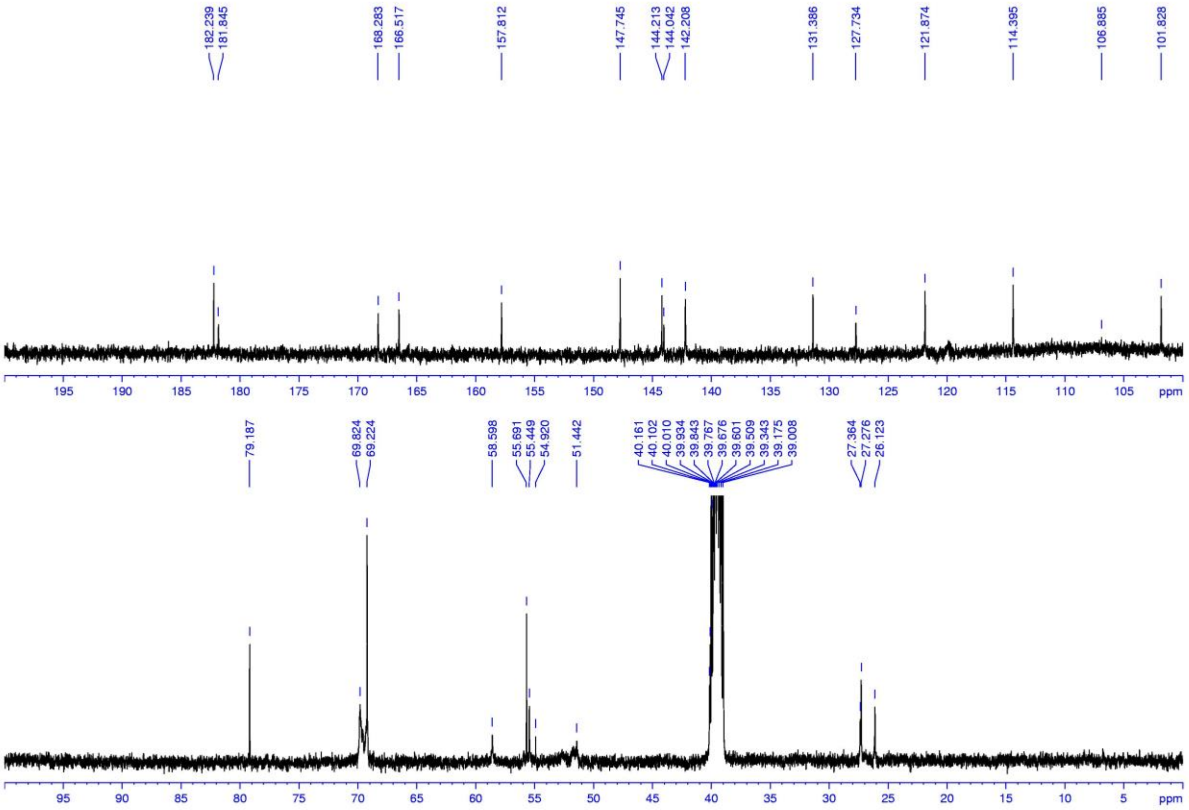

Figure S28. ^{13}C NMR spectrum of catalyst **Q5** (125.76 MHz, DMSO-d_6)

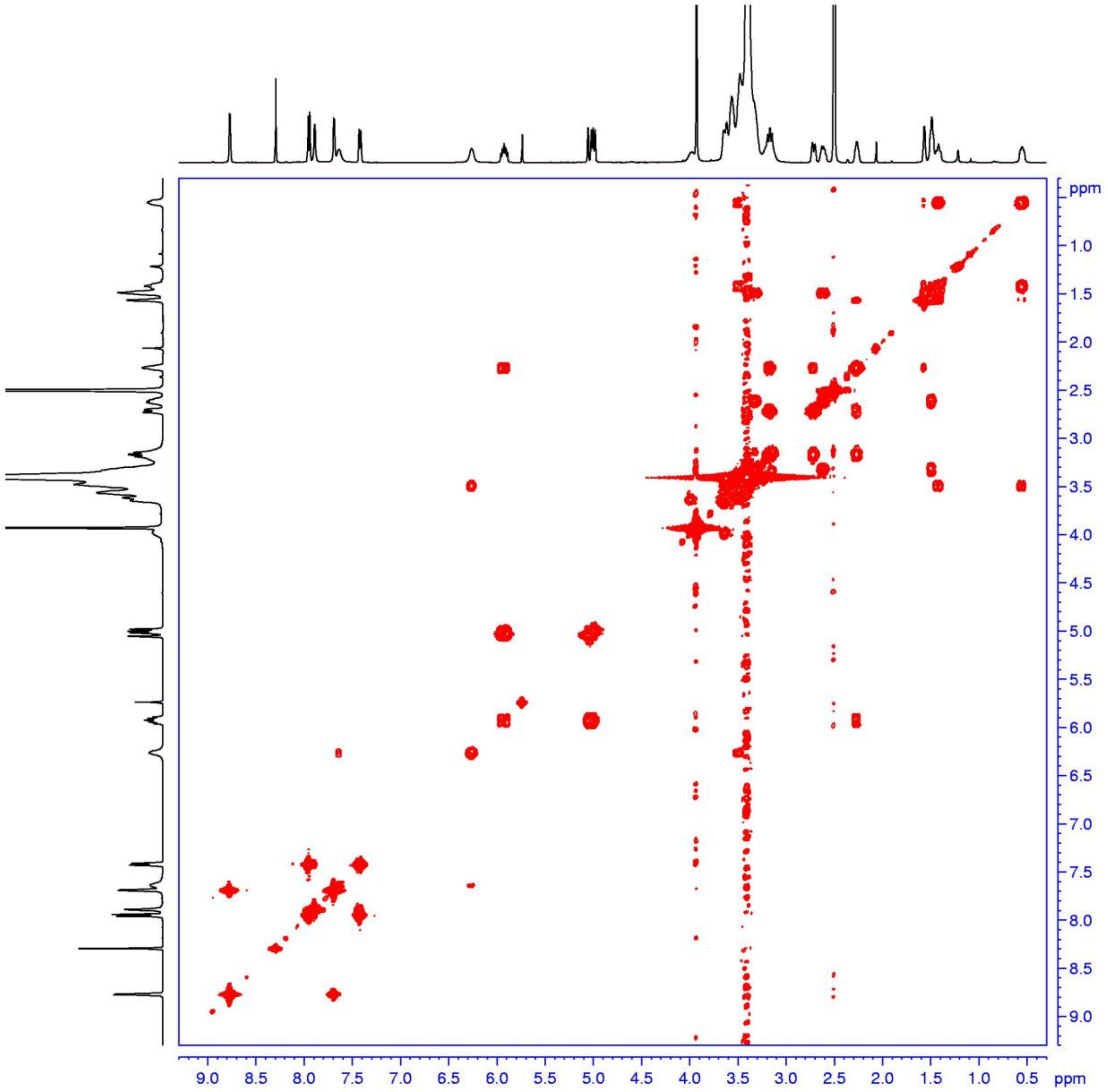

Figure S29. ^{1}H-gs-COSY NMR spectrum of catalyst **Q5** (DMSO-d_6)

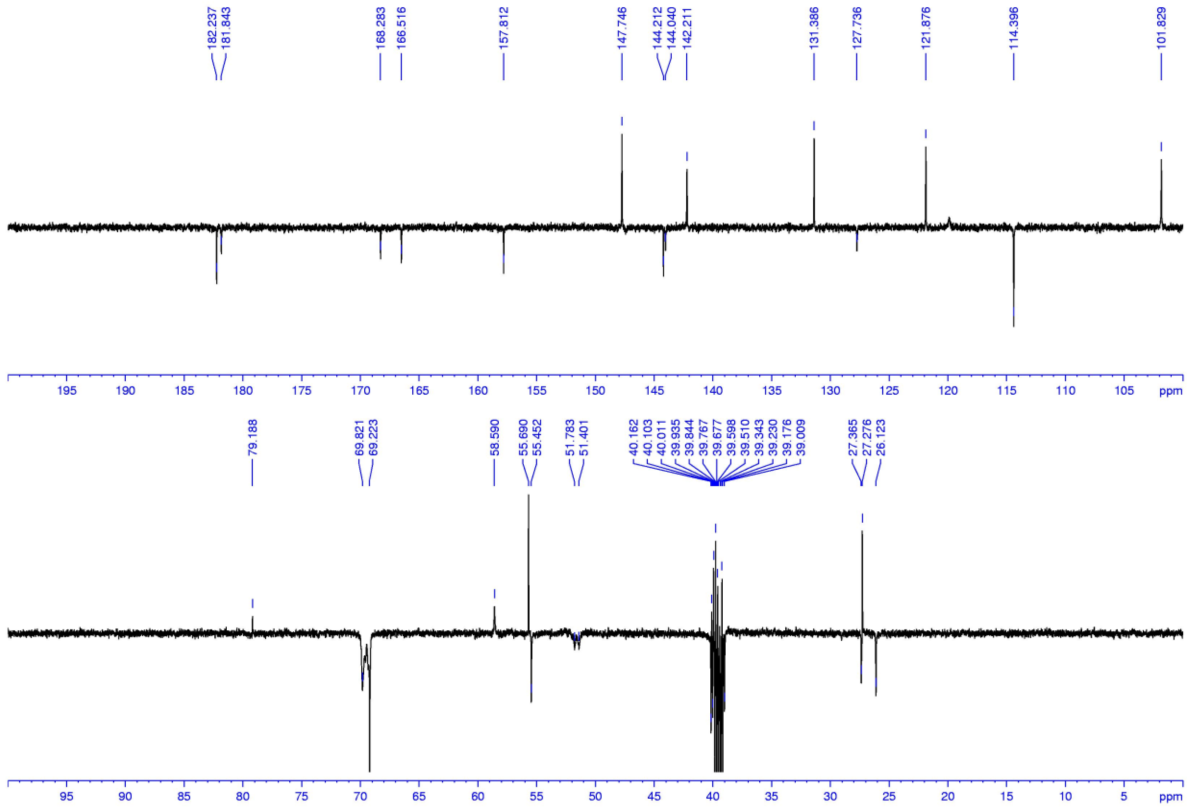

Figure S30. ^{13}C DEPTQ NMR spectrum of catalyst **Q5** (DMSO-d_6)

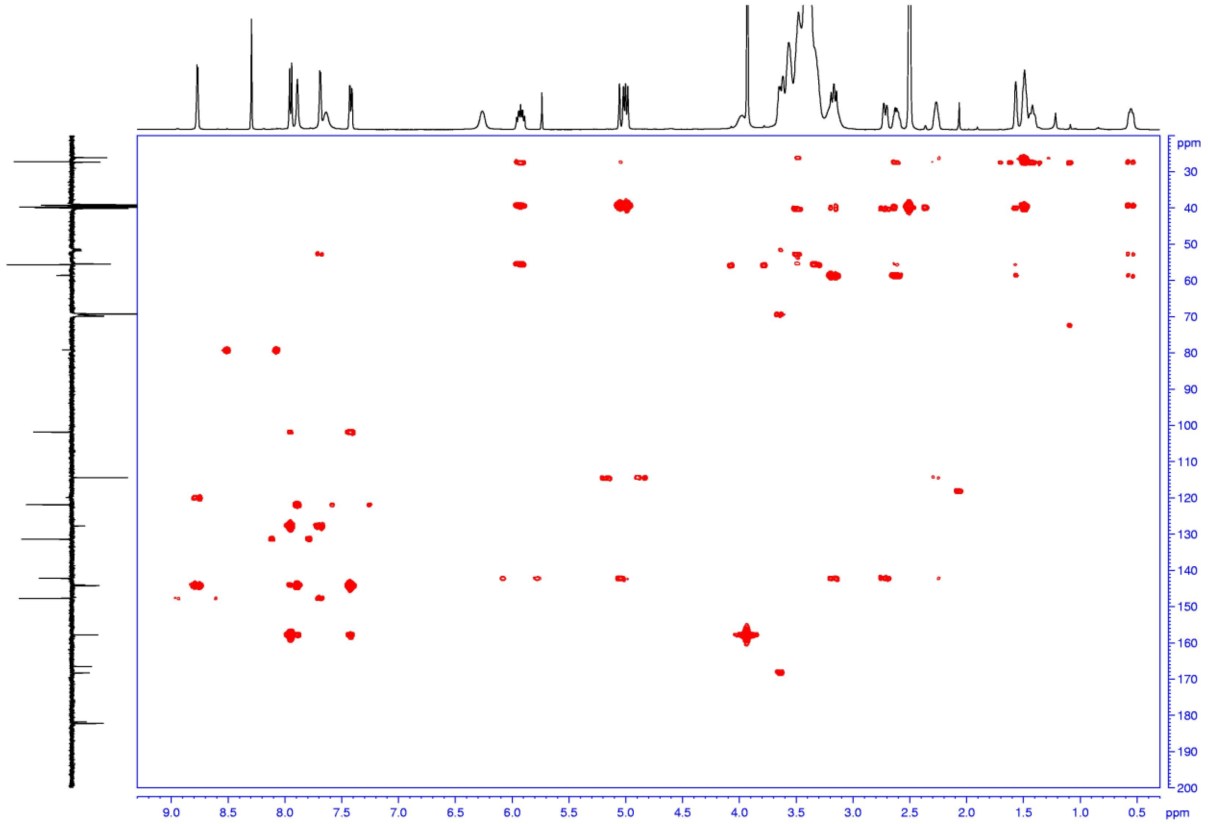

Figure S31. ^{1}H,^{13}C-gs-HMBC NMR spectrum of catalyst **Q5** (DMSO-d_6)

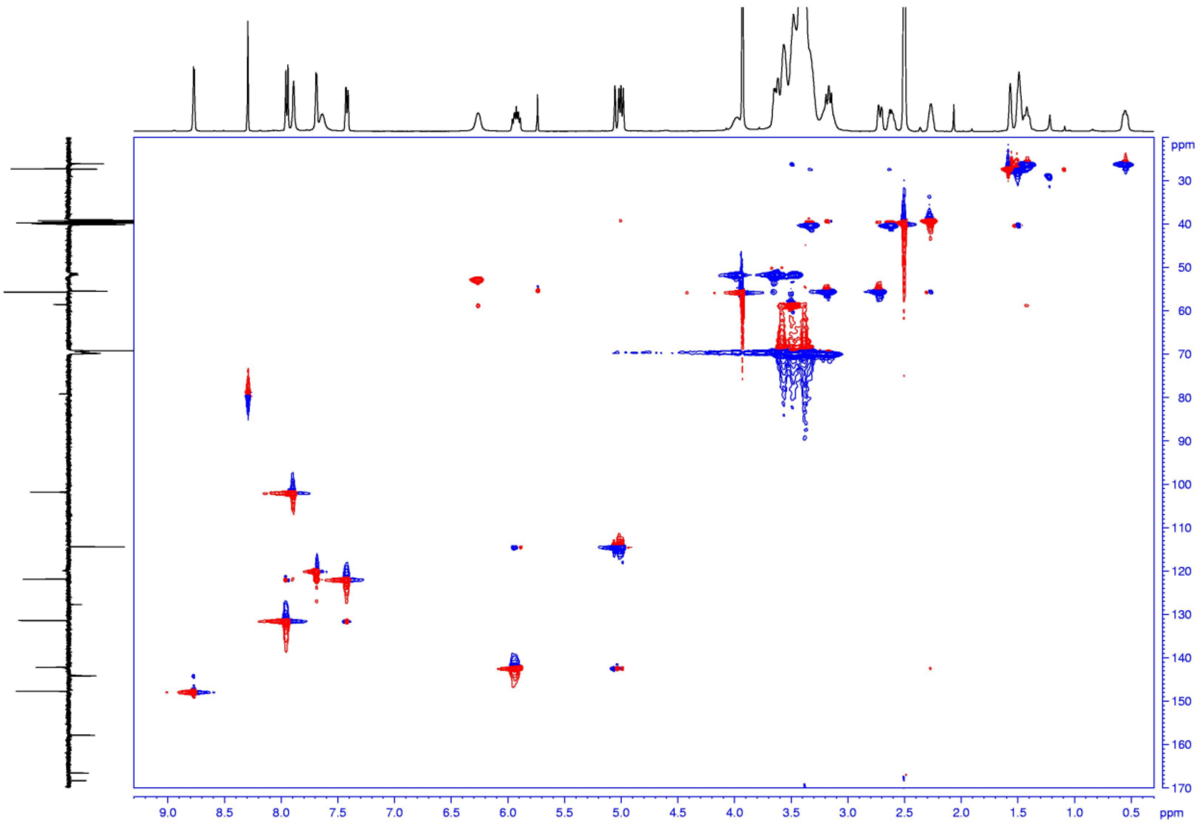

Figure S32. ^{1}H,^{13}C-gs-HSQC NMR spectrum of intermediate **Q5** (DMSO-d_6)

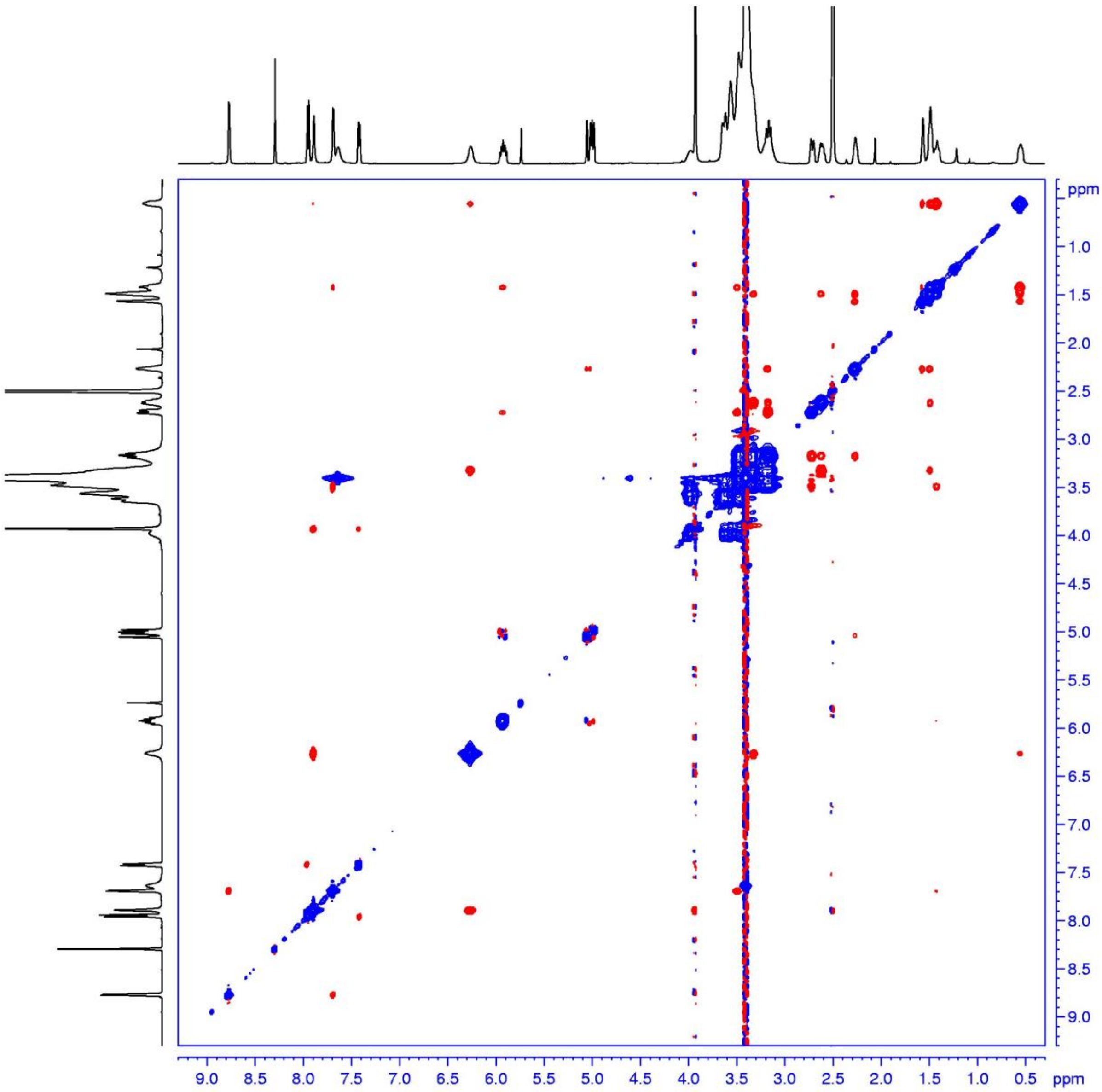

Figure S33. ^{1}H,^{1}H-gs-ROESY NMR spectrum of intermediate **Q5** (DMSO-d_6)

NMR spectra of catalyst **Q6**

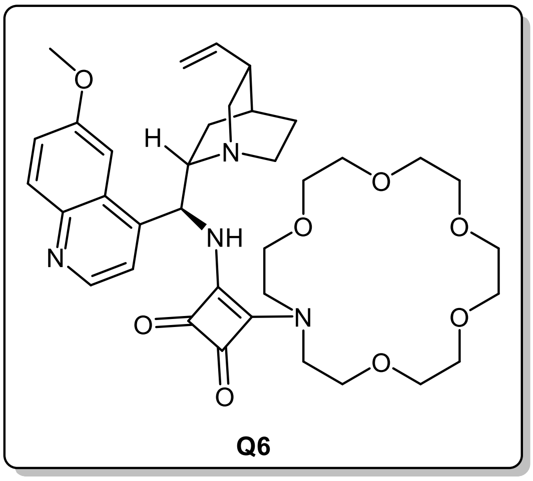

Figure S34. Molecule structure of catalyst **Q6**

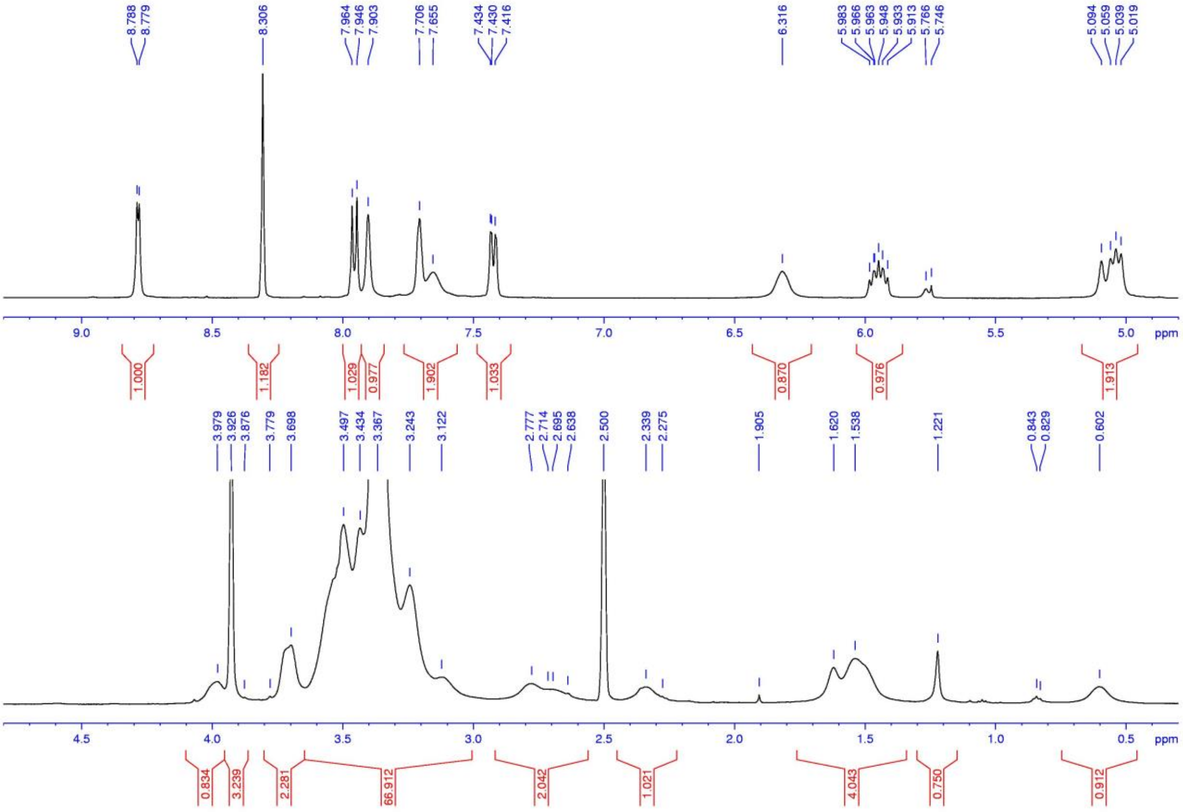

Figure S35. ^{1}H NMR spectrum of catalyst **Q6** (500.13 MHz, DMSO-d_6)

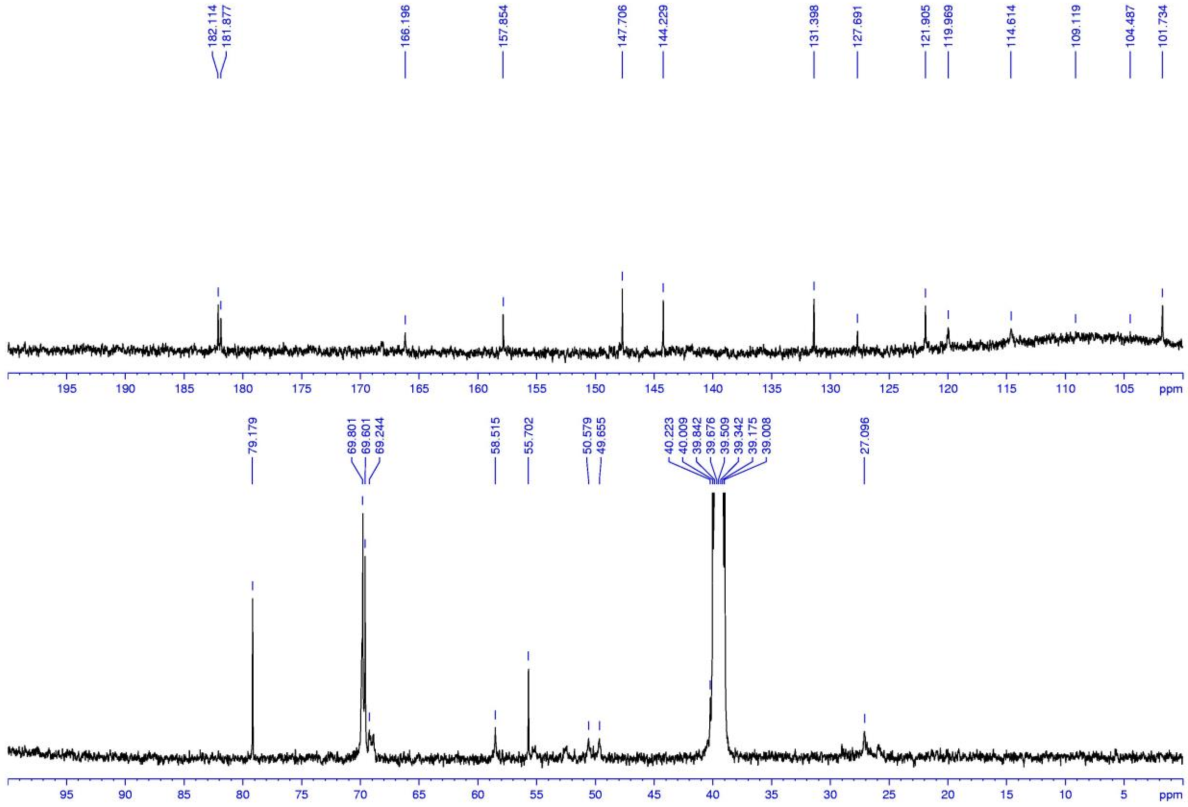

Figure S36. ^{13}C NMR spectrum of catalyst **Q6** (125.76 MHz, DMSO-d_6)

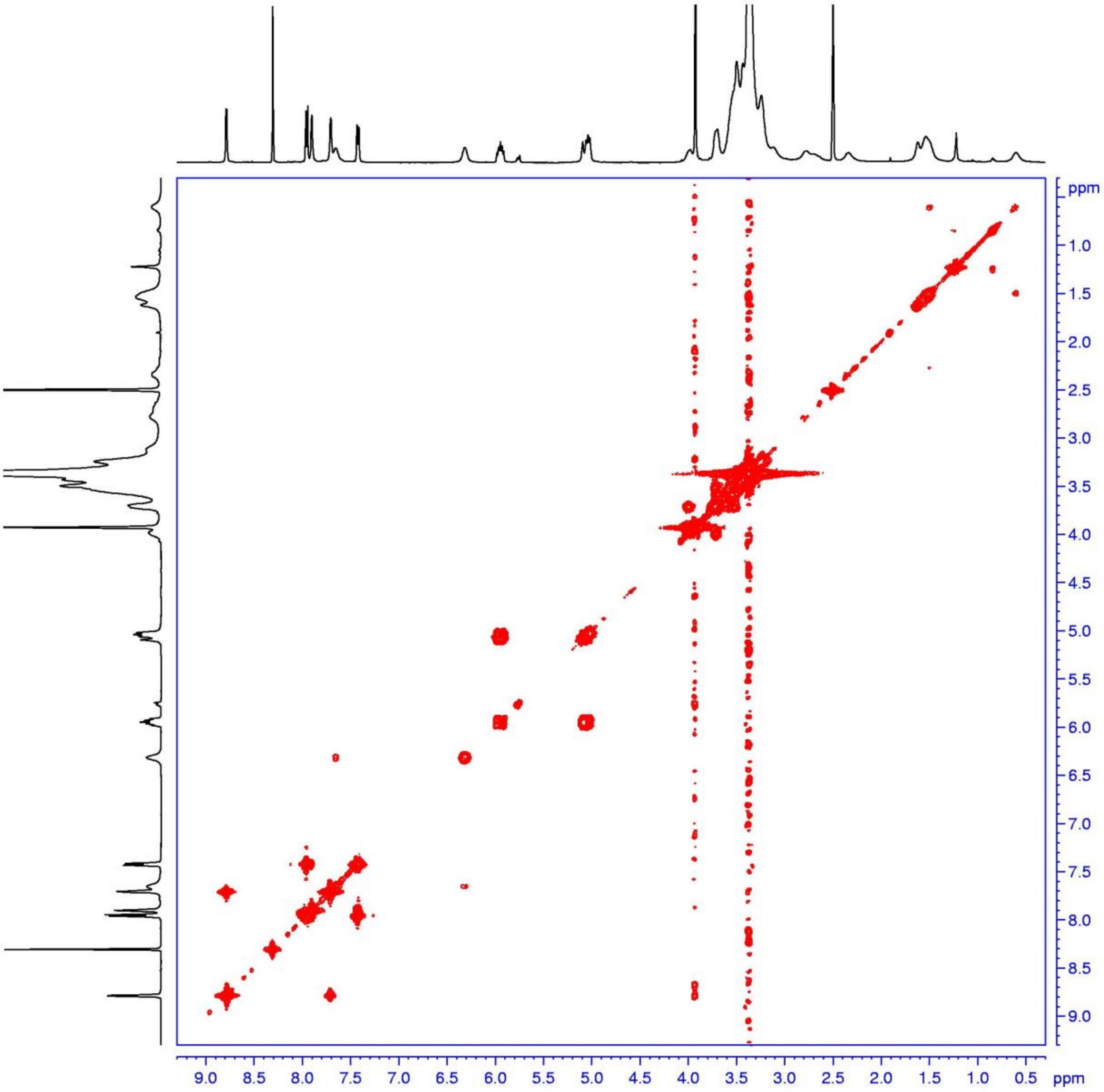

Figure S37. ^{1}H-gs-COSY NMR spectrum of catalyst **Q6** (DMSO-d_6)

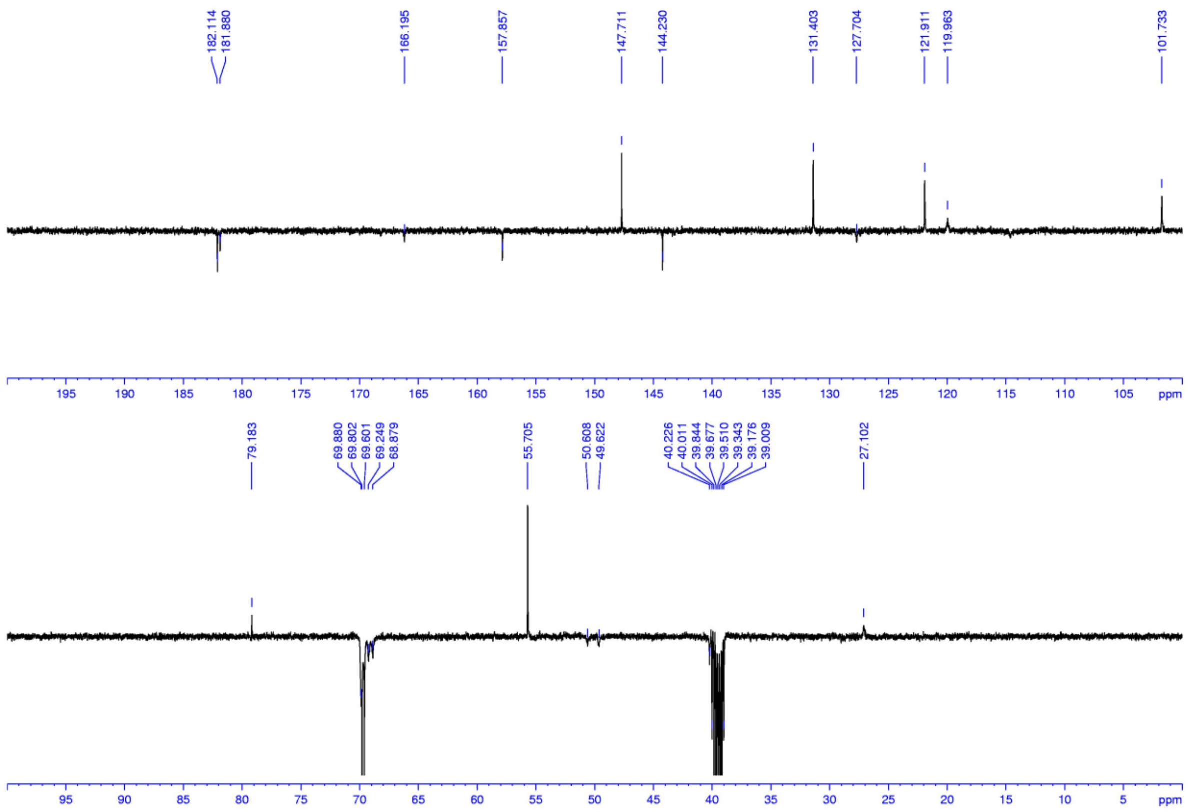

Figure S38. ^{13}C DEPTQ NMR spectrum of catalyst **Q6** (DMSO-d_6)

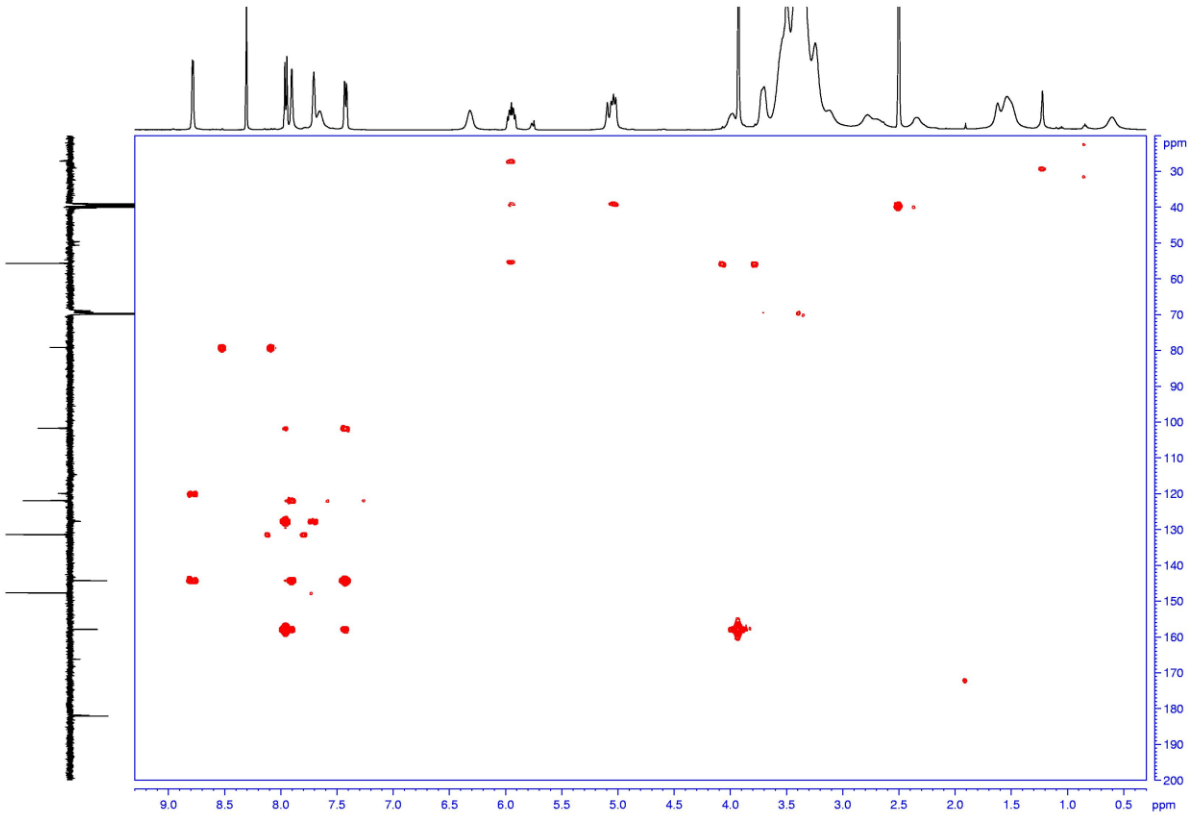

Figure S39. ^{1}H,^{13}C-gs-HMBC NMR spectrum of catalyst **Q6** (DMSO-d_6)

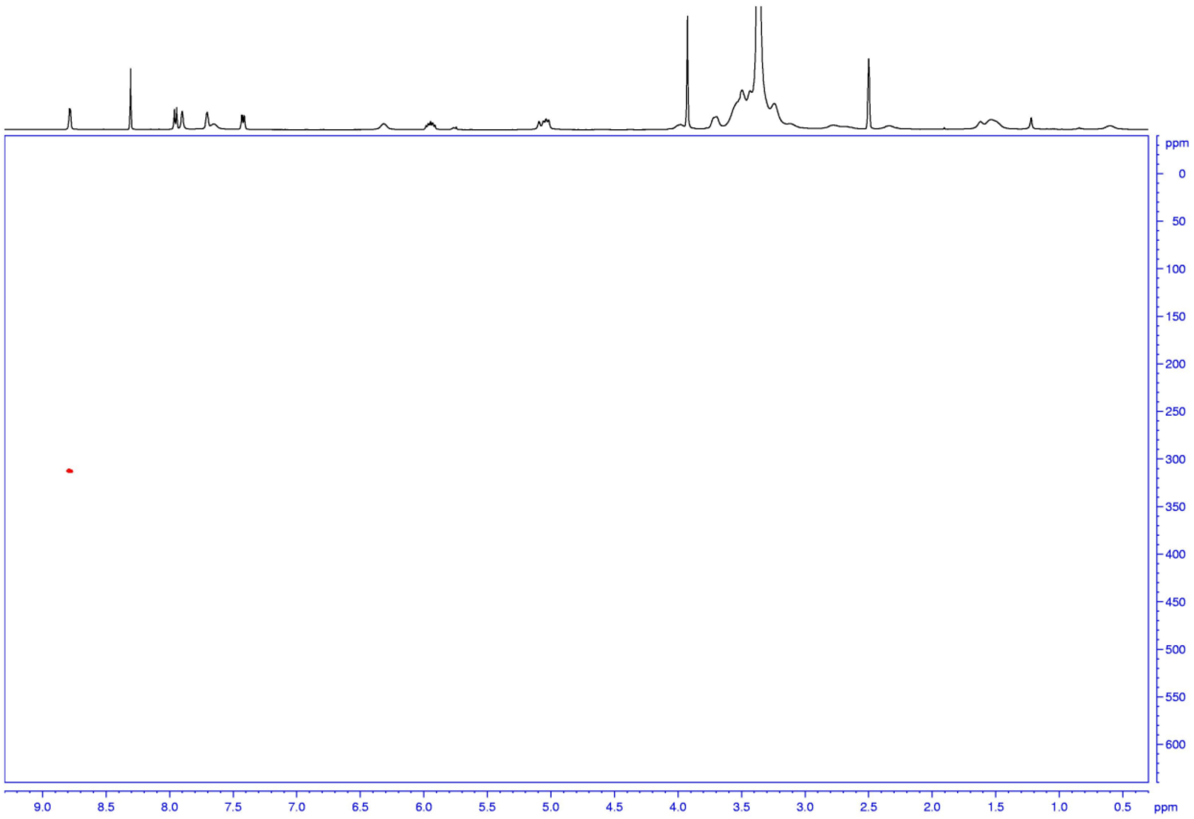

Figure S40. $^{1}H,^{15}N$-gs-HMBC NMR spectrum of catalyst **Q6** (DMSO-d_6)

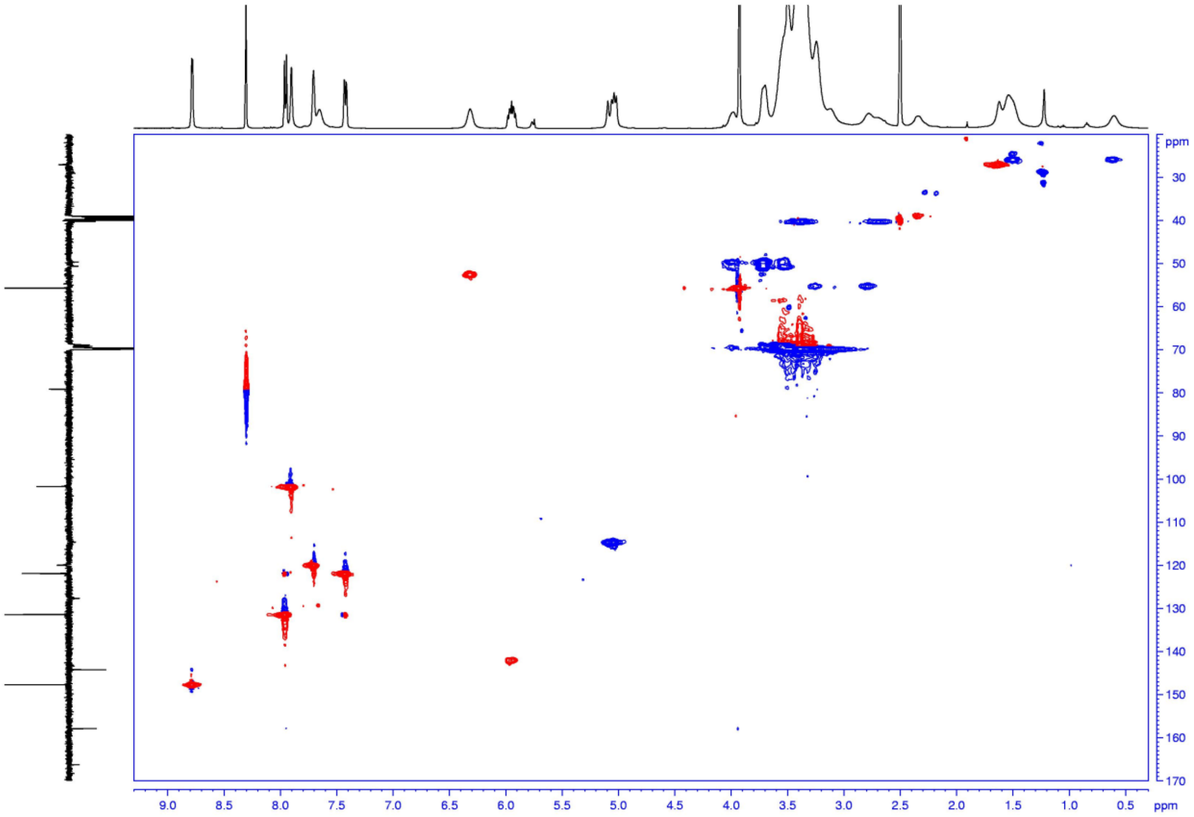

Figure S41. $^{1}H,^{13}C$-gs-HSQC NMR spectrum of intermediate **Q6** (DMSO-d_6)

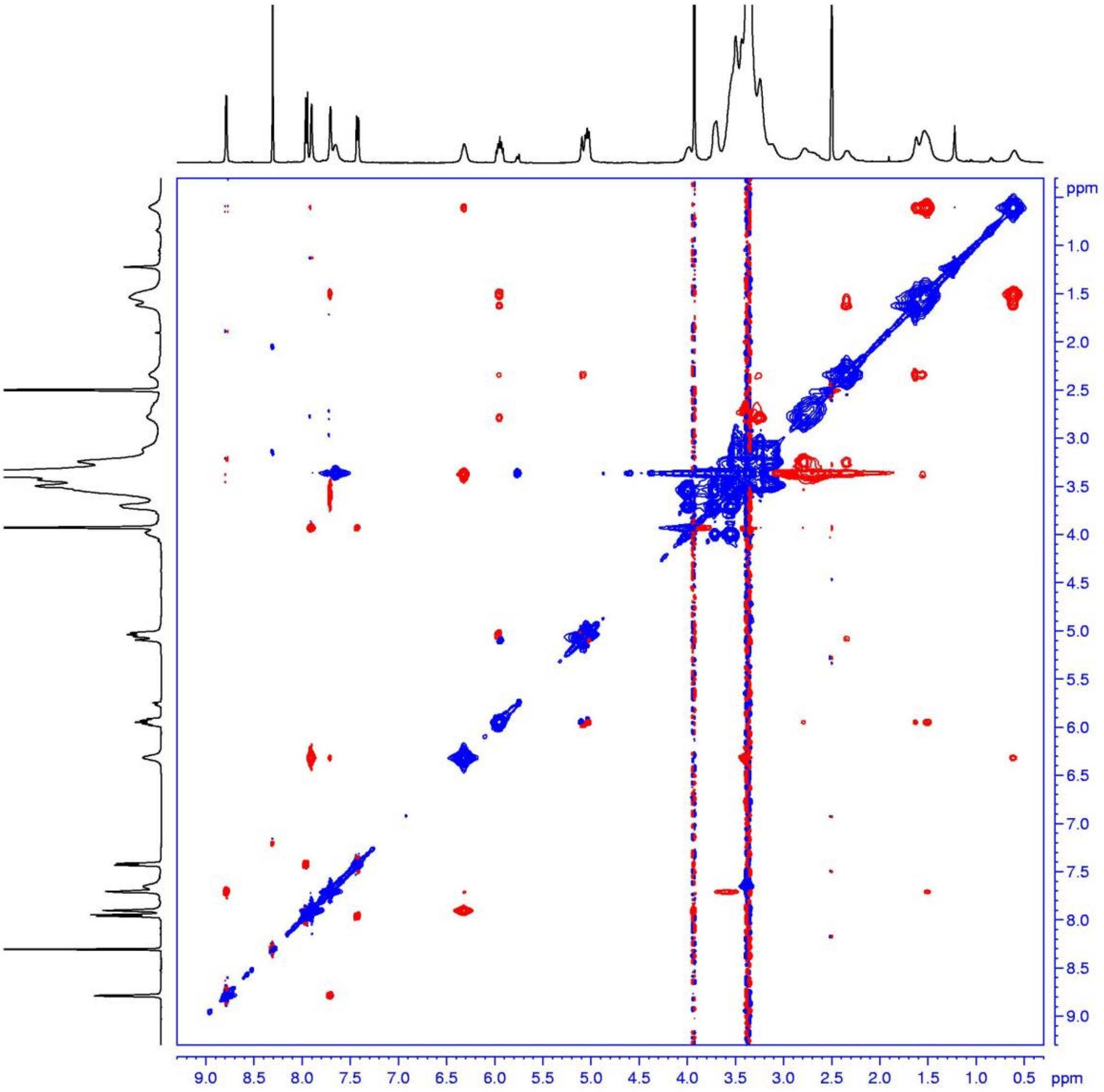

Figure S42. $^{1}H,^{1}H$-gs-ROESY NMR spectrum of intermediate **Q6** (DMSO-d_6)

NMR spectra of catalyst **HQ5**

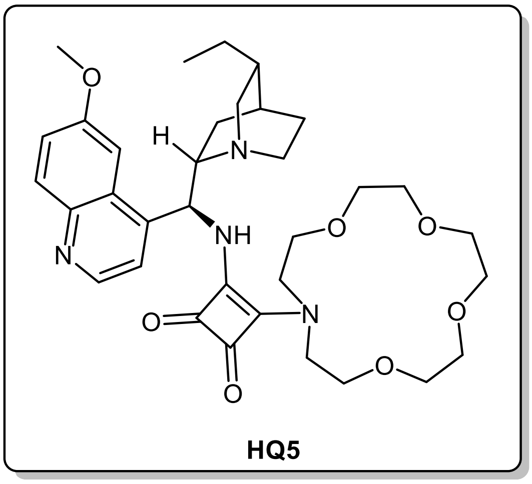

Figure S43. Molecule structure of catalyst **HQ5**

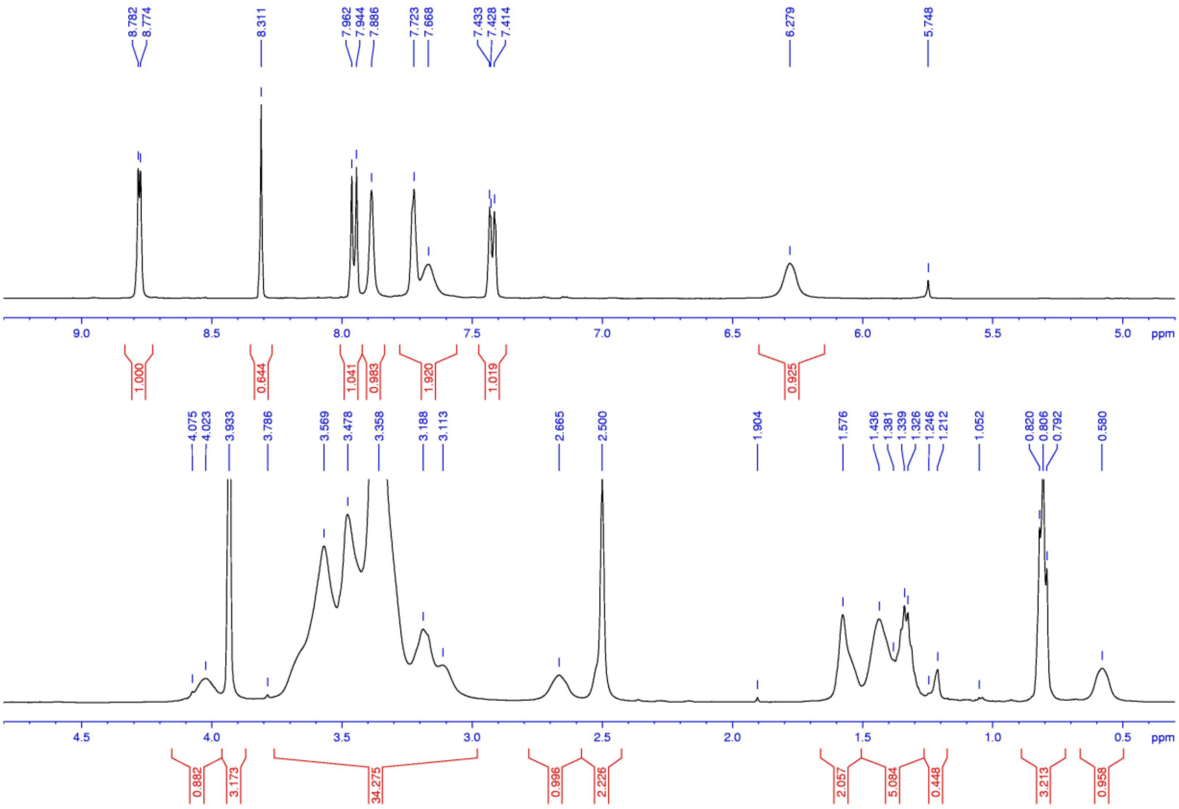

Figure S44. ^{1}H NMR spectrum of catalyst **HQ5** (500.13 MHz, DMSO-d_6)

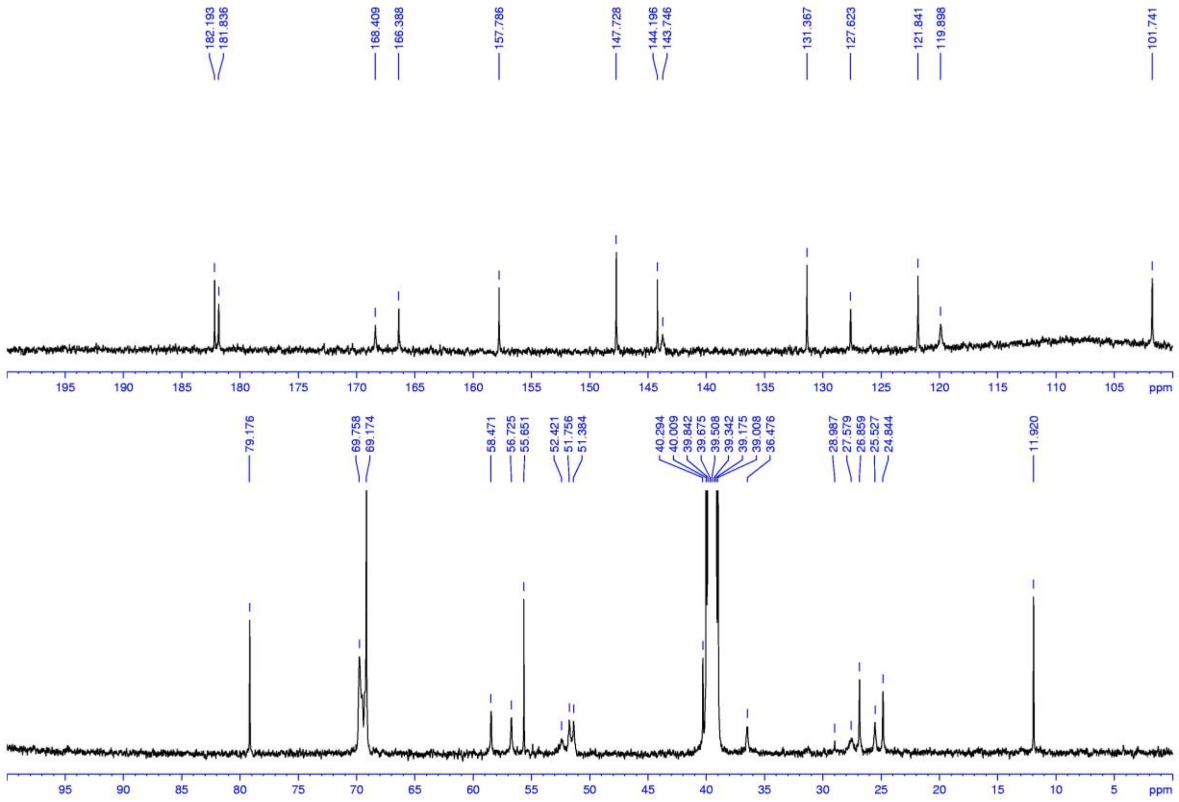

Figure S45. ^{13}C NMR spectrum of catalyst **HQ5** (125.76 MHz, DMSO-d_6)

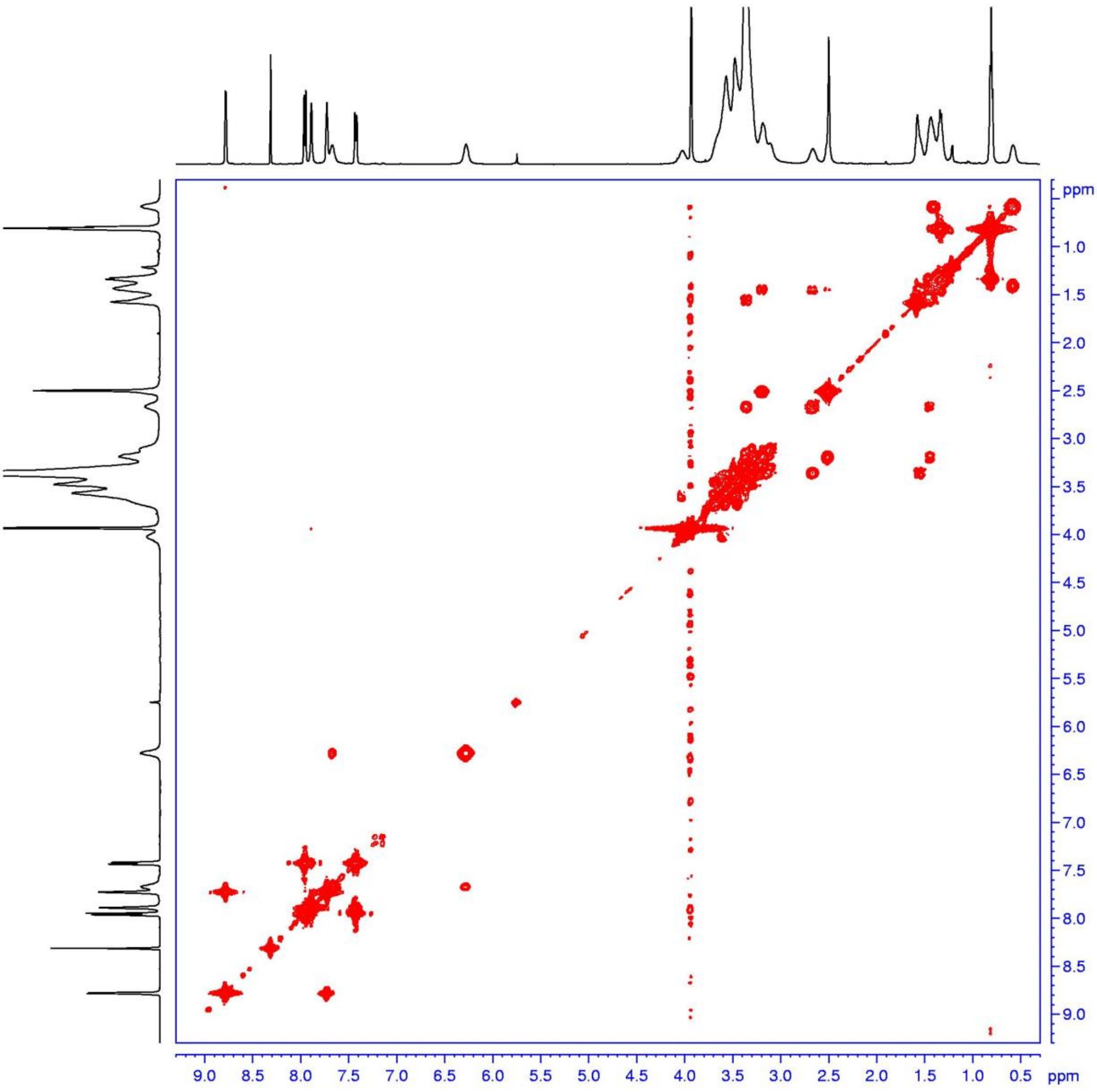

Figure S46. ^{1}H-gs-COSY NMR spectrum of catalyst **HQ5** (DMSO-d_6)

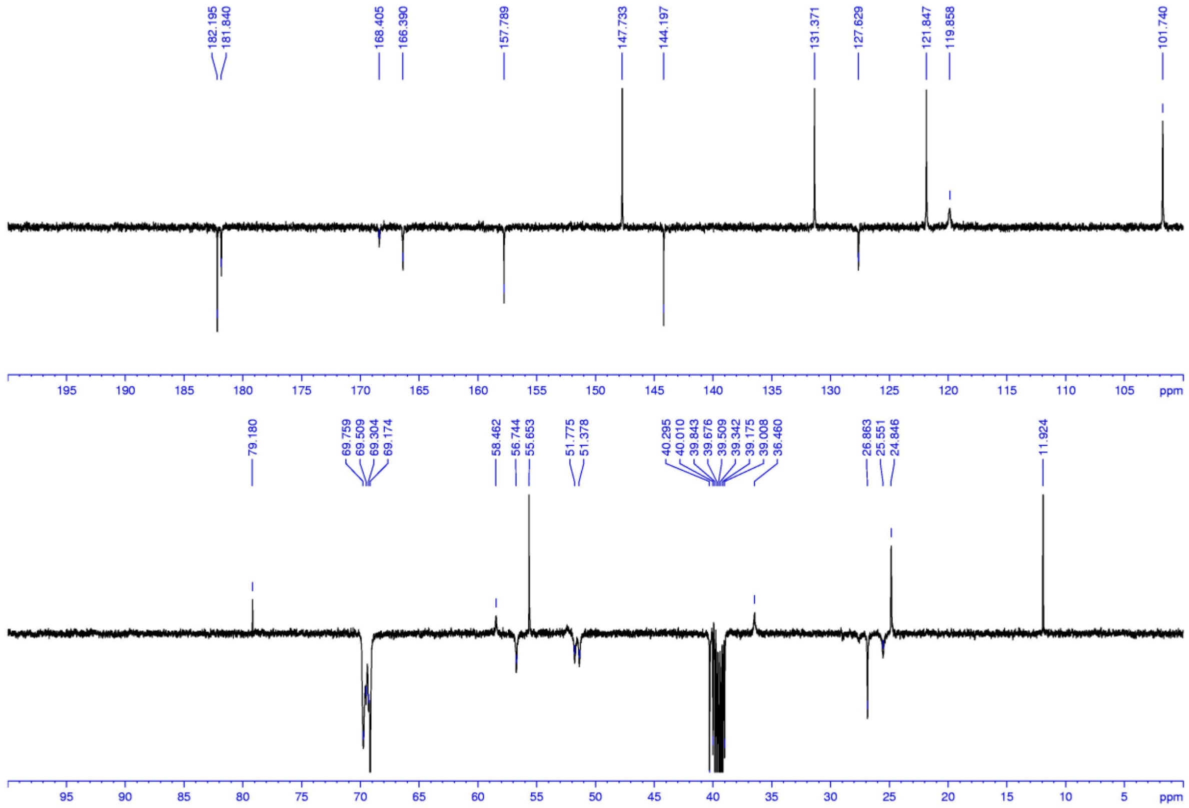

Figure S47. ^{13}C DEPTQ NMR spectrum of catalyst **HQ5** (DMSO-d_6)

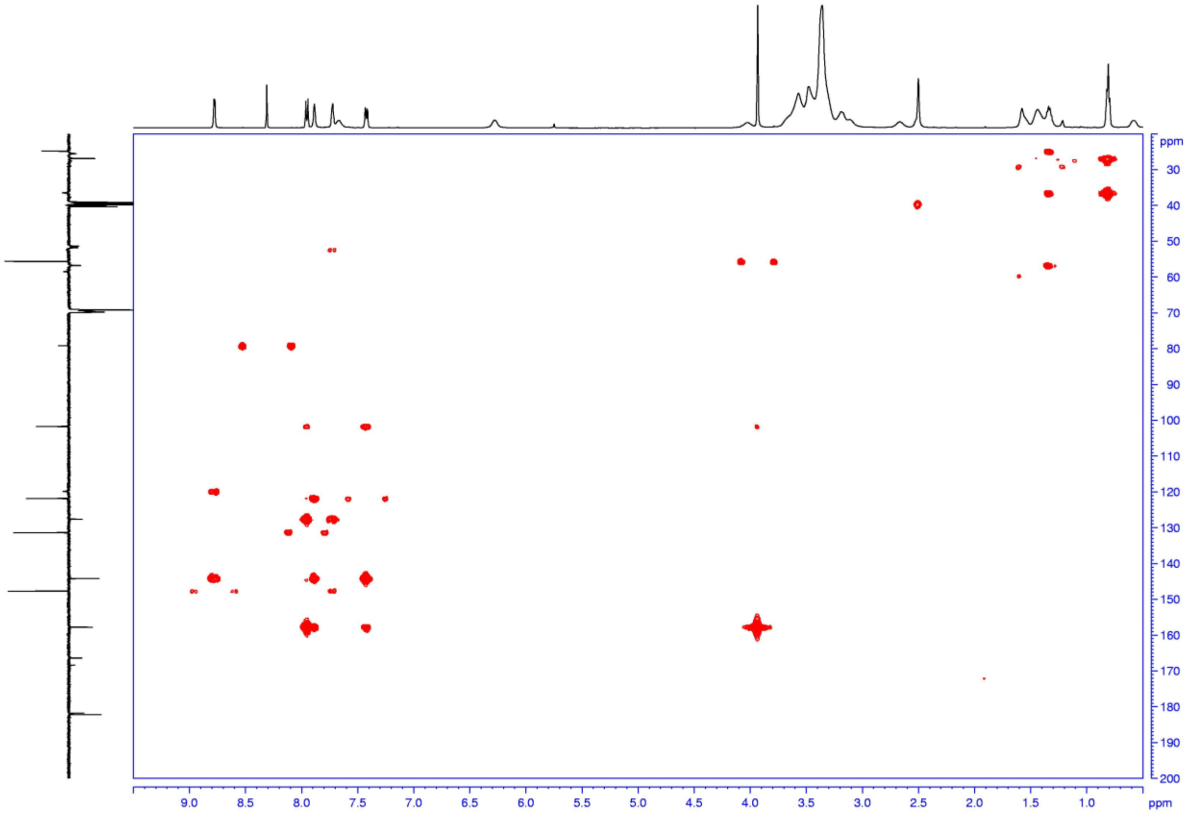

Figure S48. ^{1}H,^{13}C-gs-HMBC NMR spectrum of catalyst **HQ5** (DMSO-d_6)

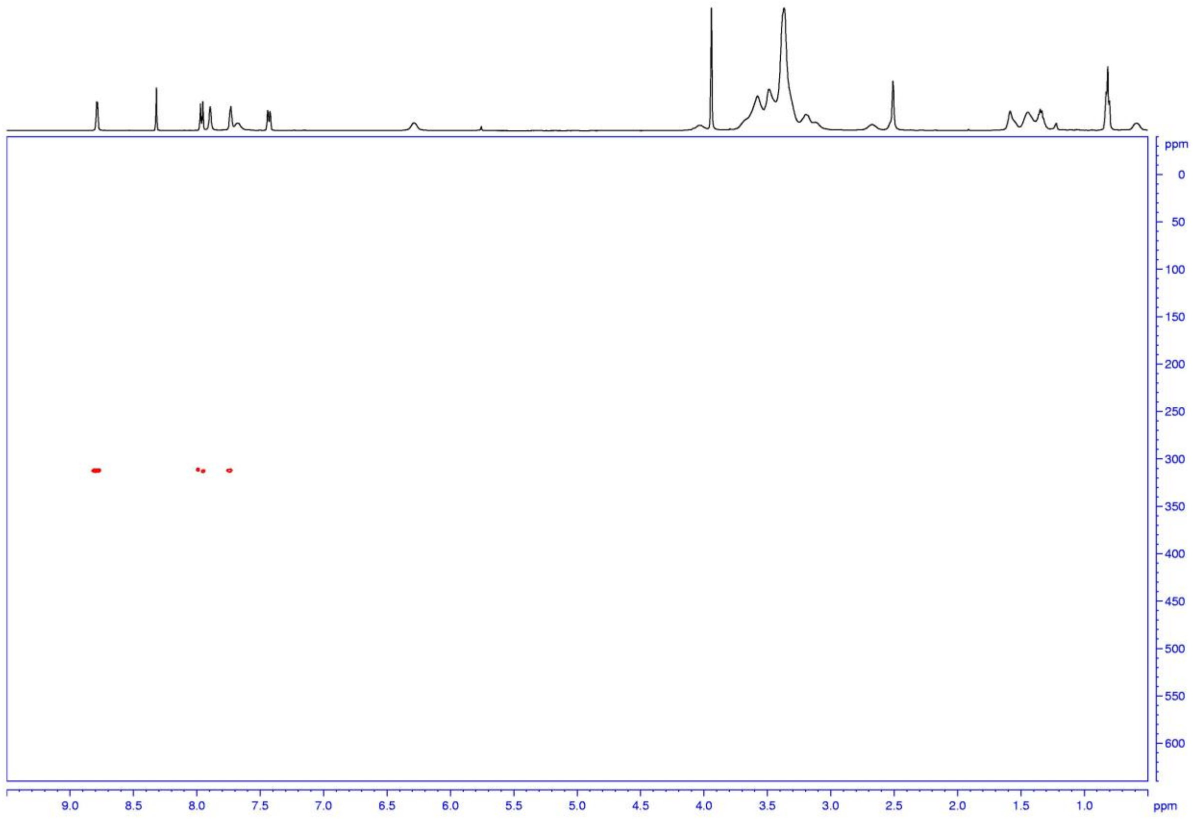

Figure S49. $^{1}H,^{15}N$-gs-HMBC NMR spectrum of catalyst **HQ5** (DMSO-d_6)

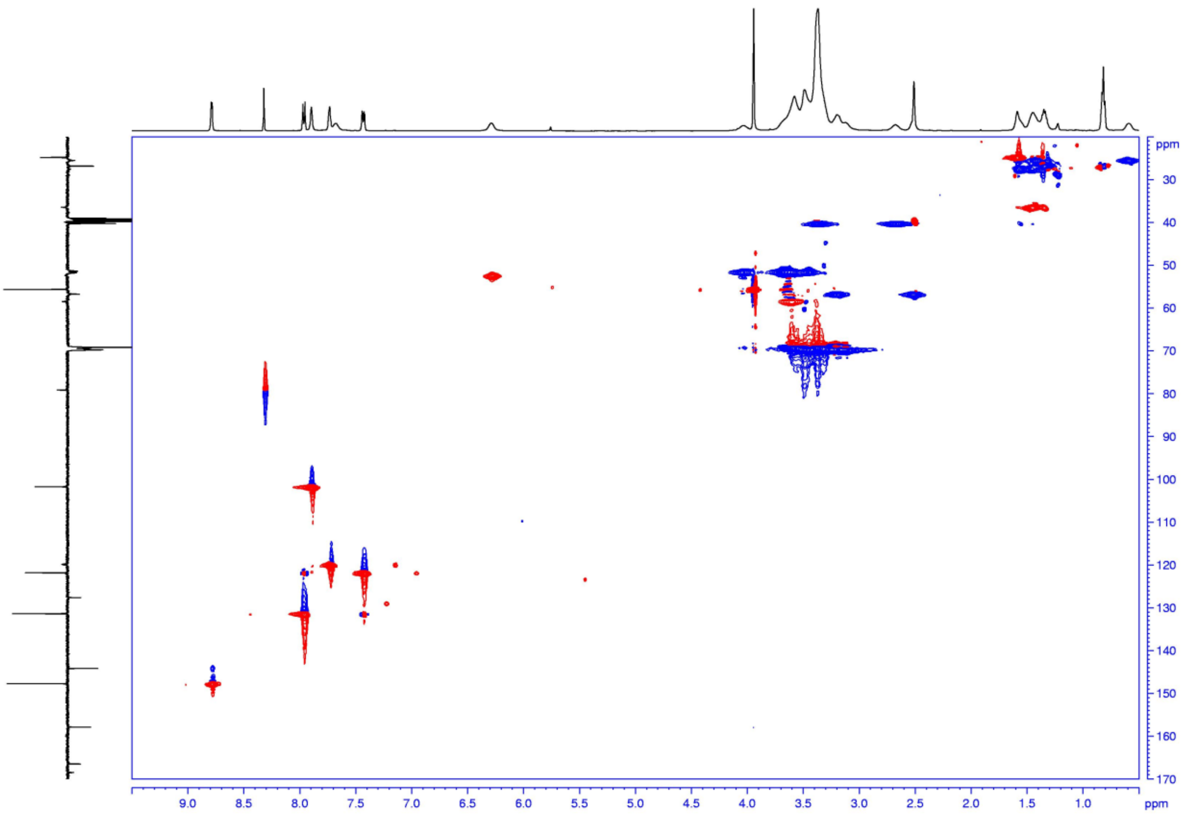

Figure S50. $^{1}H,^{13}C$-gs-HSQC NMR spectrum of intermediate **HQ5** (DMSO-d_6)

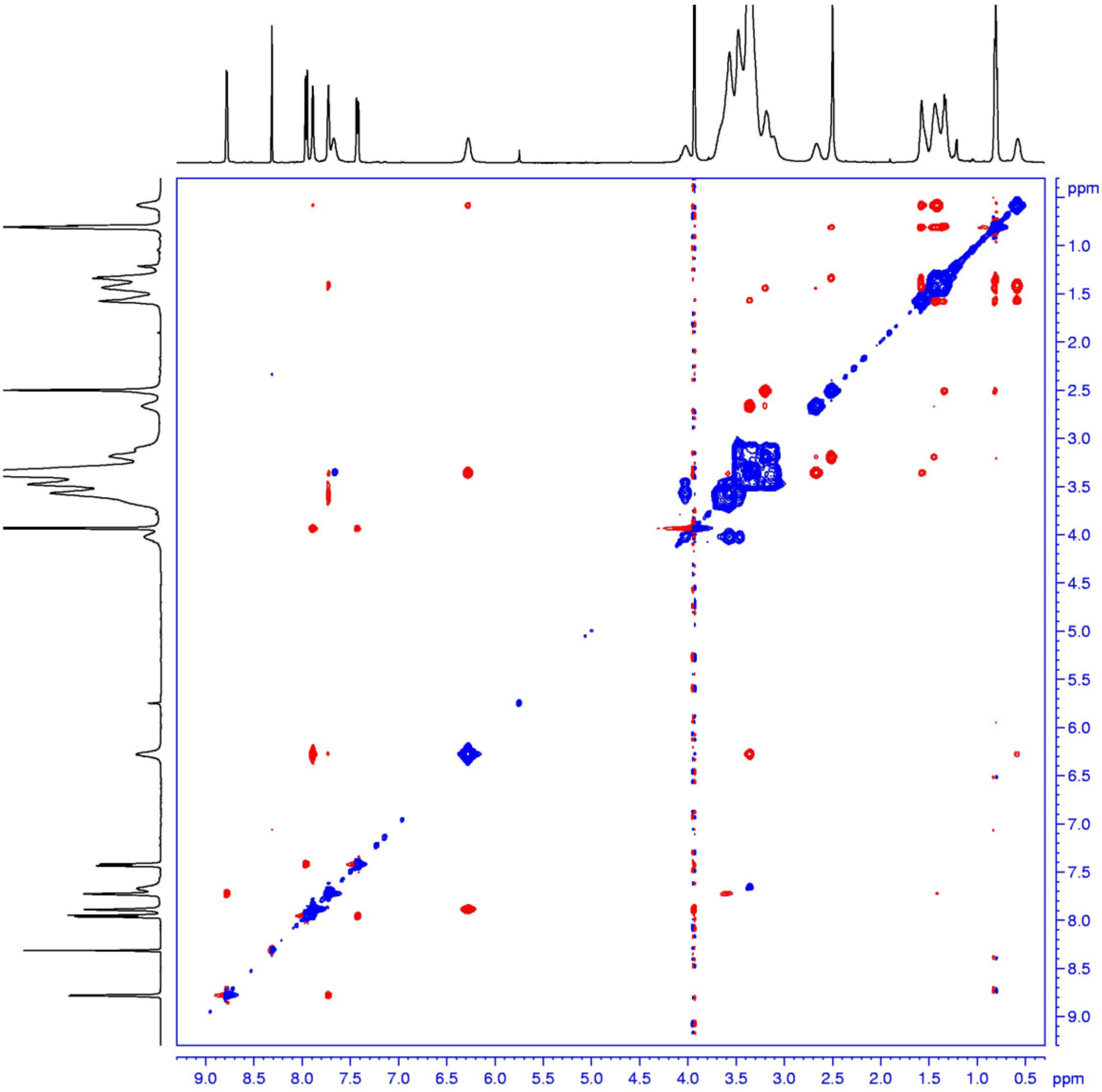

Figure S51. $^{1}H,^{1}H$-gs-ROESY NMR spectrum of intermediate **HQ5** (DMSO-d_6)

NMR spectra of catalyst **G5**

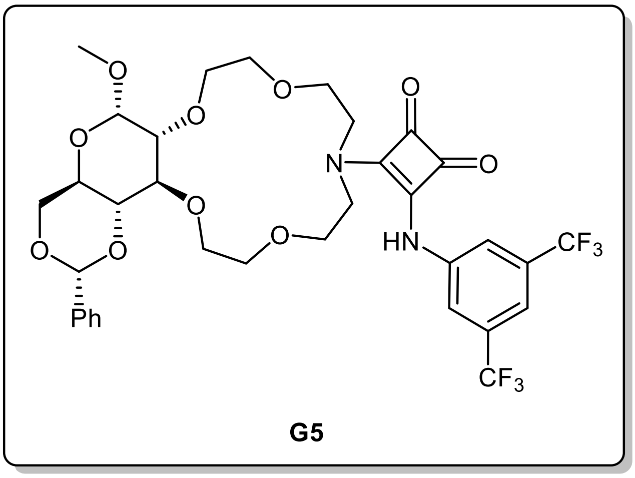

Figure S52. Molecule structure of catalyst **G5**

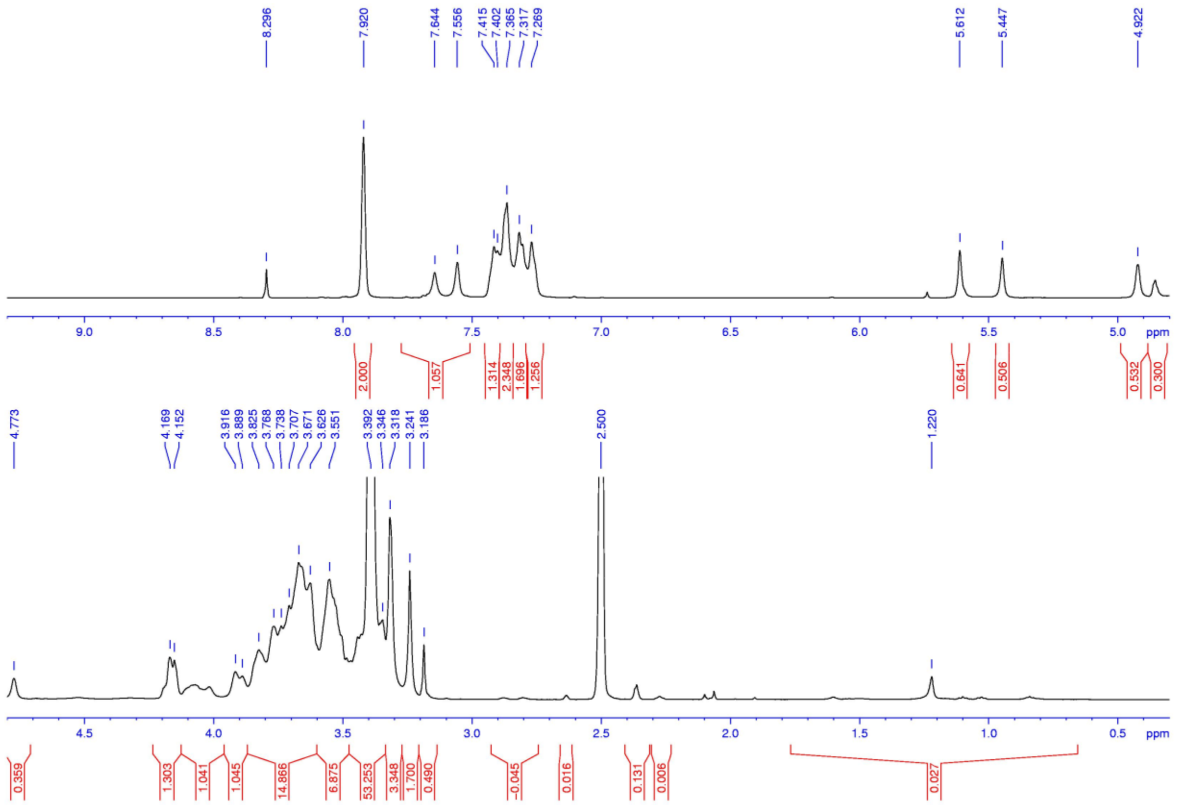

Figure S53. ^{1}H NMR spectrum of catalyst **G5** (500.13 MHz, DMSO-d_6)

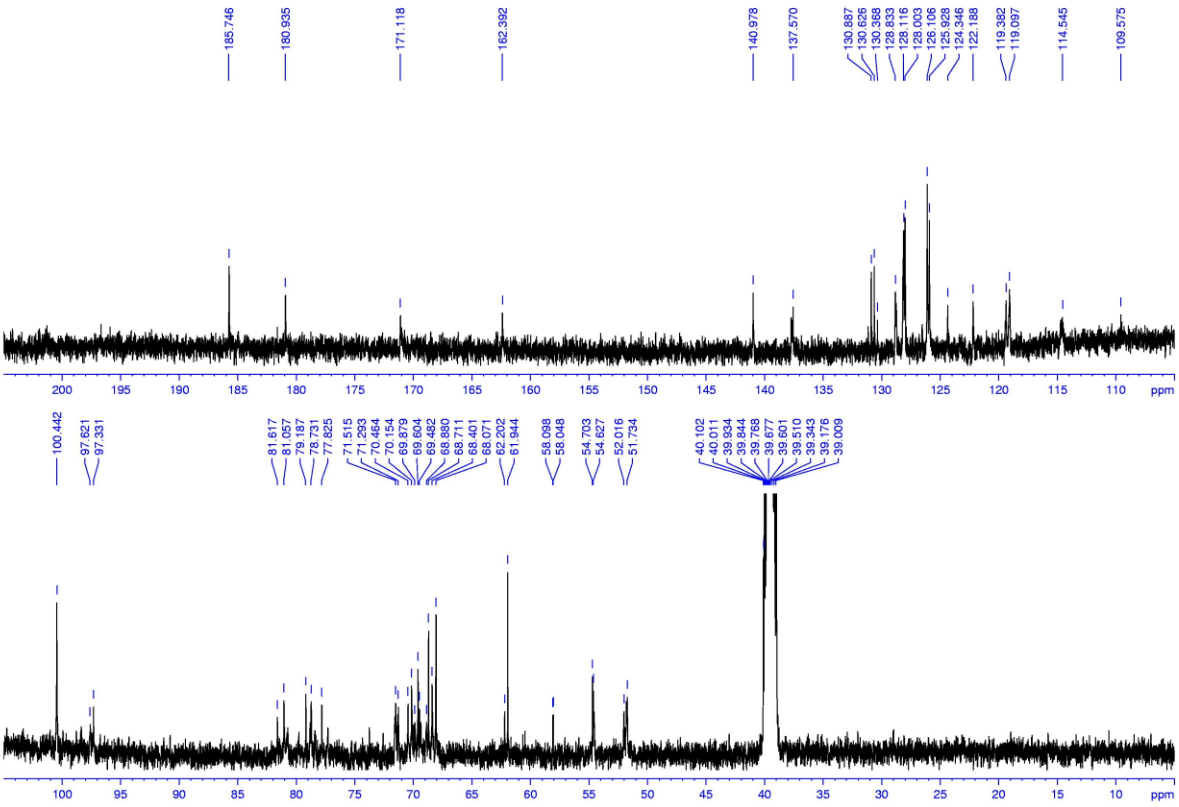

Figure S54. ^{13}C NMR spectrum of catalyst **G5** (125.76 MHz, DMSO-d_6)

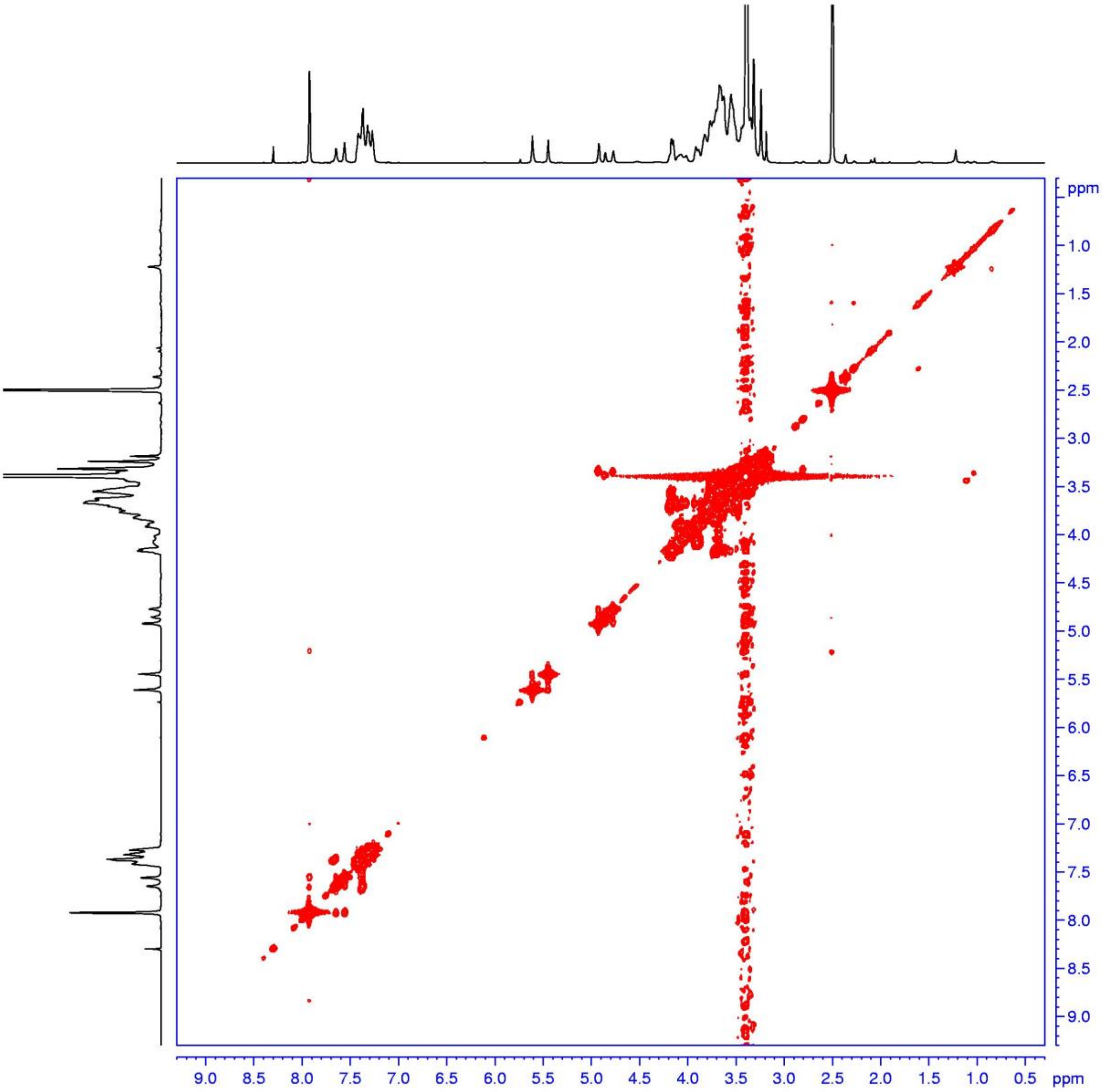

Figure S55. ^{1}H-gs-COSY NMR spectrum of catalyst **G5** (DMSO-d_6)

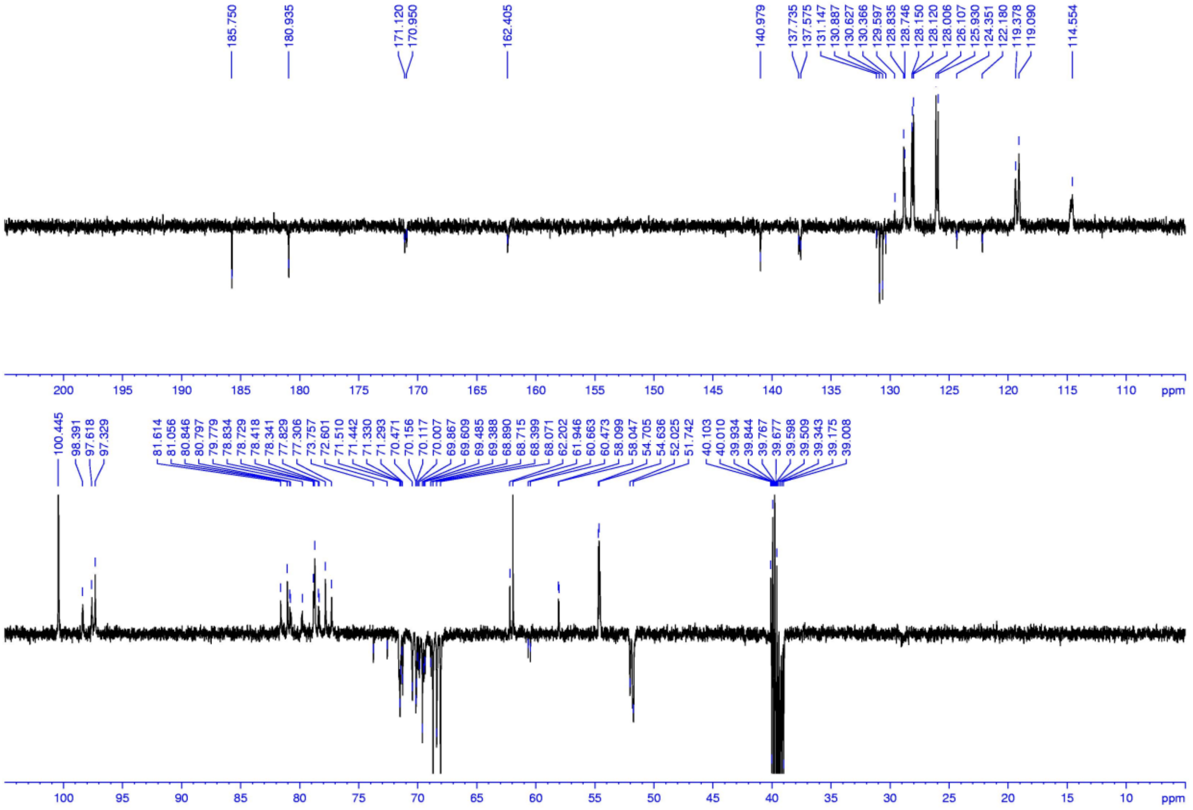

Figure S56. ^{13}C DEPTQ NMR spectrum of catalyst **G5** (DMSO-d_6)

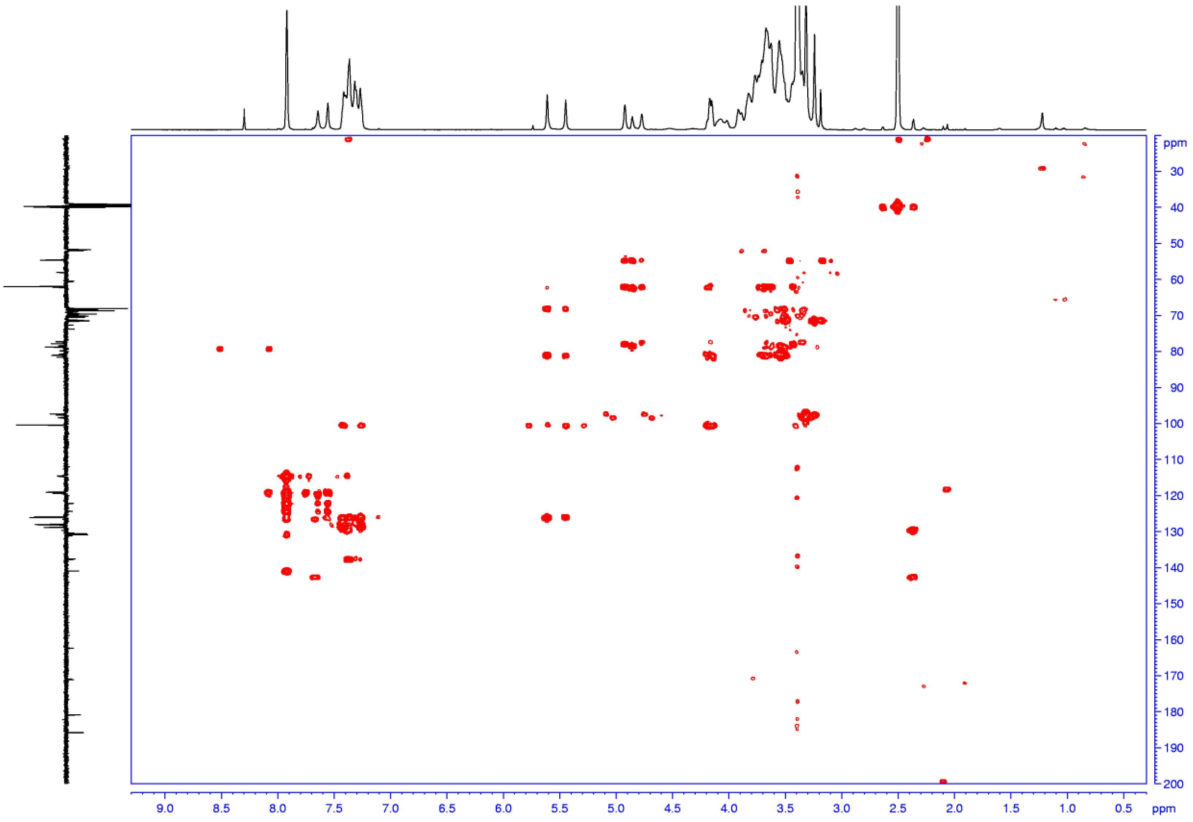

Figure S57. ^{1}H,^{13}C-gs-HMBC NMR spectrum of catalyst **G5** (DMSO-d_6)

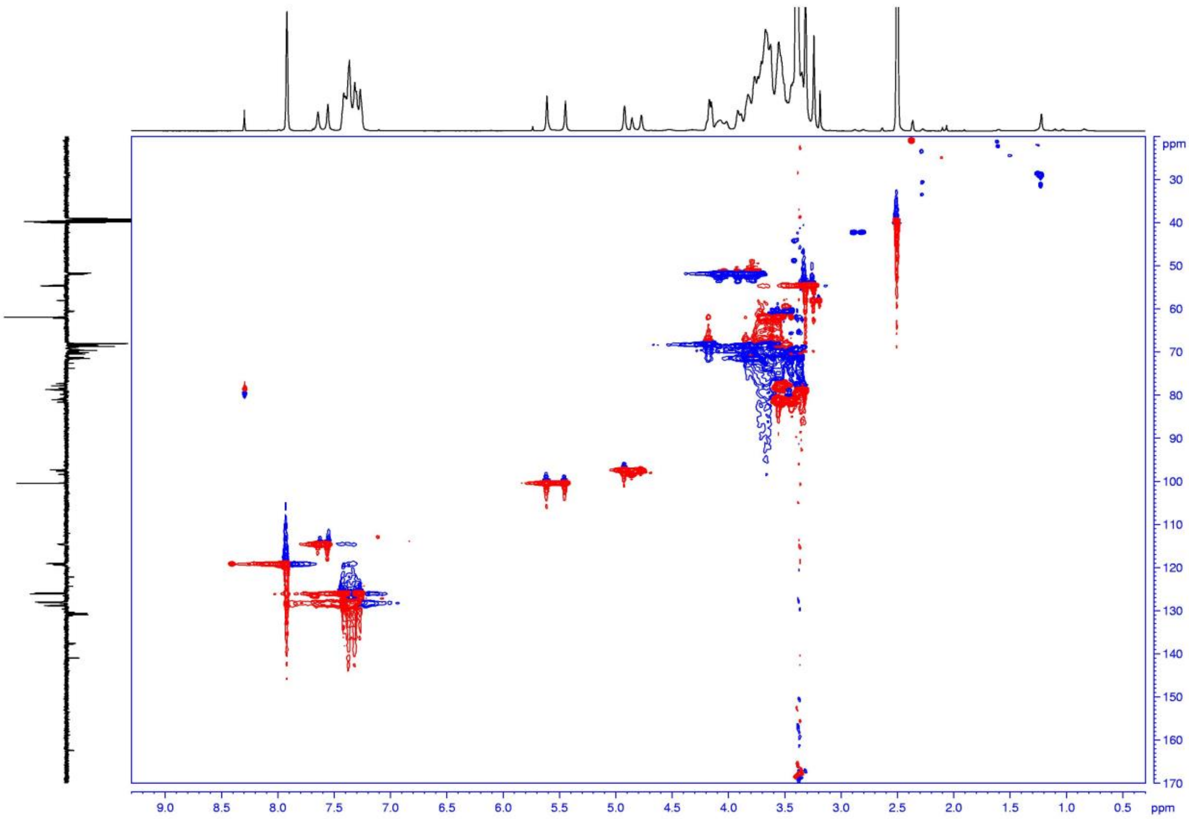

Figure S58. $^{1}H,^{13}C$-gs-HSQC NMR spectrum of intermediate **G5** (DMSO-d_6)

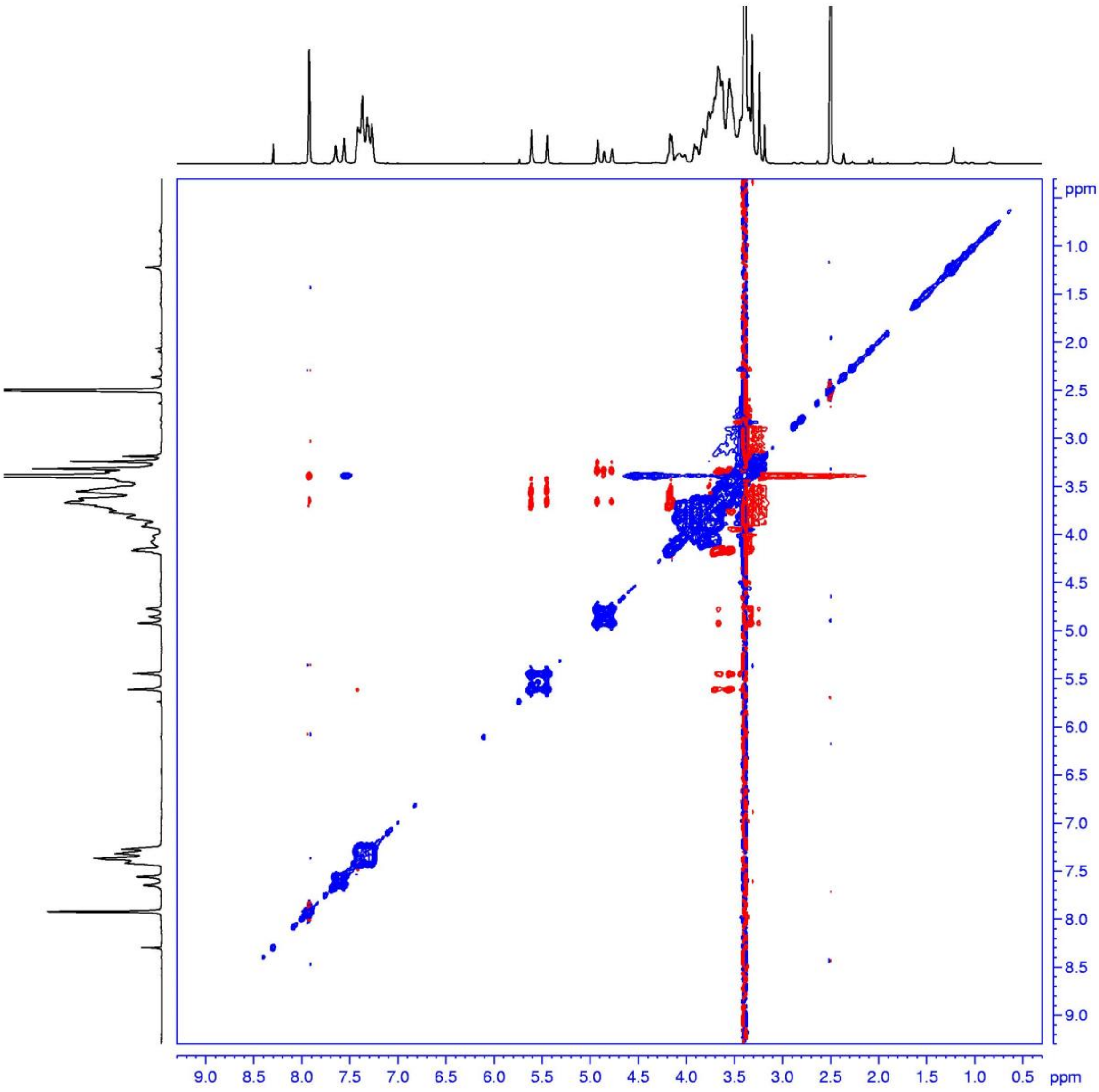

Figure S59. $^{1}H,^{1}H$-gs-ROESY NMR spectrum of intermediate **G5** (DMSO-d_6)

2. HPLC chromatograms

Product **3** of α-alkylation reaction

Method: Phenomenex Lux® 5 µm Cellulose-2 (250 × 4.6 mm ID), hexane/ethanol 95:5, 0.8 mL min^{-1}, 20 °C, λ=254 nm

HPLC chromatogram of racemic mixture

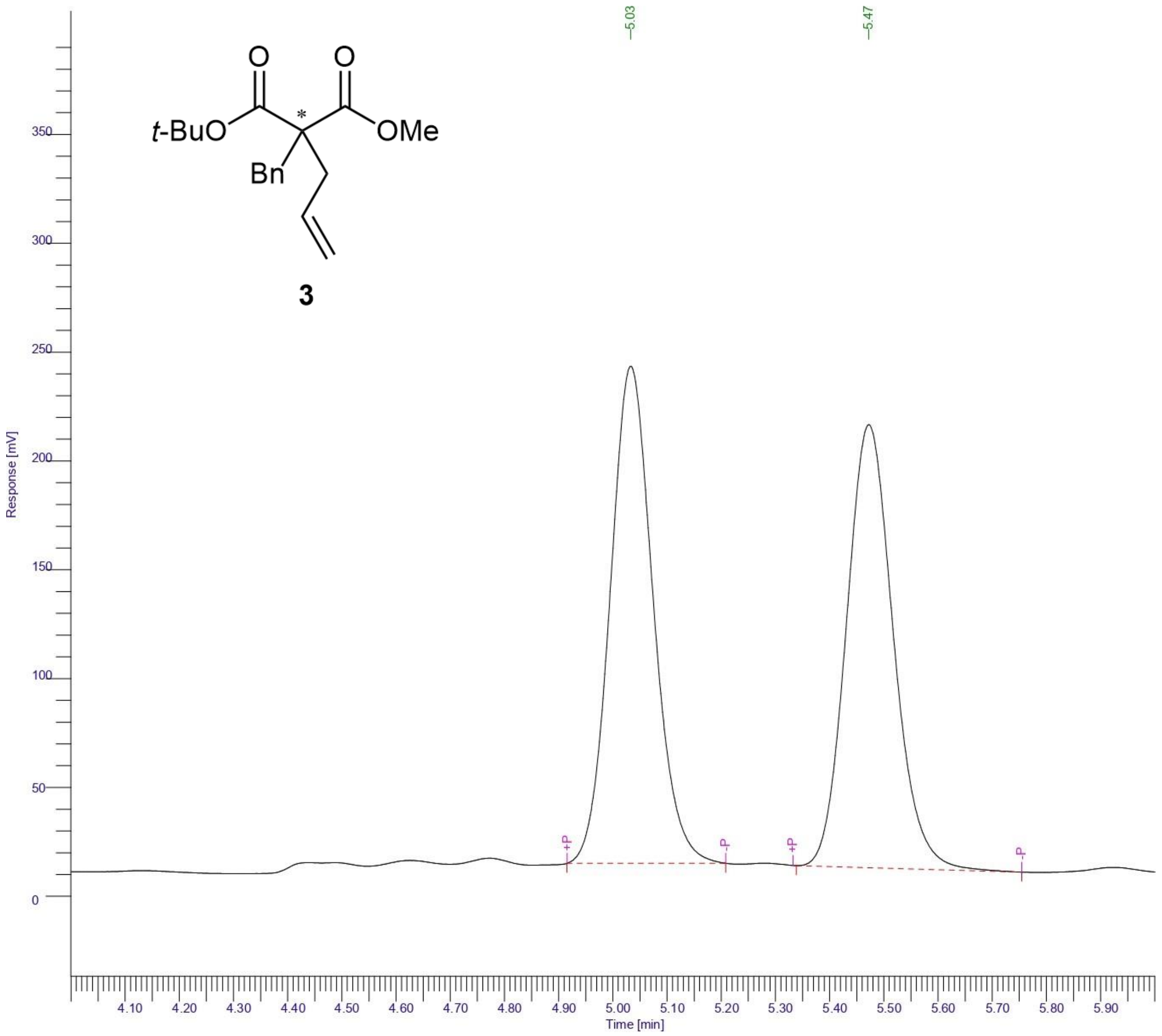

Figure S60. HPLC chromatogram of the racemic mixture of product **3**

Table S1. HPLC chromatogram peaks of the racemic mixture of product **3**

Peak #	Time [min]	Area [µV·s]	Height [µV]	Area [%]
1	5.033	1255114.36	228333.54	50.70
2	5.472	1220626.56	203554.28	49.30
		2475740.92	431887.81	100.00

HPLC chromatogram of product **3** with catalyst **C5** (17% ee)

Reaction conditions: *tert*-butyl methyl α-benzylmalonate (1 eq), allyl iodide reagent (1.2 eq), **C5** catalyst (10 mol%), 50% aq. KOH base (50 eq), 2.4 mL DCM solvent, 0 °C, 24 h reaction time.

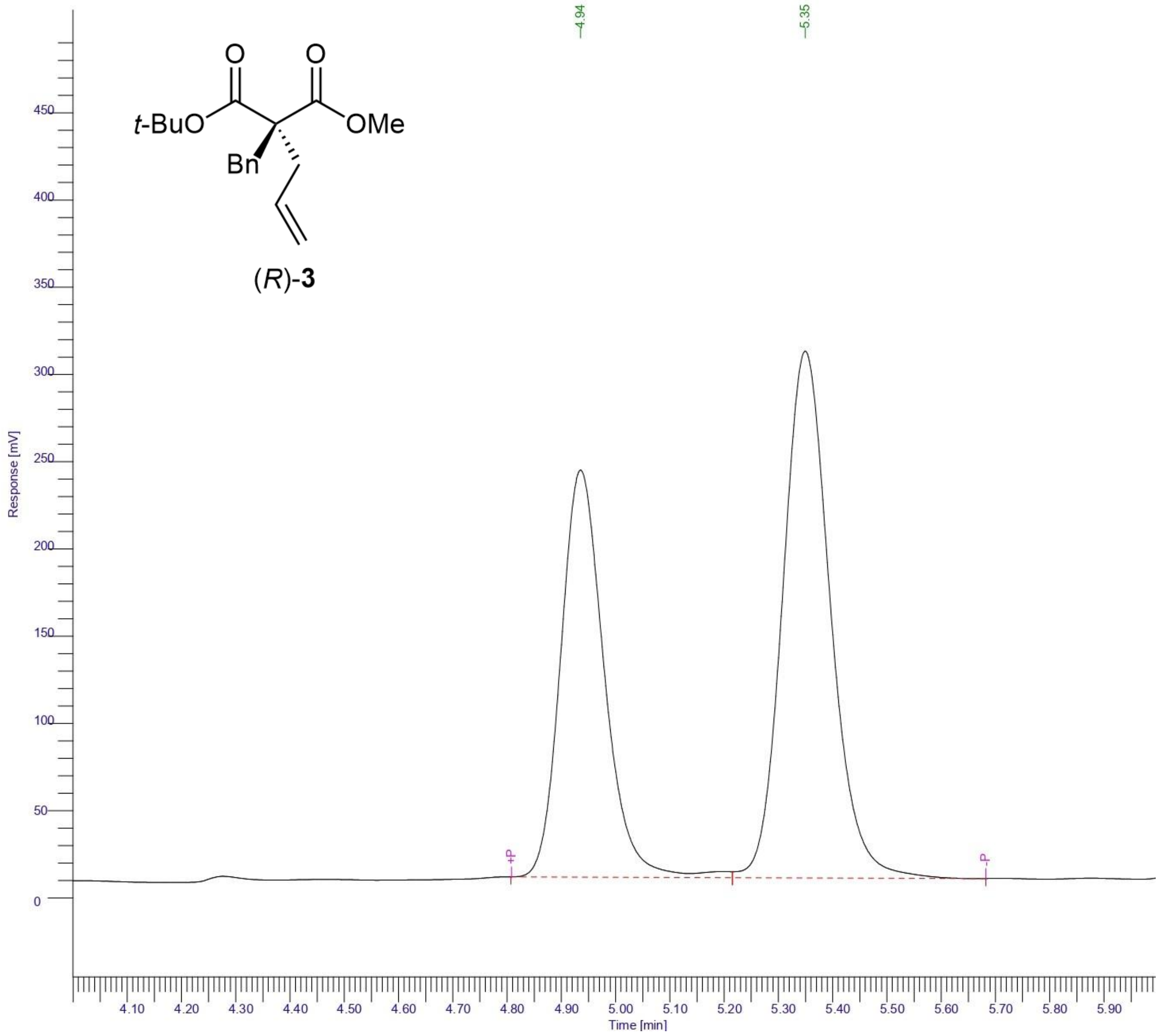

Figure S61. HPLC chromatogram of product (*R*)-**3** (17% ee)

Table S2. HPLC chromatogram peaks of product (*R*)-**3** (17% ee)

Peak #	Time [min]	Area [μV·s]	Height [μV]	Area [%]
1	4.935	1299138.74	233277.90	41.47
2	5.349	1833377.33	301912.66	58.53
		3132516.08	535190.57	100.00

Product **5** of *Michael* addition reaction

Method: Kromasil® 5 µm AmyCoat® (250 × 4.6 mm ID), hexane/ethanol 85:15, 0.8 mL min^{-1}, 20 °C, λ=254 nm

HPLC chromatogram of racemic mixture

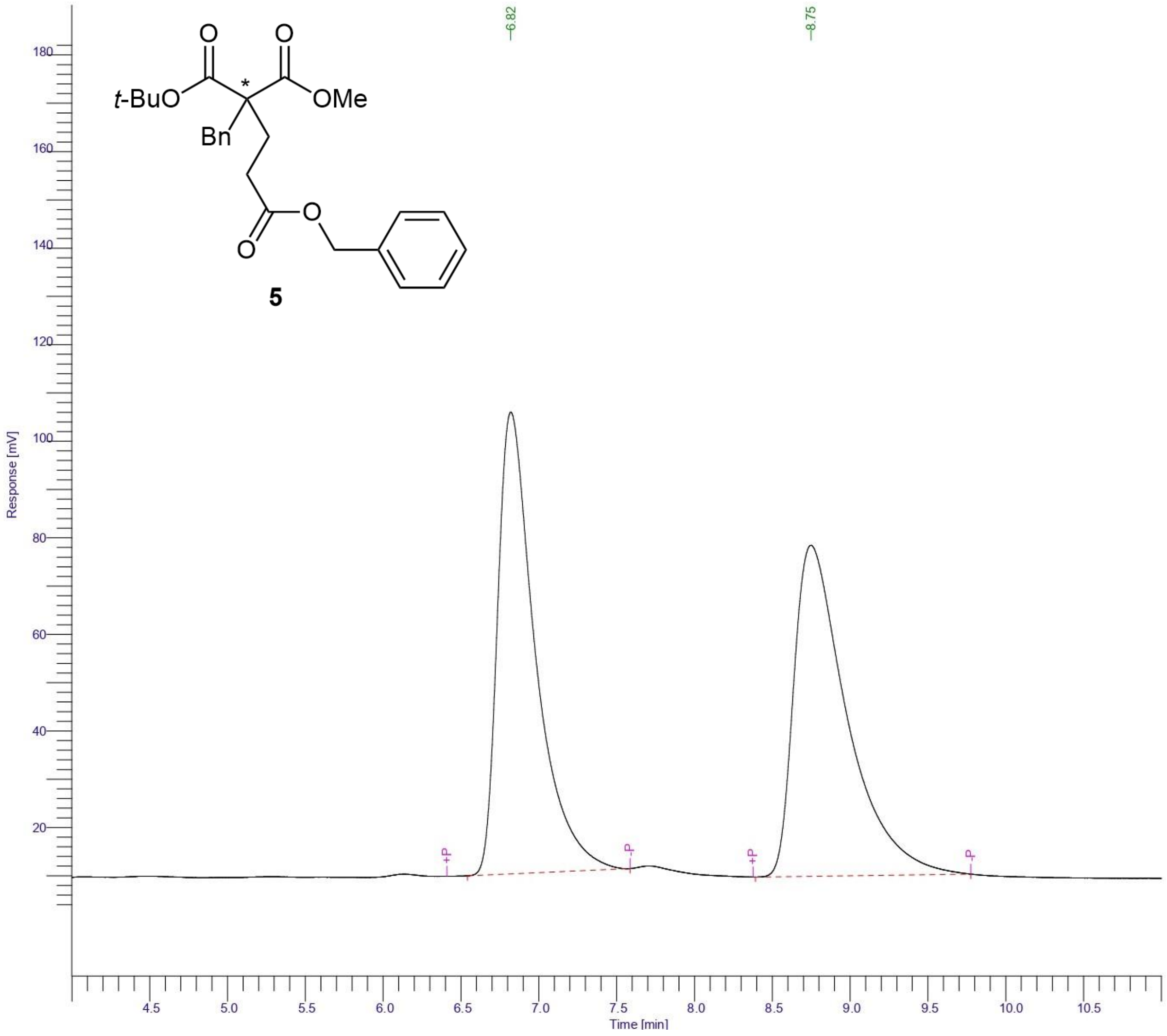

Figure S62. HPLC chromatogram of the racemic mixture of product **5**

Table S3. HPLC chromatogram peaks of the racemic mixture of product **5**

Peak #	Time [min]	Area [µV·s]	Height [µV]	Area [%]
1	6.820	1609853.73	95671.16	49.79
2	8.749	1623189.02	68560.60	50.21
		3233042.75	164231.76	100.00
